# Karyosystematics and molecular taxonomy of the anomalous blue butterflies (Lepidoptera, Lycaenidae) from the Balkan Peninsula

**DOI:** 10.3897/CompCytogen.v10i5.10944

**Published:** 2016-12-20

**Authors:** Maria S. Vishnevskaya, Alsu F. Saifitdinova, Vladimir A. Lukhtanov

**Affiliations:** 1 Department of Karyosystematics, Zoological Institute of Russian Academy of Sciences, Universitetskaya nab. 1, 199034 St. Petersburg, Russia; 2 Department of Entomology, St Petersburg State University, Universitetskaya nab. 7/9, 199034 St. Petersburg, Russia; 3 Department of Cytology and Histology, St Petersburg State University, Universitetskaya nab. 7/9, 199034 St. Petersburg, Russia

**Keywords:** Agrodiaetus, biodiversity, chromosome, *COI*, conservation, cryptic species, DNA barcode, *ITS2*, karyotype, mitochondrial marker, Polyommatus timfristos sp. n., protected species, red list

## Abstract

The Balkan Peninsula represents one of the hottest biodiversity spots in Europe. However, the invertebrate fauna of this region is still insufficiently investigated, even in respect of such well-studied organisms as Lepidoptera. Here we use a combination of chromosomal, molecular and morphological markers to rearrange the group of so-called anomalous blue butterflies (also known as ‘brown complex’ of the subgenus Agrodiaetus Hübner, [1822] and as the Polyommatus (Agrodiaetus) admetus (Esper, 1783) species group) and to reveal its cryptic taxonomic structure. We demonstrate that *Polyommatus aroaniensis* (Brown, 1976) is not as widespread in the Balkans as was previously thought. In fact, it has a dot-like distribution range restricted to the Peloponnese Peninsula in South Greece. *Polyommatus orphicus* Kolev, 2005 is not as closely related to the Turkish species *Polyommatus dantchenkoi* (Lukhtanov & Wiemers, 2003) as was supposed earlier. Instead, it is a Balkan endemic represented by two subspecies: *Polyommatus orphicus orphicus* (Bulgaria) and *Polyommatus orphicus eleniae* Coutsis & De Prins, 2005 (Northern Greece). *Polyommatus ripartii* (Freyer, 1830) is represented in the Balkans by an endemic subspecies *Polyommatus ripartii pelopi*. The traditionally recognized *Polyommatus admetus* (Esper, 1783) is shown to be a heterogeneous complex and is divided into *Polyommatus admetus* sensu stricto (the Balkans and west Turkey) and *Polyommatus yeranyani* (Dantchenko & Lukhtanov, 2005) (east Turkey, Armenia, Azerbaijan and Iran). *Polyommatus nephohiptamenos* (Brown & Coutsis, 1978) is confirmed to be a species with a dot-like distribution range in Northern Greece. Finally, from Central Greece (Timfristos and Parnassos mountains) we describe *Polyommatus timfristos* Lukhtanov, Vishnevskaya & Shapoval, **sp. n.** which differs by its haploid chromosome number (n=38) from the closely related and morphologically similar *Polyommatus aroaniensis* (n=47-48) and *Polyommatus orphicus* (n=41-42). We provide chromosomal evidence for three separate south Balkan Pleistocene refugia (Peloponnesse, Central Greece and Northern Greece/South Bulgaria) and stress the biogeographic importance of Central Greece as a place of diversification. Then we argue that the data obtained have direct implications for butterfly karyology, taxonomy, biogeography and conservation.

## Introduction

The Balkan Peninsula is recognized as a European biodiversity hotspot, with high endemism found in animals and plants (Nicolić et al. 2014, Buj et al. 2015, Bregović and Zagmajster 2016). However, the invertebrate fauna of this region is still insufficiently investigated (Previšić et al. 2016), even in respect of such a well-studied group as Lepidoptera (butterflies and moths) (Sobezyk and Gligorović 2016).

Within Balkan Lepidoptera, the *Agrodiaetus* Hübner, [1822] blue butterflies are the most complicated group from the taxonomical point of view. The subgenus Agrodiaetus is a distinct monophyletic lineage within the species-rich genus *Polyommatus* Latreille, 1804 (Talavera et al. 2013a). Adult *Agrodiaetus* butterflies are small in size with wing span from 1.9 to 3.6 cm. Females are mostly warm brown on the upperside of the wings, whereas males can be either blue or brown. In the latter case, they resemble females. Thus, a species can be classified as either dimorphic or monomorphic depending on the wing color of the males. Most of the *Agrodiaetus* species have a white streak on the underside of hind wings, and this feature appears to be an apomorphic character of the subgenus Agrodiaetus. However, in a few species and populations this white streak is secondarily reduced or totally absent (Eckweiler and Bozano 2016).

The subgenus Agrodiaetus includes numerous species, subspecies and forms with uncertain taxonomic positions (Eckweiler and Häuser 1997). It was estimated to have originated only about 3 million years ago (Kandul et al. 2004) and radiated rapidly in the Western Palaearctic (Kandul et al. 2007). The last published review of the subgenus includes 120 valid species (Eckweiler and Bozano 2016). Many of them have extremely local ‘dot-like’ distributions that are restricted to particular mountain valleys in the Balkan Peninsula, Asia Minor, Transcaucasus, Iran and Central Asia (Vila et al. 2010, Eckweiler and Bozano 2016).

Although this group has attracted the attention of numerous researchers (e.g. de Lesse 1960a, b, Häuser and Eckweiler 1997, Oliver et al. 1999, Carbonell 2000, 2001, Dantchenko 2000, Przybyłowicz 2000, 2014, ten Hagen and Eckweiler 2001, Skala 2001, Lukhtanov and Dantchenko 2002a, Kandul et al. 2002, 2004, 2007, Wiemers 2003, Schurian and ten Hagen 2003, Vila et al. 2010, Talavera et al. 2013a), a large number of unresolved taxonomic problems still persist in *Agrodiaetus*.

In most cases, species identification in *Agrodiaetus* is extremely difficult. The morphology of male genitalia is uniform throughout most of the species and, with a few exceptions (see Coutsis 1985, 1986), at most it can help to separate groups of species, e.g. the *Polyommatus dolus* (Hübner, 1823) and *Polyommatus admetus* (Esper, 1783) species groups (see Kolev 2005), but not individual species. The differences in wing pattern and coloration between many *Agrodiaetus* species are very subtle or nearly lacking (Eckweiler and Bozano 2016).

Despite morphological similarity, the taxonomic and identification problems within the subgenus Agrodiaetus can be solved if chromosomal (de Lesse 1960a,b, Lukhtanov 1989) or molecular markers (Wiemers 2003, Kandul et al. 2004, 2007, Lukhtanov et al. 2005, Stradomsky and Fomina 2013), or their combination (Lukhtanov et al. 2006, 2014, 2015a, Vila et al. 2010, Lukhtanov and Tikhonov 2015, Shapoval and Lukhtanov 2015a,b) are applied. Although chromosome numbers are invariable in many groups of Lepidoptera (Robinson 1971, Lukhtanov 2014, Hernández-Roldán 2016), a few genera demonstrate chromosomal instability, a situation in which multiple closely related species differ drastically from each other by major chromosomal rearrangements, sometimes resulting in high variability in chromosome number (de Lesse 1960a,b, Talavera et al. 2013b). An unusual diversity of karyotypes is the most remarkable characteristic of the subgenus Agrodiaetus. Species of *Agrodiaetus* exhibit one of the highest ranges in chromosome numbers in the animal kingdom (Lukhtanov 2015). Haploid chromosome numbers in *Agrodiaetus* range from n=10 in *Agrodiaetus caeruleus* (Staudinger, 1871) to n=134 in *Agrodiaetus shahrami* (Skala, 2001) (Lukhtanov and Dantchenko 2002a, Lukhtanov et al. 2005). Additionally, this subgenus demonstrates a high level of karyotypic differentiation with respect to chromosome size (Lukhtanov and Dantchenko 2002b) and variation in number of chromosomes bearing ribosomal DNA clusters (Vershinina et al. 2015). The karyotype is generally stable within species although differences between closely related taxa are often high and provide reliable characters for species delimitation, description and identification (de Lesse 1960a,b, Lukhtanov and Dantchenko 2002a,b).

Molecular studies revealed that subgenus Agrodiaetus consists of 10 monophyletic clades: the *Polyommatus transcaspicus* (Heyne, 1895) group, the *Polyommatus iphigenides* (Staudinger, 1886) group, the *Polyommatus ershoffii* (Lederer, 1869) group, the *Polyommatus poseidon* (Herrich-Schäffer, 1844) group, the *Polyommatus admetus* group, the *Polyommatus damone* (Eversmann, 1841) group, the *Polyommatus carmon* (Herrich-Schäffer, 1851) group, the *Plebejus damon* (Denis & Schiffermüller, 1775) group, the *Polyommatus dolus* group and the *Polyommatus actis* (Herrich-Schäffer, 1851) group (Kandul et al. 2002, 2004, 2007, Wiemers 2003). They also demonstrated that many species are clearly differentiated with respect to mitochondrial and nuclear DNA sequences. However, this is not a general rule, as the standard mitochondrial DNA barcodes are often identical or nearly identical between closely related taxa and even between morphologically distinct species (Kandul et al. 2004, 2007, Wiemers and Fiedler 2007). Generally, chromosomal characters in *Agrodiaetus* evolve more quickly than standard DNA barcodes, and because they are usually present as fixed differences, provide better markers for recently evolved taxa than nucleotide substitutions (Lukhtanov et al. 2015a).

Species delimitation is especially difficult within a group of so-called anomalous blue species (known also as ‘brown complex’ of the subgenus Agrodiaetus and as the *Polyommatus admetus* species complex). This group is composed of multiple species in which both male and female butterflies have similar brown coloration on the upperside of the wings (Lukhtanov et al. 2003).

The group of anomalous blue species includes taxa belonging to two clearly monophyletic and most probably sister clades: the *Polyommatus admetus* clade (comprises only monomorphic species – *Polyommatus admetus*, *Polyommatus demavendi*, *Polyommatus khorasanensis*. *Polyommatus nephohiptamenos*, *Polyommatus ripartii*, *Polyommatus pseudorjabovi*) and the *Polyommatus dolus* clade (comprises both monomorphic – *Polyommatus alcestis*, *Polyommatus karacetinae*, *Polyommatus eriwanensis*, *Polyommatus interjectus*, *Polyommatus dantchenkoi*, *Polyommatus humedasae*, *Polyommatus aroaniensis*, *Polyommatus orphicus*, *Polyommatus timfristos* sp. n., *Polyommatus fabressei*, *Polyommatus violetae*, *Polyommatus valiabadi*, *Polyommatus rjabovianus*; and dimorphic species – *Polyommatus dolus*, *Polyommatus fulgens*, *Polyommatus menalcas*). The anomalous blue butterflies represent a real stumbling block in the *Agrodiaetus* taxonomy (Lukhtanov et al. 2003, Przybyłowicz et al. 2014). According to Eckweiler and Bozano (2016), the group is distributed in West Palearctic from Spain in the west to Mongolia in the east. The majority of the species have very localized distribution areas concentrated in (1) the Iberian Peninsula, (2) the Balkan Peninsula and (3) west Asia (mostly in the Middle East and Caucasus). Vila et al. (2010) studied in detail the European *Agrodiaetus* taxa distributed west of the 17th meridian, using a combination of molecular and chromosomal markers (Vila et al. 2010). Chromosomal and molecular markers were also applied to study the taxonomy of the Asian taxa (Lukhtanov et al. 2015a). It is paradoxical that systematic studies based on combined analysis of molecular and chromosomal markers have never been applied to Balkan taxa of the *Polyommatus admetus* species complex. However, some DNA data can be found in GenBank (Wiemers 2003, Wiemers et al. 2007, 2009, 2010, Lukhtanov et al. 2009, 2015a, Vila et al. 2010, Dincă et al. 2013, Przybyłowicz et al. 2014) and chromosome numbers are known for a few Balkan populations (Coutsis and De Prins 2005, 2007, Kolev 2005).

The goal of the present study is a simultaneous investigation of chromosomal, molecular and morphological diversity in the anomalous blue butterflies from the Balkan Peninsula and interpretation of this diversity in terms of taxonomy. To achieve this goal, the following tasks were set:

To collect specimens of all the taxa of the complex described from the territory of the Balkan Peninsula. To collect specimens from different populations of these taxa.

To study their karyotypes (chromosome number and structure) using standard protocols for staining.

To obtain data on the variability of molecular markers: mitochondrial DNA barcode (*COI* gene fragment) and nuclear *internal transcribed spacer 2* (*ITS2*). These markers were selected because the usefulness of mitochondrial *COI* barcodes in taxonomic studies on species-level is generally recognized (Hebert et al. 2004, but see Wiemers and Fiedler 2007), and despite some limitations (Shapoval and Lukhtanov 2015c), *internal transcribed spacer 2* was found to be a useful nuclear marker in butterfly taxonomy (Wiemers et al. 2009).

To study the variability of the wing pattern characters which can be potentially useful for delimitation of species and populations (presence/reduction/absence of the white streak on the underside of the hindwings, the development of the marginal marking on the underside of the wings, presence or absence of a white stroke on the underside of the forewings).

To interpret the discovered chromosomal, molecular and morphological diversity in terms of taxonomy using two original methodologies: (1) detecting and taxonomic interpretation of cryptic entities found in sympatry and allopatry using combined analysis of mitochondrial and chromosomal markers (Lukhtanov et al. 2015a), and (2) critical evaluation of pre-existing morphology-based taxonomic hypotheses using DNA barcodes (Lukhtanov et al. 2016).

## Material and methods

### Taxon sampling

**Figure 1.**
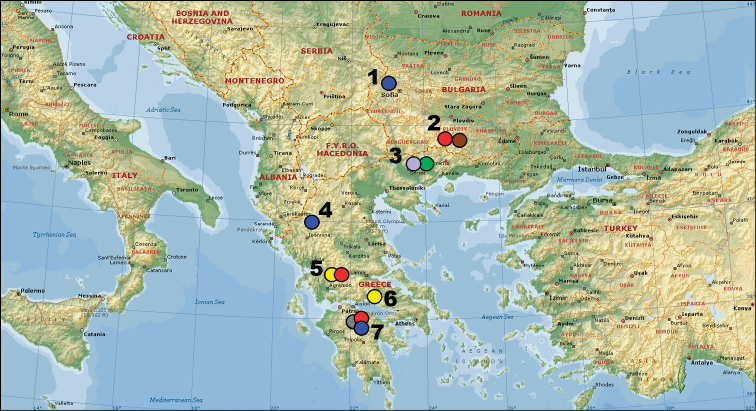
Localities of the species collected for the study (the species list is presented in Table 1). **1** Bulgaria: Dragoman (*Polyommatus admetus*) **2** Bulgaria: Hvoyna (*Polyommatus ripartii pelopi*, *Polyommatus orphicus orphicus*) **3** Greece: Granitis (*Polyommatus nephohiptamenos*, *Polyommatus orphicus eleniae*) **4** Greece: Smolikas (*Polyommatus admetus*) **5** Greece: Timfristos Mt (*Polyommatus ripartii pelopi*, *Polyommatus timfristos*) **6** Greece: Parnassos Mt (*Polyommatus timfristos*) **7** Greece: Kalavrita (*Polyommatus admetus*, *Polyommatus aroaniensis*, *Polyommatus ripartii pelopi*). Colored circles match different taxa. Blue circle: *Polyommatus admetus*. Red circle: *Polyommatus ripartii*. Brown circle: *Polyommatus orphicus orphicus*. Lavender circle: *Polyommatus orphicus eleniae*. Yellow circle: *Polyommatus timfristos* sp. n. Grey circle: *Polyommatus aroaniensis*.

Butterflies for this study were collected in 2008 in the Balkan Peninsula by V.A. Lukhtanov, N.A. Shapoval and L. Rieppel, in 2016 in Hvoyna village (Bulgaria) by E.A. Pazhenkova and in the Tigirekskiy Reservation (the Altai Mountains, Russia) by M.S.Vishnevskaya in 2007 (Fig. 1, Table 1). We paid special attention to collecting the taxa in their type localities: mount Chelmos (Greece: Peloponnese) (type locality of *Agrodiaetus alcestis aroaniensis* Brown, 1976), mount Falakró near Granítis (Greece, Makedonía, Dráma district) (type locality of *Agrodiaetus eleniae* Coutsis & De Prins, 2005) and Hvoyna (south Bulgaria, the Rhodopi mts) (type locality of *Polyommatus dantchenkoi orphicus*). Unfortunately, in our research we did not have an opportunity to study the holotypes of these taxa. Taking into account a possibility of multiple cryptic species within a local area even in well-studied European butterflies (Dincă et al. 2011, 2013b), in each place we managed to collect (and then to study) as many individuals as possible paying special attention to the specimens with unusual or intermediate morphology.

**Table 1.** List of butterflies collected for the present study*

Traditionally accepted name and combination	Proposed name and combination	Sample code	GenBank code COI	GenBank ITS2	Locality and date
*Polyommatus admetus*	*Polyommatus admetus*	08D109	KY050594		Greece, Kalavrita, 38°02.097'N; 22°07.085'E, 812 m, 17 July 2008
*Polyommatus admetus*	*Polyommatus admetus*	08D211	KY050595	KY066732	Greece, Kalavrita 38°02.097'N; 22°07.085'E, 1150 m, 19 July 2008
*Polyommatus admetus*	*Polyommatus admetus*	08D386	KY050596	KY066733	Greece, Smolikas, 40°03.175'N; 20°53.941'E, 1497 m, 22 July 2008
*Polyommatus admetus*	*Polyommatus admetus*	08D655	KY050597		Bulgaria, Dragoman, 42°56.320'N; 22°56.038'E, 753 m, 29 July 2008
*Polyommatus aroaniensis*	*Polyommatus aroaniensis*	08D102	KY050598	KY066734	Greece, Kalavrita, 38°00.699'N; 22°11.554'E, 1640, 16 July 2008
*Polyommatus aroaniensis*	*Polyommatus timfristos*	08D205	KY066724	KY081278	Greece, Parnassos, 38°33.311'N; 22°34.300'E, 1750m, 19 July 2008
*Polyommatus aroaniensis*	*Polyommatus timfristos*	08D247	KY066725	KY081279	Greece, Timfristos, 38°55.460'N; 21°47.605'E, 1267 m, 20 July 2008
*Polyommatus aroaniensis*	*Polyommatus timfristos*	08D255	KY066726	KY081280	Greece, Timfristos, 38°55.460'N; 21°47.605'E, 1267 m, 20 July 2008
*Polyommatus aroaniensis*	*Polyommatus timfristos*	08D258	KY066727	KY081281	Greece, Timfristos, 38°55.460'N; 21°47.605'E, 1267 m, 20 July 2008
*Polyommatus aroaniensis*	*Polyommatus timfristos*	08D273	KY066728	KY081282	Greece, Timfristos, 38°55.460'N; 21°47.605'E, 1267 m, 20 July 2008
*Polyommatus aroaniensis*	*Polyommatus timfristos*	08D274	KY066729	KY081283	Greece, Timfristos, 38°55.460'N; 21°47.605'E, 1267 m, 20 July 2008
*Polyommatus dantchenkoi orphicus*	*Polyommatus orphicus orphicus*	08D546	KY066698	KY081246	Bulgaria, Hvoyna, Rodopi Mts, 41°52'14"N; 24°41'6"E, 800 m, 26 July 2008
*Polyommatus dantchenkoi orphicus*	*Polyommatus orphicus orphicus*	08D560	KY066699	KY081247	Bulgaria, Hvoyna, Rodopi Mts, 41°52'14"N; 24°41'6"E, 800 m, 26 July 2008
*Polyommatus dantchenkoi orphicus*	*Polyommatus orphicus orphicus*	PE 002	KY066700	KY081266	Bulgaria, Hvoyna, Rodopi Mts, 41°52.14'N; 24°41.6'E, 950 m, 3–7 July 2016
*Polyommatus dantchenkoi orphicus*	*Polyommatus orphicus orphicus*	PE 003	KY066701	KY081267	Bulgaria, Hvoyna, Rodopi Mts, 41°52.14'N; 24°41.6'E, 950 m, 3–7 July 2016
*Polyommatus dantchenkoi orphicus*	*Polyommatus orphicus orphicus*	PE 006	KY066702	KY081268	Bulgaria, Hvoyna, Rodopi Mts, 41°52.14'N; 24°41.6'E, 950 m, 3–7 July 2016
*Polyommatus dantchenkoi orphicus*	*Polyommatus orphicus orphicus*	PE 010	KY066705	KY081271	Bulgaria, Hvoyna, Rodopi Mts, 41°52.14'N; 24°41.6'E, 950 m, 3–7 July 2016
*Polyommatus dantchenkoi orphicus*	*Polyommatus orphicus orphicus*	PE 011	KY066706	KY081272	Bulgaria, Hvoyna, Rodopi Mts, 41°52.14'N; 24°41.6'E, 950 m, 3–7 July 2016
*Polyommatus dantchenkoi orphicus*	*Polyommatus orphicus orphicus*	PE 012	KY066707	KY081273	Bulgaria, Hvoyna, Rodopi Mts, 41°52.14'N; 24°41.6'E, 950 m, 3–7 July 2016
*Polyommatus dantchenkoi orphicus*	*Polyommatus orphicus orphicus*	PE 013	KY066708	KY081274	Bulgaria, Hvoyna, Rodopi Mts, 41°52.14'N; 24°41.6'E, 950 m, 3–7 July 2016
*Polyommatus dantchenkoi orphicus*	*Polyommatus orphicus orphicus*	PE 014	KY066709	KY081275	Bulgaria, Hvoyna, Rodopi Mts, 41°52.14'N; 24°41.6'E, 950 m, 3–7 July 2016
*Polyommatus dantchenkoi orphicus*	*Polyommatus orphicus orphicus*	PE 015	KY066710	KY081276	Bulgaria, Hvoyna, Rodopi Mts, 41°52.14'N; 24°41.6'E, 950 m, 3–7 July 2016
*Polyommatus dantchenkoi orphicus*	*Polyommatus orphicus orphicus*	PE 016	KY066711	KY081277	Bulgaria, Hvoyna, Rodopi Mts, 41°52.14'N; 24°41.6'E, 950 m, 3–7 July 2016
*Polyommatus dantchenkoi orphicus*	*Polyommatus orphicus orphicus*	PE 007	KY066703	KY081269	Bulgaria, Hvoyna, Rodopi Mts, 41°52.14'N; 24°41.6'E, 950 m, 3–7 July 2016
*Polyommatus dantchenkoi orphicus*	*Polyommatus orphicus orphicus*	PE 008	KY066704	KY081270	Bulgaria, Hvoyna, Rodopi Mts, 41°52.14'N; 24°41.6'E, 950 m, 3–7 July 2016
*Polyommatus dantchenkoi orphicus*	*Polyommatus orphicus orphicus*	08D545	KY066697	KY081245	Bulgaria, Hvoyna, Rodopi Mts, 41°52.14'N; 24°41.6'E, 800 m, 26 July 2008
*Polyommatus eleniae*	*Polyommatus orphicus eleniae*	08D431	KY050599	KY066735	Greece, Granitis, 41°17.543'N; 23°56.265'E, 830 m, 23 July 2008
*Polyommatus eleniae*	*Polyommatus orphicus eleniae*	08D433	KY050600	KY066736	Greece, Granitis, 41°17.543'N; 23°56.265'E, 830 m, 23 July 2008
*Polyommatus eleniae*	*Polyommatus orphicus eleniae*	08D434	KY050601	KY081243	Greece, Granitis, 41°17.543'N; 23°56.265'E, 830 m, 23 July 2008
*Polyommatus eleniae*	*Polyommatus orphicus eleniae*	08D437	KY050602	KY081244	Greece, Granitis, 41°17.543'N; 23°56.265'E, 830 m, 23 July 2008
*Polyommatus nephohiptamenos*	*Polyommatus nephohiptamenos*	08D471	KY050603	KY081248	Greece, Granitis, 41°17.543'N; 23°56.265'E, 830 m, 23 July 2008
*Polyommatus nephohiptamenos*	*Polyommatus nephohiptamenos*	08D483	KY050604	KY081249	Greece, Granitis, 41°13.485'N; 24°02.990'E, 1646 m, 23 July 2008
*Polyommatus nephohiptamenos*	*Polyommatus nephohiptamenos*	08D485			Greece, Granitis, 41°13.485'N; 24°02.990'E 1646 m, 23 July 2008
*Polyommatus nephohiptamenos*	*Polyommatus nephohiptamenos*	08D494	KY050605	KY081250	Greece, Granitis, 41°13.485'N; 24°02.990'E, 1450–1750 m, 24 July 2008
*Polyommatus nephohiptamenos*	*Polyommatus nephohiptamenos*	08D496	KY050606	KY081251	Greece, Granitis, 41°13.485'N; 24°02.990'E, 1450–1750 m, 24 July 2008
*Polyommatus nephohiptamenos*	*Polyommatus nephohiptamenos*	08D498	KY066694	KY081252	Greece, Granitis, 41°13.485'N; 24°02.990'E, 1450–1750 m, 24 July 2008
*Polyommatus nephohiptamenos*	*Polyommatus nephohiptamenos*	08D499	KY066695	KY081253	Greece, Granitis, 41°13.485'N; 24°02.990'E, 1450–1750 m, 24 July 2008
*Polyommatus ripartii pelopi*	*Polyommatus ripartii pelopi*	08D249	KY066717	KY081258	Greece, Timfristos, 38°55.460'N; 21°47.605'E, 1267 m, 20 July 2008
*Polyommatus ripartii pelopi*	*Polyommatus ripartii pelopi*	08D252	KY066718	KY081259	Greece, Timfristos, 38°55.460'N; 21°47.605'E, 1267 m, 20 July 2008
*Polyommatus ripartii pelopi*	*Polyommatus ripartii pelopi*	08D257	KY066719	KY081260	Greece, Timfristos, 38°55.460'N; 21°47.605'E, 1267 m, 20 July 2008
*Polyommatus ripartii pelopi*	*Polyommatus ripartii pelopi*	08D260	KY066720	KY081263	Greece, Timfristos, 38°55.460'N; 21°47.605'E, 1267 m, 20 July 2008
*Polyommatus ripartii pelopi*	*Polyommatus ripartii pelopi*	08D291	KY066721	KY081261	Greece, Timfristos, 38°55.460'N; 21°47.605'E, 1267 m, 20 July 2008
*Polyommatus ripartii pelopi*	*Polyommatus ripartii pelopi*	08D549	KY066722	KY081262	Bulgaria, Hvoyna, Rodopi Mts, 41°52.14'N; 24°41.6'E, 800 m
*Polyommatus ripartii pelopi*	*Polyommatus ripartii pelopi*	08D551			Bulgaria, Hvoyna, Rodopi Mts, 41°52.14'N; 24°41.6'E, 800m, 26 July 2008
*Polyommatus ripartii pelopi*	*Polyommatus ripartii pelopi*	08D571	KY066723	KY081264	Bulgaria, Hvoyna, Rodopi Mts yna, 41°52.14'N; 24°41.6'E, 800 m, 26 July 2008
*Polyommatus ripartii pelopi*	*Polyommatus ripartii pelopi*	08D085	KY066712	KY081254	Greece, Kalavrita, 38°02.097'N; 22°07.085'E, 812 m, 16 July 2008
*Polyommatus ripartii pelopi*	*Polyommatus ripartii pelopi*	08D092	KY066713	KY081255	Greece, Kalavrita, 38°02.097'N; 22°07.085'E, 812 m, 16 July 2008
*Polyommatus ripartii pelopi*	*Polyommatus ripartii pelopi*	08D120	KY066714	KY081256	Greece Kalavrita, 38°02.097'N; 22°07.085'E, 812 m, 17 July 2008
*Polyommatus ripartii pelopi*	*Polyommatus ripartii pelopi*	08D144	KY066715	KY085933	Greece Kalavrita, 38°01.617'N; 22°13.411'E, 1610–1700 m, 17 July 2008
*Polyommatus ripartii pelopi*	*Polyommatus ripartii pelopi*	08D145	KY066716	KY081257	Greece, Kalavrita, 38°01.617'N; 22°13.411'E 1610–1700 m, 17 July 2008
*Polyommatus ripartii pelopi*	*Polyommatus ripartii pelopi*	PE 009	KY066696	KY081265	Bulgaria, Hvoyna, Rodopi Mts, 41°52.14'N 24°41.6'E, 950 m
*Plebejus damon*	*Plebejus damon*	VM237	KY066730		Russia, Altai Mts, Tigirek, 51°0'N; 82°55'E, 28 July 2007
*Plebejus damon*	*Plebejus damon*	VM196	KY066731		Russia, Altai Mts, Tigirek, 51°0'N; 82°55'E, 19 July 2007

* The samples 08D485 and 08D551 were not used for molecular analysis since the sequences obtained were too short.

Before processing butterflies were put in glassine envelopes and kept alive for less than one hour. Testes were removed and put into a vial with a fresh fixative (3:1, 96% ethanol: glacial acetic acid). The wings were removed and put into a glassine envelope, and the body was placed into a vial with 96% ethanol for further molecular analysis. All chromosome preparations, butterfly bodies in ethanol and wings in glassine envelopes are stored in the Department of Karyosystematics (Zoological Institute of the Russian Academy of Sciences, St. Petersburg).

### Analysis of karyotype

Testes were stored in the 3:1 fixative for several months at +4 °C and then stained with 2% acetic orcein for 30 days at 20 °C. We used a two-phase method of chromosome analysis following Lukhtanov and Dantchenko (2002b). In the first phase, stained testes were placed into a drop of 40% lactic acid on a slide where spermatocysts were dissected from testis membranes using entomological pins. Intact spermatocytes were transferred into a new drop of 40% lactic acid and covered with a coverslip. A Carl Zeiss Amplival light microscope was used for cytogenetic analysis. During the metaphase I stage, each spermatocyst was observed as a regular sphere consisting of 64 spermatocytes. In the second phase, different degrees of chromosome spreading were observed by gradually increasing pressure on the coverslip. The second phase was useful for studying the bivalent structure and counting the bivalent number. By scaling up the pressure on the coverslip, we were able to manipulate chromosomes, e.g. change their position and orientation on the slide, and consequently to resolve controversial cases of contacting or overlapping bivalents. Haploid chromosome numbers were counted in metaphase I (MI) and/or metaphase II (MII) of meiosis.

### DNA extraction and sequencing

We used a 657-bp fragment within the mitochondrial *COI* gene and a 440-bp fragment within the *ITS2* region. DNA was extracted using phenol-chloroform method according to the standard protocol (Sambrook and Russel 2006). The first two abdominal segments were homogenized in lysis buffer [25 mM EDTA, 75 mM NaCl, 10 mM Tris (pH 7.5)]. Then proteinase K (20 mg/ml) and 10% SDS were added and the samples were incubated for 2 h at 60 °C. DNA was extracted from lysate first with phenol/chloroform (1:1) and then with chloroform to remove any remaining phenol. DNA was precipitated with isopropyl alcohol in the presence of 0.1 M NaCl and pelleted by centrifugation. The pellets were washed with 70% ethanol, dried and dissolved in ddH2O. The extracted DNA was stored at -20 °C.

For *COI* amplification we used the self-designed primers *COI*F1 (5’-CCACAAATCATAAAGATATTGGAAC-3’) and *COI*R1 (5’-TGATGAGCTCATACAATAAATCCTA-3’). For *ITS2* amplification we used the self-designed primers *ITS2*F (5’-CATATGCCACACTGTTCGTCTG-3’) and *ITS2*R (5’-GATATCCGTCAGCGCAACG-3’).

The polymerase chain reaction (PCR) was carried out with Taq-polymerase (Sileks) in 20 µl of PCR buffer containing MgCl_2_ [2.5 mM], dNTP [200 mM] and forward and reverse primers [20 pmol each]. Amplification of *COI* gene fragment was carried out with the following conditions: initial denaturation at 94 °C for 3 min, followed by 30 cycles of 30 sec at 94 °C, 30 sec at 51 °C (the annealing temperature) and 30 sec at 72 °C, and then final elongation 5 min for 72 °C. Amplification of *ITS2* region fragment was carried out with the following conditions: initial denaturation at 94 °C for 2 min, followed by 30 cycles of 30 sec at 94 °C, 30 sec at 60 °C (the annealing temperature) and 30 sec at 72 °C, and then final elongation 5 min for 72 °C.

**Figure 2.**
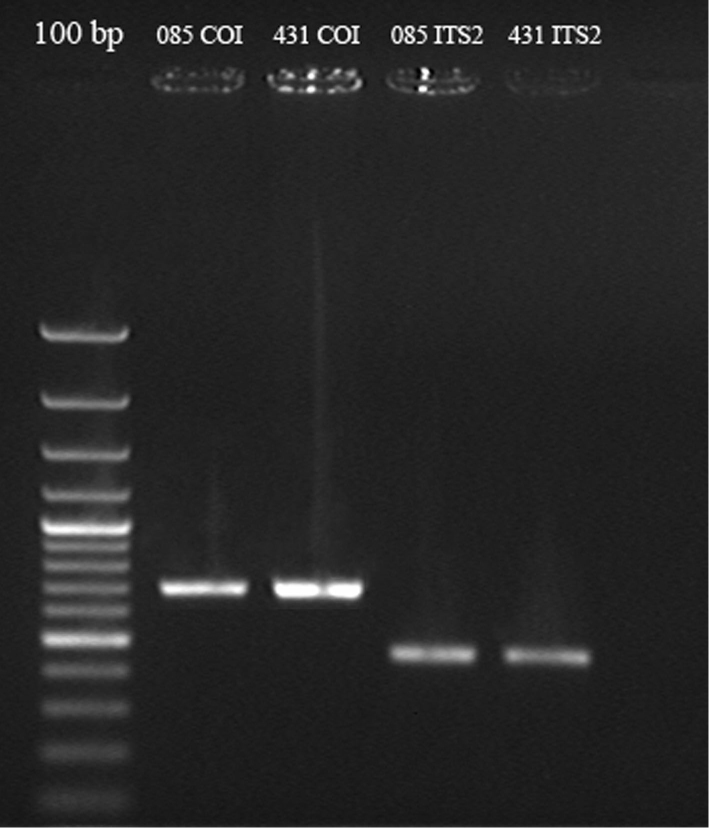
Gel electrophoresis with *COI* and *ITS2* PCR products showing the length of the fragments.

After amplification, PCR mix was loaded in 1% agarose gel and specific product was separated by gel electrophoresis (Fig. 2). Pieces of gel containing the DNA fragment of required length were cut out and then double-stranded DNA was purified using the method of ‘DNA purification from agarose gels with MP@SiO_2_ magnetic particles’ according to the manufacturer’s protocol (Sileks). Purified DNA fragments were extracted with ddH_2_O from magnetic particles pelleted with a magnetic rack and collected in a fresh tube. The concentration of purified DNA was estimated via gel electrophoresis (by comparing the brightness of the sample fragment to the brightness of the DNA marker (in our case 100 bp DNA Ladder, Thermo Fisher Scientific).

All the preparations for sequencing were held in “The Laboratory of Animal Genetics” of Saint-Petersburg State University and “Chromas” Core Facility, Saint-Petersburg State University Research Park. Sequencing was carried out in the Research Resource Center for Molecular and Cell Technologies. GenBank codes of the studied samples are provided in Tables 1 and 2.

**Table 2.** List of samples and haplogroups used for the present study.

Taxon and field code	*COI* GenBank code	*ITS2* GenBank code	*COI* haplogroup
*Polyommatus admetus* 08D109	KY050594		**ad_1**
*Polyommatus admetus* 08D211	KY050595	KY066732	**ad_2**
*Polyommatus admetus* 08D386	KY050596	KY066733	**ad_3**
*Polyommatus admetus*	AY556867	AY556733	**ad_4**
*Polyommatus admetus*	AY556986		**ad_5**
*Polyommatus admetus*	KC581753		**ad_6**
*Polyommatus admetus*	KC581754		**ad_7**
*Polyommatus alcestis alcestis*	AY557008	AY556641	**alc_3**
*Polyommatus aroaniensis* 08D102	KY050598	KY066734	**ar_1**
*Polyommatus aroaniensis*	AY556856	AY556725	**ar_2**
*Polyommatus dantchenkoi*	AY557072	AY556678	**dan_1**
*Polyommatus dantchenkoi*	AY557081	AY556685	
*Polyommatus dantchenkoi*	AY557073	AY556679	
*Polyommatus demavendi belovi*	KR265493		**dem_1**
*Polyommatus demavendi belovi*	KR265494		**dem_2**
*Polyommatus demavendi belovi*	EF104630		**dem_3**
*Polyommatus demavendi lorestanus*	AY557142	AY556743	**dem_4**
*Polyommatus dolus virgilius*	HM210162	HM210180	**dol_1**
*Polyommatus dolus vittatus*	AY496740		**dol_2**
*Polyommatus fabressei*	AY496744		**fab_1**
*Polyommatus fabressei*	AY556952	AY556608	
*Polyommatus fabressei*	AY556869	AY556734	**fab_1**
*Polyommatus fulgens*	AY556941	AY556601	
*Polyommatus fabressei*	EF104605	HM210186	**fab_4**
*Polyommatus fulgens*	AY556963	AY556615	**ful_1**
*Polyommatus fulgens*	AY496746		
*Polyommatus fulgens*	AY496712		
*Polyommatus fulgens*	AY556954	AY556610	**ful_2**
*Polyommatus fulgens*	AY556958		**ful_4**
*Polyommatus humedasae*	AY557127	AY556710	**hum_1**
*Polyommatus humedasae*	AY557128	AY556711	**hum_2**
*Polyommatus humedasae*	HM210169	HM210192	
*Polyommatus humedasae*	HM210170	HM210193	**hum_4**
*Polyommatus karacetinae*	AY556906		**alc_1**
*Polyommatus karacetinae*	AY556907	AY556574	**alc_1**
*Polyommatus karacetinae*	AY557090		**alc_4**
*Polyommatus karacetinae urmiaensis*	EF104631		**urm**
*Polyommatus khorasanensis*	AY557138	AY556737	**khor**
*Polyommatus menalcas*	AY556982		**men_1**
*Polyommatus menalcas*	AY557111		**men_2**
*Polyommatus menalcas*	AY557001	AY556635	**men_3**
*Polyommatus nephohiptamenos* 08D471	KY050603	KY081248	**ne_1**
*Polyommatus nephohiptamenos* 08D483	KY050604	KY081249	
*Polyommatus nephohiptamenos* 08D499	KY066695	KY081253	
*Polyommatus nephohiptamenos* 08D496	KY050606	KY081251	
*Polyommatus nephohiptamenos* 08D494	KY050605	KY081250	**ne_3**
*Polyommatus nephohiptamenos* 08D498	KY066694	KY081252	**ne_5**
*Polyommatus nephohiptamenos*	KC581745		**ne_7**
*Polyommatus nephohiptamenos*	AY556860		
*Polyommatus nephohiptamenos*	AY556859	AY556728	
*Polyommatus orphicus eleniae* 08D431	KY050599	KY066735	**orph_1**
*Polyommatus orphicus eleniae* 08D433	KY050600	KY066736	
*Polyommatus orphicus eleniae* 08D437	KY050602	KY081244	
*Polyommatus orphicus eleniae* 08D434	KY050601	KY081243	**orph_3**
*Polyommatus orphicus orphicus* 08D545	KY066697	KY081245	**orph_5**
*Polyommatus orphicus orphicus* 08D560	KY066699	KY081247	
*Polyommatus orphicus orphicus* PE 003	KY066701	KY081267	**orph_5**
*Polyommatus orphicus orphicus* PE 011	KY066706	KY081272	
*Polyommatus orphicus orphicus* PE 013	KY066708	KY081274	
*Polyommatus orphicus orphicus* PE 014	KY066709	KY081275	
*Polyommatus orphicus orphicus* PE 015	KY066710	KY081276	
*Polyommatus orphicus orphicus* PE 007	KY066703	KY081269	
*Polyommatus orphicus orphicus* PE 006	KY066702	KY081268	
*Polyommatus orphicus orphicus* PE 012	KY066707	KY081273	**orph_6**
*Polyommatus orphicus orphicus* PE 008	KY066704	KY081270	
*Polyommatus orphicus orphicus* 08D546	KY066698	KY081246	
*Polyommatus orphicus orphicus* PE 002	KY066700	KY081266	**orph_8**
*Polyommatus orphicus orphicus* PE 010	KY066705	KY081271	**orph_11**
*Polyommatus orphicus orphicus* PE 016	KY066711	KY081277	
*Polyommatus pseudorjabovi*	KR265487		**pse_1**
*Polyommatus pseudorjabovi*	KR265489		
*Polyommatus pseudorjabovi*	KR265490		
*Polyommatus pseudorjabovi*	KR265491		**pse_1**
*Polyommatus pseudorjabovi*	KR265484		
*Polyommatus pseudorjabovi*	KR265480		
*Polyommatus pseudorjabovi*	KR265496		**pse_2**
*Polyommatus pseudorjabovi*	KR265483		
*Polyommatus pseudorjabovi*	KR265481		
*Polyommatus pseudorjabovi*	KR265488		**pse_3**
*Polyommatus pseudorjabovi*	KR265482		**pse_9**
*Polyommatus pseudorjabovi*	KR265500		**pse_12**
*Polyommatus ripartii pelopi* 08D249	KY066717	KY081258	**rip_1**
*Polyommatus ripartii pelopi* 08D252	KY066718	KY081259	
*Polyommatus ripartii pelopi* 08D257	KY066719	KY081260	**rip_3**
*Polyommatus ripartii pelopi* 08D260	KY066720	KY081263	**rip_4**
*Polyommatus ripartii pelopi* 08D291	KY066721	KY081261	
*Polyommatus ripartii pelopi* 08D549	KY066722	KY081262	
*Polyommatus ripartii pelopi* 08D085	KY066712	KY081254	
*Polyommatus ripartii pelopi* 08D145	KY066716	KY081257	
*Polyommatus ripartii ripartii*	AY556858	AY556727	
*Polyommatus ripartii ripartii*	KC581746		
*Polyommatus ripartii ripartii*	KC581747		
*Polyommatus ripartii ripartii*	KC581748		
*Polyommatus ripartii ripartii*	KC581749		
*Polyommatus ripartii ripartii*	KC581750		
*Polyommatus ripartii ripartii*	KC581751		
*Polyommatus ripartii ripartii*	KC581752		
*Polyommatus ripartii pelopi* 08D571	KY066723	KY081264	
*Polyommatus ripartii paralcestis*	KC581715		**rip_8**
*Polyommatus ripartii paralcestis*	KC581716		**rip_9**
*Polyommatus ripartii pelopi*	AY557042		**rip_10**
*Polyommatus ripartii pelopi* 08D092	KY066713	KY081255	**rip_12**
*Polyommatus ripartii pelopi* 08D120	KY066714	KY081256	**rip_13**
*Polyommatus ripartii pelopi* 08D144	KY066715	KY085933	**rip_14**
*Polyommatus ripartii pelopi* PE 009	KY066696	KY081265	**rip_82**
*Polyommatus ripartii ripartii*	HM210164		**rip_16**
*Polyommatus ripartii ripartii*	HM210172		
*Polyommatus ripartii riparii*	HM210163	HM210197	
*Polyommatus ripartii ripartii*	AY556944	AY556603	**rip_18**
*Polyommatus ripartii ripartii*	KC581717		**rip_19**
*Polyommatus ripartii ripartii*	KC581718		
*Polyommatus ripartii ripartii*	AY556957		
*Polyommatus ripartii ripartii*	AY556962		**rip_20**
*Polyommatus ripartii ripartii*	EF104603		**rip_21**
*Polyommatus ripartii ripartii*	FJ663243		**rip_22**
*Polyommatus ripartii ripartii*	FJ663244		**rip_23**
*Polyommatus ripartii ripartii*	FJ663245		
*Polyommatus ripartii ripartii*	FJ663246		
*Polyommatus ripartii ripartii*	JN276883		**rip_26**
*Polyommatus ripartii ripartii*	GU675760		
*Polyommatus ripartii ripartii*	GU676039		**rip_27**
*Polyommatus ripartii ripartii*	GU676152		
*Polyommatus ripartii ripartii*	GU677012		
*Polyommatus ripartii ripartii*	GU677029		
*Polyommatus ripartii ripartii*	HM901559		
*Polyommatus ripartii ripartii*	HM901664		
*Polyommatus ripartii ripartii*	KC581736		
*Polyommatus ripartii ripartii*	KC581737		
*Polyommatus ripartii ripartii*	KC581738		
*Polyommatus ripartii ripartii*	KC581739		
*Polyommatus ripartii ripartii*	KC581740		
*Polyommatus ripartii ripartii*	GU676158		
*Polyommatus ripartii ripartii*	GU676213		**rip_30**
*Polyommatus ripartii ripartii*	KC617793		**rip_31**
*Polyommatus ripartii ripartii*	KC617794		
*Polyommatus ripartii ripartii*	GU676220		**rip_31**
*Polyommatus ripartii ripartii*	HM210167		**rip_35**
*Polyommatus ripartii ripartii*	KC581741		**rip_36**
*Polyommatus ripartii ripartii*	KC581742		
*Polyommatus ripartii ripartii*	KC581743		
*Polyommatus ripartii ripartii*	HM210168		
*Polyommatus ripartii ripartii*	KC581723		**rip_37**
*Polyommatus ripartii ripartii*	KC581724		
*Polyommatus ripartii ripartii*	KC581725		
*Polyommatus ripartii ripartii*	HM210171		
*Polyommatus ripartii ripartii*	KC567885		**rip_42**
*Polyommatus ripartii ripartii*	KC581719		
*Polyommatus ripartii ripartii*	KC567883		
*Polyommatus ripartii ripartii*	KC567884		**rip_43**
*Polyommatus ripartii ripartii*	KC581720		**rip_48**
*Polyommatus ripartii ripartii*	KC581721		**rip_49**
*Polyommatus ripartii ripartii*	KC581722		**rip_50**
*Polyommatus ripartii ripartii*	KC581726		**rip_54**
*Polyommatus ripartii ripartii*	KC581727		**rip_55**
*Polyommatus ripartii ripartii*	KC581728		
*Polyommatus ripartii ripartii*	KC581729		**rip_57**
*Polyommatus ripartii ripartii*	KC581730		
*Polyommatus ripartii ripartii*	KC581731		
*Polyommatus ripartii ripartii*	KC581732		
*Polyommatus ripartii ripartii*	KC581733		
*Polyommatus ripartii ripartii*	KC581734		**rip_62**
*Polyommatus ripartii ripartii*	KC581735		
*Polyommatus ripartii ripartii*	KC581744		**rip_72**
*Polyommatus rjabovianus rjabovianus*	KR265475		**rja_1**
*Polyommatus rjabovianus rjabovianus*	KR265476		
*Polyommatus rjabovianus rjabovianus* 2014A10			
*Polyommatus rjabovianus rjabovianus*	KR265477		
*Polyommatus rjabovianus masul*	KR265497		**rja_4**
*Polyommatus rjabovianus masul*	KR265485		
*Polyommatus rjabovianus masul*	KR265498		
*Polyommatus rjabovianus masul*	AY954006		
*Polyommatus rjabovianus masul*	KR265499		
*Polyommatus rjabovianus rjabovianus*	KR265478		**rja_5**
*Polyommatus rjabovianus rjabovianus*	AY954019		
*Polyommatus timfristos* 08D205	KY066724	KY081278	**tim_1**
*Polyommatus timfristos* 08D247 Holotype	KY066725	KY081279	**tim_2**
*Polyommatus timfristos* 08D273	KY066728	KY081282	
*Polyommatus timfristos* 08D274	KY066729	KY081283	
*Polyommatus timfristos* 08D255	KY066726	KY081280	
*Polyommatus timfristos* 08D258	KY066727	KY081281	**tim_4**
*Polyommatus valiabadi*	KR265495		**val_1**
*Polyommatus valiabadi*	KR265486		
*Polyommatus valiabadi*	AY556934	AY556594	
*Polyommatus valiabadi*	AY556882	AY556557	
*Polyommatus violetae subbaeticus*	EF104604	HM210188	**viol_1**
*Polyommatus violetae subbaeticus*	HM210166	HM210187	**viol_2**
*Polyommatus violetae violetae*	HM210173	HM210200	**viol_3**
*Polyommatus violetae violetae*	HM210174	HM210201	
*Polyommatus violetae violetae*	HM210175	HM210202	**viol_5**
*Polyommatus yeranyani malyevi*	KJ906515		**ad_8**
*Polyommatus yeranyani yeranyani*	KR265492		**ad_9**

### Phylogenetic analysis

The analysis involved 221 *COI* sequences (169 GenBank sequences and 52 own material) and 117 *ITS2* sequences (66 GenBank and 51 own data).

Sequences of different length (from 415 to 657 bp in case of *COI* and from 415 to 440 bp in case of *ITS2*) were included into the final dataset alignment. We used BioEdit 7.2.5 software (Hall 1999) to align the sequences and then edited them manually. The final *COI* alignment included 657 sites, with 137 variable sites and 112 parsimony-informative sites. The final *ITS2* alignment included 440 sites, with 52 variable sites and 22 parsimony-informative sites.

Previously, no significant conflict was detected between the mitochondrial *COI* and nuclear *ITS2* *Agrodiaetus* data sets (Vila et al. 2010). Thus, we combined mitochondrial and nuclear sequences to improve phylogenetic signal. This resulted in a concatenated alignment with a total of 1039 bp.

Phylogenetic relationships were inferred using Bayesian Inference (BI), maximum likelihood (ML) and maximum parsimony (MP) analyses. jModelTest was used to determine optimal substitution models for ML inference (Posada 2008).

Bayesian analyses were conducted using MrBayes, version 3.2 (Ronquist et al. 2012). Datasets were partitioned by codon position. Substitution models used for each partition were chosen according to jModelTest (Posada 2008): nst=2 and rates=invgamma for the first position, nst=2 and rates=gamma for the second position, and nst=6 and rates=gamma for the third position of *COI* barcodes. Substitution model nst=6 and rates=invgamm was chosen for *ITS2*. In evolution of *ITS2* sequences, the mono, bi- and mullti-nucleotide insertions/deletions are frequent and contain phylogenetically important information. To account for this, each indel event was coded as a binary character (1/0, presence/absence of the gap independently of its length) and then used in the Bayesian analyses of *ITS2* and concatenated data sets. Two runs of 10 000 000 generations with four chains (one cold and three heated) were performed. Chains were sampled every 10 000 generations, and burn-in was determined based on inspection of log likelihood over time plots using TRACER, version 1.4 (available from http://beast.bio.ed.ac.uk/Tracer).

The ML trees were inferred using MEGA6 under the GTR+G+I model. MP analysis was performed using a heuristic search as implemented in MEGA6 (Tamura et al. 2013). A heuristic search was carried out using the close-neighbor-interchange algorithm with search level 3 (Nei and Kumar 2000) in which the initial trees were obtained with the random addition of sequences (100 replicates). We used nonparametric bootstrap values (Felsenstein 1985) to estimate branch support for ML and MP trees. The bootstrap consensus tree was inferred from 500 replicates.

### Haplotype network

Median network was constructed using the program Network 4.6.1.3. (Fluxus Technology, fluxus-engineering.com), with the Median Joining algorithm (Bandelt 1999). The algorithm picks close haplotype groups and finds hypothetical ancestors, to join the haplotypes in a common parsimony network. The program shows each haplotype with a colored circle. When the haplotypes are identical, they are united in one bigger circle under one name. Similar haplotypes then are combined in haplogroups (Table 2). The network was constructed on the base of *COI* alignment, with 191 sequences. The length of the sequences was 612 bp with 116 parsimony-informative sites. The final alignment included only sequences of equal length. Short and ambiguous sequences were excluded.

## Karyotypes of the studied samples

Table 3

**Table 3.** Chromosome numbers of the studied samples.

Code	Species	Chomosome number	Country	Locality	Elevation	Date
LR08D109	*Polyommatus admetus*	n=80	Greece (South)	Mt. Chelmos (Aroania), Kalavrita, 38°02.097'N; 22°07.085'E	812m	2008.VII.17
LR08D211	*Polyommatus admetus*	n=80	Greece (Central)	Timfristos Mt, Karpenisi, 38°55.460'N; 21°47.605'E	1267m	2008.VII.20
LR08D386	*Polyommatus admetus*	n=caca80	Greece	Smolikas Mt, Pades, 40°03.175'N; 20°53.941'E	1497m	2008.VII.22
LR08D655	*Polyommatus admetus*	n=ca80	Bulgaria	Dragoman, 42°56.320'N; 22°56.038'E	753m	2008.VII.29
LR08D085	*Polyommatus ripartii pelopi*	2n=ca180	Greece (South)	Mt. Chelmos (Aroania), Kalavrita, 38°02.097'N; 22°07.085'E	812m	2008.VII.16
LR08D092	*Polyommatus ripartii pelopi*	n=90	Greece (South)	Mt. Chelmos (Aroania), Kalavrita, 38°02.097'N; 22°07.085'E	812m	2008.VII.16
LR08D120	*Polyommatus ripartii pelopi*	2n=ca180	Greece (South)	Mt. Chelmos (Aroania), Kalavrita, 38°02.097'N; 22°07.085'E	812m	2008.VII.17
LR08D144	*Polyommatus ripartii pelopi*	n=90	Greece (South)	Mt. Chelmos (Aroania), Kalavrita, 38°01.617'N; 22°13.411'E	1610–1700m	2008.VII.17
LR08D145	*Polyommatus ripartii pelopi*	n=90	Greece (South)	Mt. Chelmos (Aroania), Kalavrita, 38°01.617'N; 22°13.411'E	1610–1700m	2008.VII.17
LR08D249	*Polyommatus ripartii pelopi*	n=90	Greece (Central)	Timfristos Mt, Karpenisi, 38°55.460'N; 21°47.605'E	1267m	2008.VII.20
LR08D252	*Polyommatus ripartii pelopi*	n=ca90	Greece (Central)	Timfristos Mt, Karpenisi, 38°55.460'N; 21°47.605'E	1267m	2008.VII.20
LR08D257	*Polyommatus ripartii pelopi*	n=90	Greece (Central)	Timfristos Mt, Karpenisi, 38°55.460'N; 21°47.605'E	1267m	2008.VII.20
LR08D260	*Polyommatus ripartii pelopi*	2n=ca180	Greece (Central)	Timfristos Mt, Karpenisi, 38°55.460'N; 21°47.605'E	1267m	2008.VII.20
LR08D291	*Polyommatus ripartii pelopi*	n=ca90	Greece (Central)	Timfristos Mt, Karpenisi, 38°55.460'N; 21°47.605'E	1267m	2008.VII.20
LR08D549	*Polyommatus ripartii pelopi*	n=ca90	Bulgaria	Rodopi Mts, Hvoyna, 41°15'N; 24°32'E	800m	2008.VII.26
LR08D571	*Polyommatus ripartii pelopi*	n=90	Bulgaria	Rodopi Mts, Hvoyna, 41°15'N; 24°32'E	800m	2008.VII.26
LR08D562	*Polyommatus ripartii pelopi*	n=90	Bulgaria	Rodopi Mts, Hvoyna, 41°15'N; 24°32'E	800m	2008.VII.26
LR08D471	*Polyommatus nephohiptamenos*	n=90	Greece (North)	Granitis, 41°17.543'N; 23°56.265'E	830m	2008.VII.23
LR08D483	*Polyommatus nephohiptamenos*	n=ca90	Greece (Northern)	Falakro Mt, 41°13.485'N; 24°02.990'E	1646m	2008.VII.23
LR08D485	*Polyommatus nephohiptamenos*	n=ca90	Greece (North)	Falakro Mt, 41°13.485'N; 24°02.990'E	1646m	2008.VII.23
LR08D494	*Polyommatus nephohiptamenos*	n=90	Greece (North)	Falakro Mt, 41°13.485'N; 24°02.990'E	1450–1750m	2008.VII.24
LR08D496	*Polyommatus nephohiptamenos*	n=ca90	Greece (North)	Falakro Mt, 41°13.485'N; 24°02.990'E	1450–1750m	2008.VII.24
LR08D498	*Polyommatus nephohiptamenos*	n=ca90	Greece (North)	Falakro Mt, 41°13.485'N; 24°02.990'E	1450–1750m	2008.VII.24
LR08D102	*Polyommatus aroaniensis*	n=47	Greece (South)	Mt. Chelmos (Aroania), Kalavrita, 38°00.699'N; 22°11.554'E	1640m	2008.VII.16
LR08D247 Holotype	*Polyommatus timfristos*	n=38	Greece (Central)	Timfristos Mt, Karpenisi, 38°55.460'N; 21°47.605'E	1267m	2008.VII.20
LR08D255	*Polyommatus timfristos*	n=38	Greece (Central)	Timfristos Mt, Karpenisi, 38°55.460'N; 21°47.605'E	1267m	2008.VII.20
LR08D258	*Polyommatus timfristos*	n=38	Greece (Central)	Timfristos Mt, Karpenisi, 38°55.460'N; 21°47.605'E	1267m	2008.VII.20
LR08D273	*Polyommatus timfristos*	n=38	Greece (Central)	Timfristos Mt, Karpenisi, 38°55.460'N; 21°47.605'E	1267m	2008.VII.20
LR08D274	*Polyommatus timfristos*	n=38	Greece (Central)	Timfristos Mt, Karpenisi, 38°55.460'N; 21°47.605'E	1267m	2008.VII.20
LR08D205	*Polyommatus timfristos*	n=38	Greece (Central)	Parnassos Mt, 38°33.311'N; 22°34.300'E	1750m	2008.VII.19
LR08D545	*Polyommatus orphicus orphicus*	n=ca41–42	Bulgaria	Rodopi Mts, Hvoyna, 41°15'N; 24°32'E	800m	2008.VII.26
LR08D546	*Polyommatus orphicus orphicus*	n=ca41–42	Bulgaria	Rodopi Mts, Hvoyna, 41°15'N; 24°32'E	800m	2008.VII.26
LR08D560	*Polyommatus orphicus orphicus*	n=41, n=42	Bulgaria	Rodopi Mts, Hvoyna, 41°15'N; 24°32'E	800m	2008.VII.26
LR08D561	*Polyommatus orphicus orphicus*	n=41, n=42	Bulgaria	Rodopi Mts, Hvoyna, 41°15'N; 24°32'E	800m	2008.VII.26
LR08D431	*Polyommatus orphicus eleniae*	n=42	Greece (North)	Granitis, 41°17.543'N; 23°56.265'E	830m	2008.VII.23
LR08D433	*Polyommatus orphicus eleniae*	n=41, n=42	Greece (North)	Granitis, 41°17.543'N; 23°56.265'E	830m	2008.VII.23
LR08D434	*Polyommatus orphicus eleniae*	n=ca42	Greece (North)	Granitis, 41°17.543'N; 23°56.265'E	830m	2008.VII.23
LR08D437	*Polyommatus orphicus eleniae*	n=ca42	Greece (North)	Granitis, 41°17.543'N; 23°56.265'E	830m	2008.VII.23

### *Polyommatus admetus*

Fig. 3a–c

The haploid chromosome number n=80 was found in MI and MII cells of two studied individuals from South and Central Greece. In two specimens (Greece, Smolikas Mt and Bulgaria) we counted approximately n=ca80 at MI. The last count was performed with an approximation due to the overlapping of some bivalents. The karyotype displayed one larger bivalent in the centre of the MI plate and one larger univalent in the centre of the MII plate.

**Figure 3.**
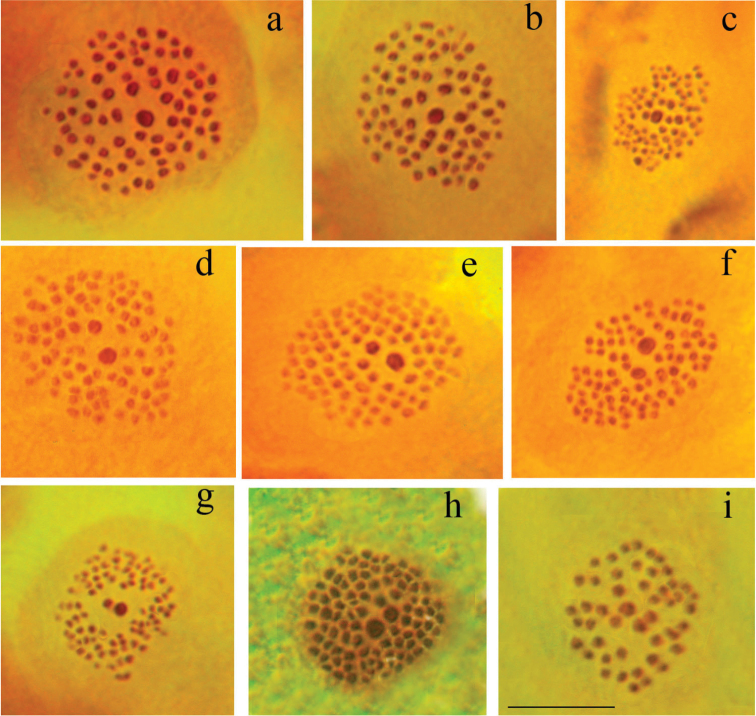
Polyommatus (Agrodiaetus) karyotypes. Bar =10 µ. **a–b** *Polyommatus admetus*, sample LR08D109, Greece, MI, n=80. One large bivalent in the centre of the plate can be seen **c** *Polyommatus admetus*, sample LR08D109, Greece, MII, n=80. One large chromosome in the centre of the plate can be seen **d** *Polyommatus ripartii pelopi*, sample LR08D249, Greece, MI, n=90. Two large bivalents in the centre of the plate can be seen **e** *Polyommatus ripartii pelopi*, sample LR08D144, Greece, MI, n=90. Two large bivalents in the centre of the plate can be seen **f** *Polyommatus ripartii pelopi*, sample LR08D145, Greece, MI, n=90. Two large bivalents in the centre of the plate can be seen **g** *Polyommatus ripartii pelopi*, sample LR08D92, Greece, MII, n=90. Two large chromosomes in the centre of the plate can be seen **h** *Polyommatus nephohiptamenos*, sample LR08D494, Northern Greece, MI, n=90. All the bivalents are situated in a plane with the largest elements in the centre of the circular metaphase plate. Bivalents are clearly separated from each other by gaps. Two bivalents are larger than the rest. **i** *Polyommatus aroaniensis*, sample LR08D102, Greece, MI, n=47.

### *Polyommatus ripartii pelopi*

Fig. 3d–g

The haploid chromosome number was determined to be n=90 in MI and MII cells of seven studied individuals from different localities (Greece, Bulgaria). At MI, two bivalents were especially large and were situated in the centre of the metaphase plates. Bivalent 1 was 1.4–1.6 times larger than bivalent 2. The sizes of the remaining 88 bivalents decreased more or less linearly. At MII, two univalents were especially large and were situated in the centre of the metaphase plates. Chromosome 1 was 1.4–1.6 times larger than chromosome 2. The sizes of the remaining 88 chromosomes decreased more or less linearly. In three specimens we counted approximately n=ca 90 at MI. The last count was an approximation due to the overlapping of some bivalents. In three specimens, the diploid chromosome number was estimated as 2n=ca180 in male asynaptic meiosis.

### *Polyommatus nephohiptamenos*

Fig. 3h

The haploid chromosome number was determined to be n=90 in MI and MII cells of two studied individuals. At MI, two bivalents (one big and one medium-sized) were larger than the others. At MII, two univalents (one big and one medium-sized) were larger than the rest. The sizes of the remaining 88 bivalents and univalents decreased more or less linearly. In four specimens we counted approximately n=ca90 at MI. The last count was an approximation due to the overlapping of some bivalents.

### *Polyommatus aroaniensis*

Fig. 3i

In the single studied specimen collected in the type locality (Greece, Mt. Chelmos) haploid chromosome number n=47 was found in MI cells. Bivalents were fairly well differentiated with respect to their size. However, it was difficult to subdivide them objectively into size groups because the sizes of the 47 bivalents decrease more or less linearly.

### *Polyommatus timfristos* Lukhtanov, Vishnevskaya & Shapoval, sp. n.

Figs 4a–h, 5a–d

The haploid chromosome number was determined to be n=38 in prometaphase, MI and MII cells of the holotype and six studied paratypes. Bivalents at MI and prometaphase and univalents at MII were fairly well differentiated with respect to their size; however, it was difficult to subdivide them objectively into size groups because the sizes of the 47 elements decrease more or less linearly.

**Figure 4.**
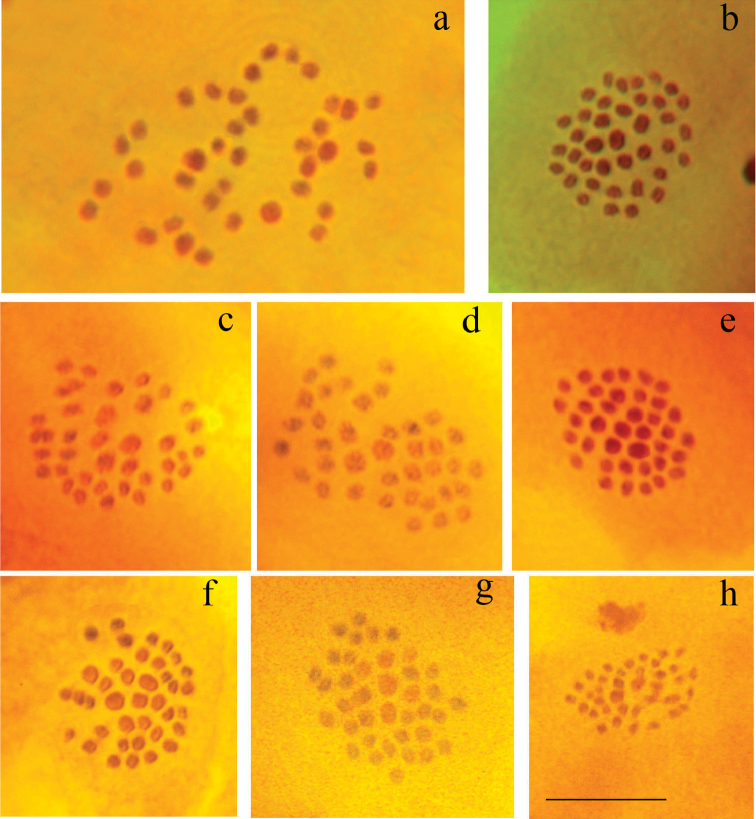
Polyommatus (Agrodiaetus) timfristos karyotypes. Bar = 10 µ. **a** *Polyommatus timfristos*, sample LR08D205, Central Greece, Parnassos, first prometaphase of meiosis, n=38 **b** *Polyommatus timfristos*, sample LR08D205, Central Greece, Parnassos, MI, n=38 **c** *Polyommatus timfristos*, holotype, sample LR08D247, Central Greece, Timfristos, MI, n=38 **d** *Polyommatus timfristos*, sample LR08D255, Central Greece, Timfristos, MI, n=38 **e** *Polyommatus timfristos*, sample LR08D258, Central Greece, Timfristos, MI, n=38 **f** *Polyommatus timfristos*, sample LR08D258, Central Greece, Timfristos, MI, n=38 **g** *Polyommatus timfristos*, sample LR08D273, Central Greece, Timfristos, MI, n=38 **h** *Polyommatus timfristos*, sample LR08D274, Central Greece, Timfristos, MII, n=38.

**Figure 5.**
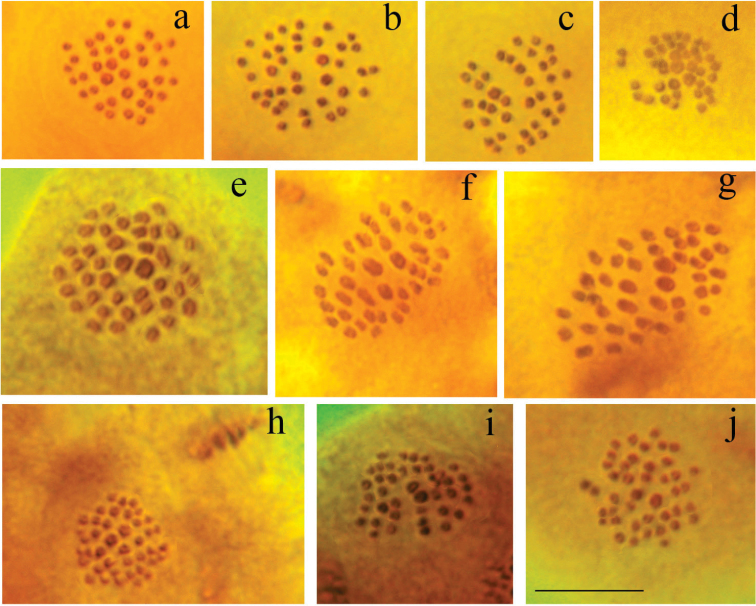
Polyommatus (Agrodiaetus) karyotypes. Bar = 10 µ. **a** *Polyommatus timfristos*, sample LR08D205, Central Greece, Parnassos, MII, n=38 **b** *Polyommatus timfristos*, sample LR08D205, Central Greece, Parnassos, MII, n=38 **c** *Polyommatus timfristos*, sample LR08D205, Central Greece, Parnassos, MII, n=38 **d** *Polyommatus timfristos*, sample LR08D258, Central Greece, Timfristos, MII, n=38 **e** *Polyommatus orphicus eleniae*, sample LR08D433, Northern Greece, MI, n=41 **f** *Polyommatus orphicus eleniae*, sample LR08D431, Northern Greece, MI, n=42 **g** *Polyommatus orphicus eleniae*, sample LR08D431, Northern Greece, MI, n=ca42 **h** *Polyommatus orphicus eleniae*, sample LR08D437, Northern Greece, MII, n=41 **i** *Polyommatus orphicus eleniae*, sample LR08D437, Northern Greece, MII, n=41 **j** *Polyommatus orphicus eleniae*, sample LR08D431, Northern Greece, MII, n=42.

### *Polyommatus orphicus orphicus*

Two different haploid chromosome numbers (n=41 and n=42) were observed in MI and MII cells of the four specimens studied. This variation was most likely caused by polymorphism for one chromosome fussion/fission. This polymorphism resulted in three types of MI karyotype: n=41 (homozygous for chromosomal fusion/fission, one pair of fused chromosomes), n=42 (homozygous for chromosomal fusion/fission, two pairs of unfused chromosomes) and n=41 (heterozygous for chromosomal fusion/fission, 40 bivalents and one trivalent). Bivalents at MI and univalents at MII were fairly well differentiated with respect to their size; however, it was difficult to subdivide them objectively into size groups because the sizes of the elements decrease more or less linearly.

### *Polyommatus orphicus eleniae*

Fig. 5e–j

Chromosome numbers (n=41 and n=42) were observed in MI and MII cells of the four specimens studied. This variation was most likely caused by polymorphism for one chromosome fussion/fission. This polymorphism resulted in three types of MI karyotype: n=41 (homozygous for chromosomal fusion/fission, one pair of fused chromosomes), n=42 (homozygous for chromosomal fusion/fission, two pairs of unfused chromosomes) and n=41 (heterozygous for chromosomal fusion/fission, 40 bivalents and one trivalent). Bivalents and univalents were fairly well differentiated with respect to their size; however, it was difficult to subdivide them objectively into size groups because the sizes of the elements decrease more or less linearly.

## Phylogenetic reconstruction

**Figure 6.**
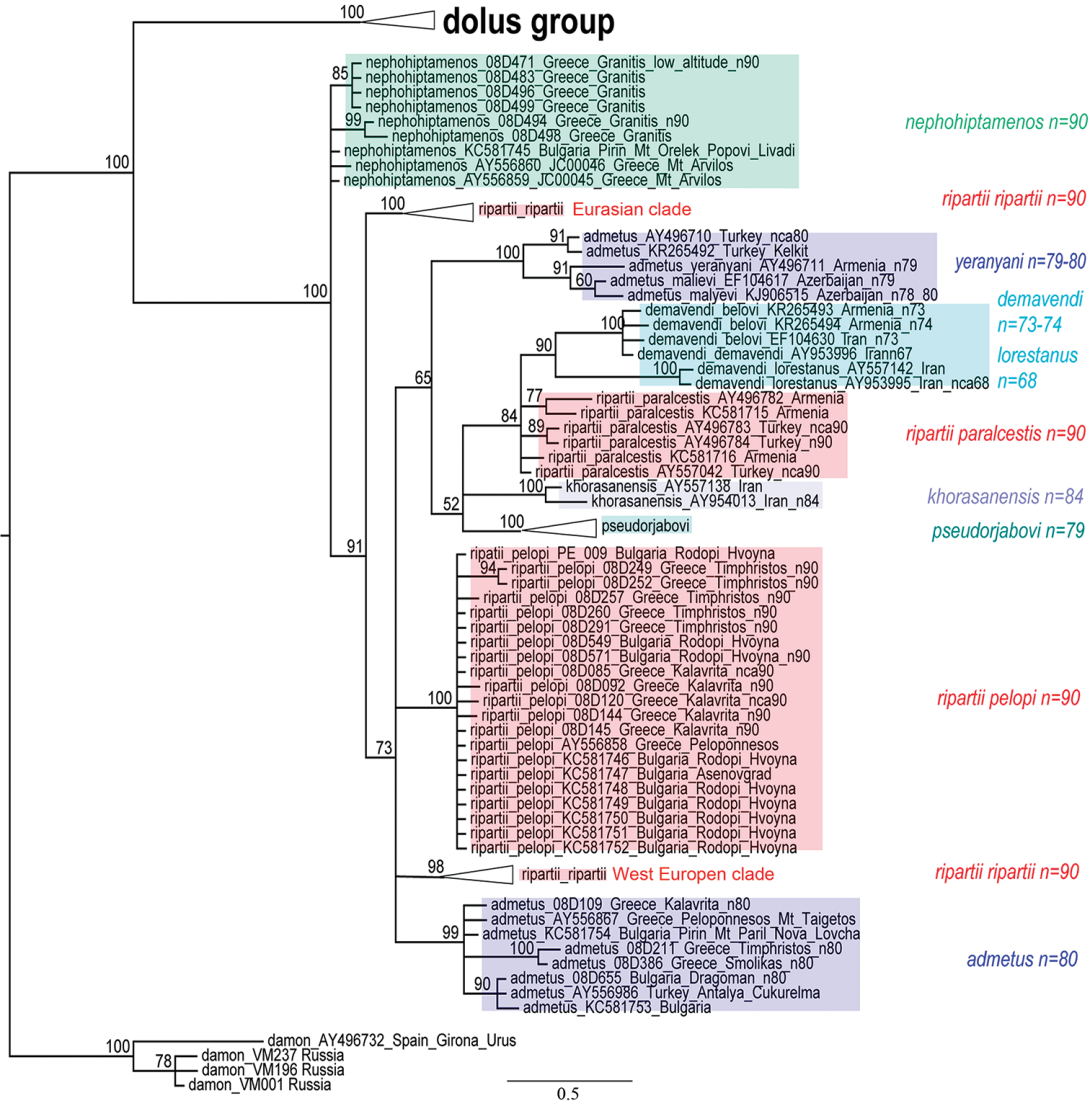
Fragment of the Bayesian tree of *Polyommatus admetus* and *Polyommatus dolus* complexes based on analysis of *COI* barcodes and focused on *Polyommatus nephohiptamenos*, *Polyommatus admetus* and *Polyommatus ripartii pelopi*. *Polyommatus pseudorjabovi* clade is not shown in details, for its composition see Lukhtanov et al. (2015a). The West-European and the “mixed” (Eurasian) clades of *Polyommatus ripartii* are shown in Fig. 7. *Polyommatus dolus* group is shown in Fig. 8. Numbers at nodes indicate Bayesian posterior probability.

**Figure 7.**
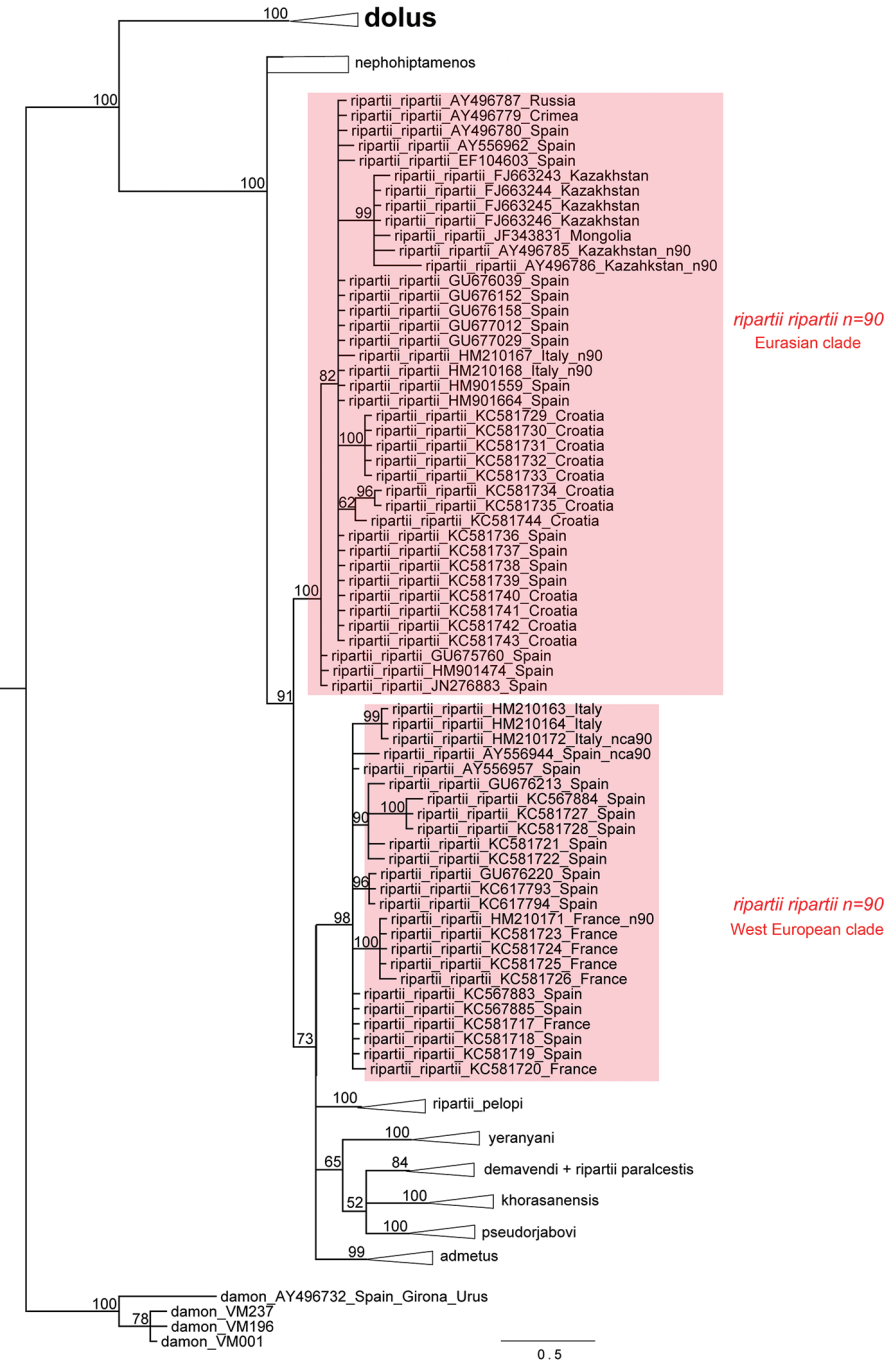
Fragment of the Bayesian tree of *Polyommatus admetus* and *Polyommatus dolus* complexes based on analysis of *COI* barcodes and focused on details of the West-European and the “mixed” (Eurasian) clades of *Polyommatus ripartii*. Numbers at nodes indicate Bayesian posterior probability.

**Figure 8.**
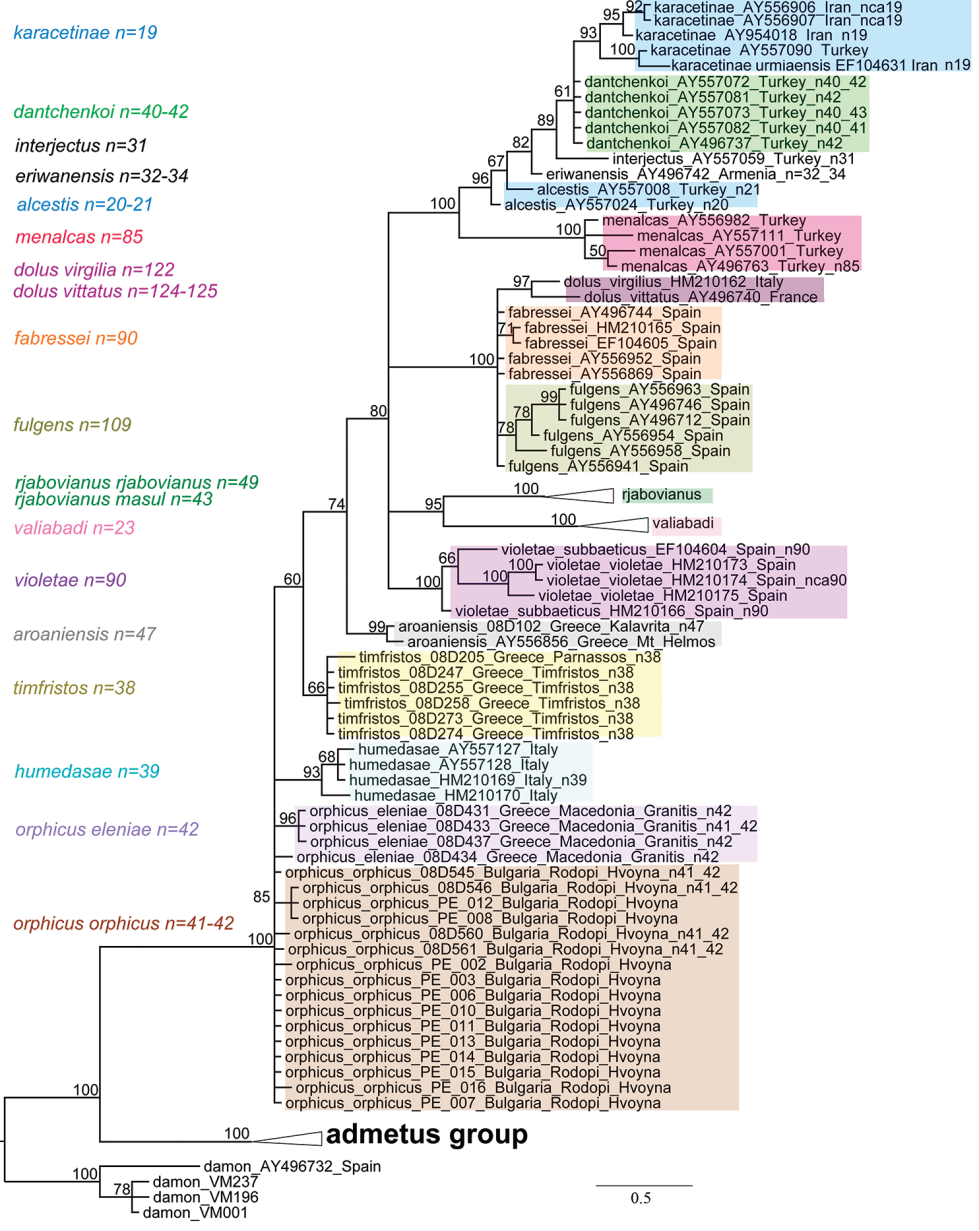
Fragment of the Bayesian tree based on analysis of *COI* barcodes and focused on details of the *Polyommatus dolus* group. *Polyommatus rjabovianus* and *Polyommatus valiabadi* clades are not shown in details, for their composition see Lukhtanov et al. (2015a). Numbers at nodes indicate Bayesian posterior probability.

Bayesian analysis of the 657-bp region of *COI* gene resulted in a phylogram, showing a high level of posterior probability for the majority of the revealed clades. Analysis of the 221-specimen dataset recovered the *Polyommatus admetus* and *Polyommatus dolus* species groups as distinct monophyletic lineages. This is consistent with the previous conclusions (Wiemers 2003, Kandul et al. 2004, 2007, Lukhtanov et al. 2005, 2015, Vila et al. 2010, Dincă et al. 2013a). The tree divided into two parts (*Polyommatus admetus* and *Polyommatus dolus* groups) is shown in Figures 6–8.

Within the *Polyommatus admetus* group, the species *Polyommatus ripartii* appeared as a polyphyletic assemblage consisting of four monophyletic lineages: the “Balkan” clade, including specimens from Greece and Bulgaria, “West-European” clade, including butterflies from France, Italy and Spain, “mixed” (or Eurasian) clade, including butterflies distributed from Spain to Mongolia, and Turkish-Transcaucasian clade, including butterflies from Turkey and Armenia. The last clade formed an independent lineage, sister to the species *Polyommatus demavendi* (Pfeiffer, 1938) from east Turkey, Transcaususus and Iran.

*Polyommatus admetus* sensu auctorum formed two independent clades: one consisting of European and west Turkish specimens and another consisting of specimens from east Turkey, Armenia and Azerbaijan. *Polyommatus nephohiptamenos* appeared on the Bayesian tree as a paraphyletic group consisting of nine weakly differentiated individuals. On the MP and ML trees (Figs 18 and 21 in Appendix 2), *Polyommatus nephohiptamenos* tended to form a monophyletic clade, but the bootstrap support of this clade was very low.

The *Polyommatus dolus* group is interesting for its Balkan species position. *Polyommatus aroaniensis* formed an independent clade separate from *Polyommatus timfristos* sp. n., which formed a monophyletic clade as well. Specimens of *Polyommatus orphicus orphicus* and *Polyommatus orphicus eleniae* were closely related and formed together a paraphyletic cluster.

**Figure 9.**
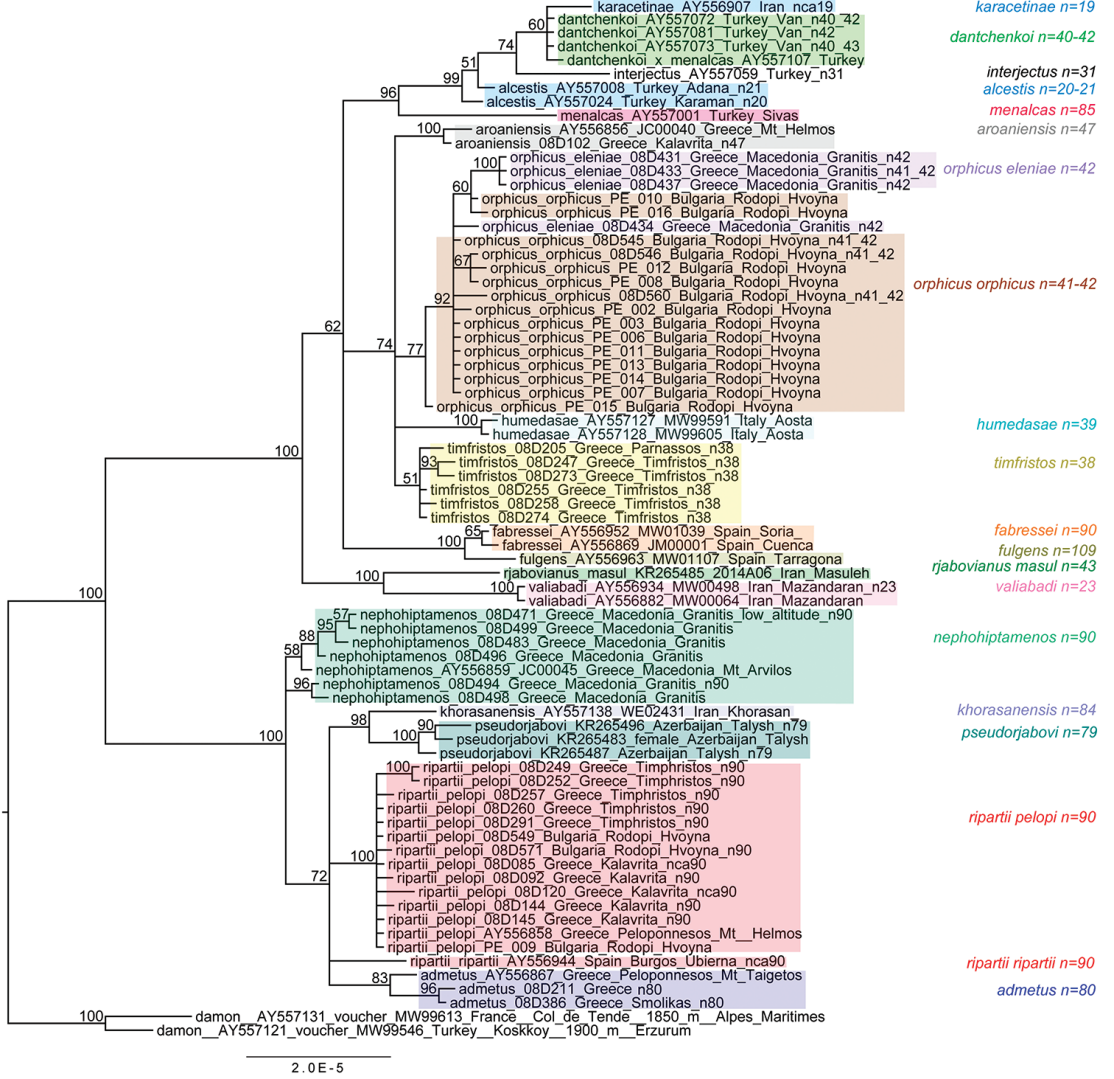
Bayesian tree of *Polyommatus admetus* and *Polyommatus dolus* complexes based on analysis of concatenated alignment (*COI*+*ITS2*). Numbers at nodes indicate Bayesian posterior probability

Because of low variability, it was difficult to use *ITS2* as a single marker to construct the phylogeny of *Agrodiaetus*. Therefore, we decided to combine the sequence data on *COI* and *ITS2* and constructed a tree on the base of these two markers (Fig. 9). We used 75 specimens for which we had data on both markers. Total length of the combined sequence was 1039 bp. The Bayesian tree constructed on the base of the concatenated alignment revealed generally the same topology as in the case of *COI* tree, however with a higher support for few clades, and *Polyommatus orphicus orphicus* + *Polyommatus orphicus elenia* formed a monophyletic clade with a posterior probability value 77.

### Haplotype network analysis

**Figure 10.**
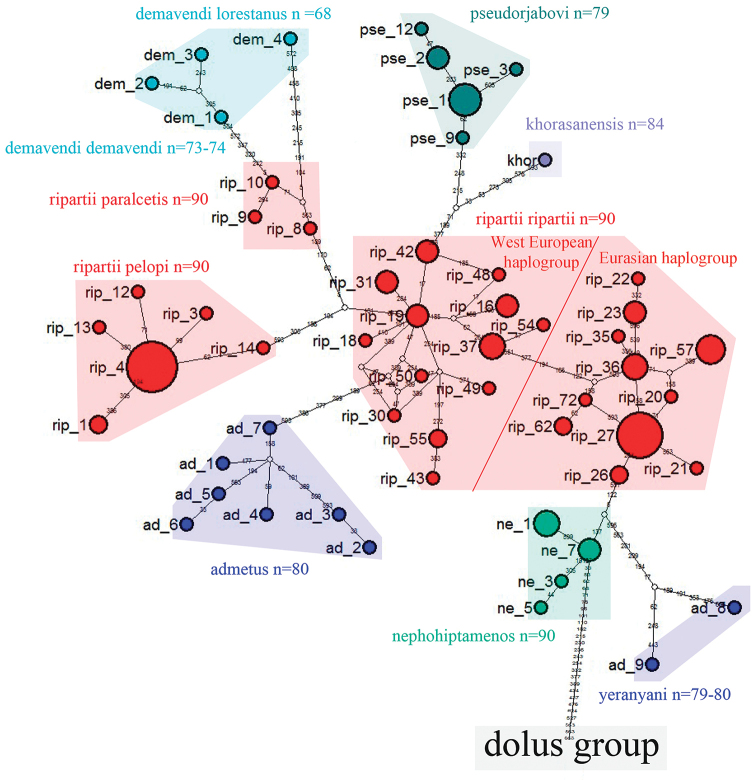
Haplotype network of *Polyommatus admetus* species group. Colored circles represent different taxa. Each line segment represents a mutation step, and white small circles represent “missing” haplotypes.

**Figure 11.**
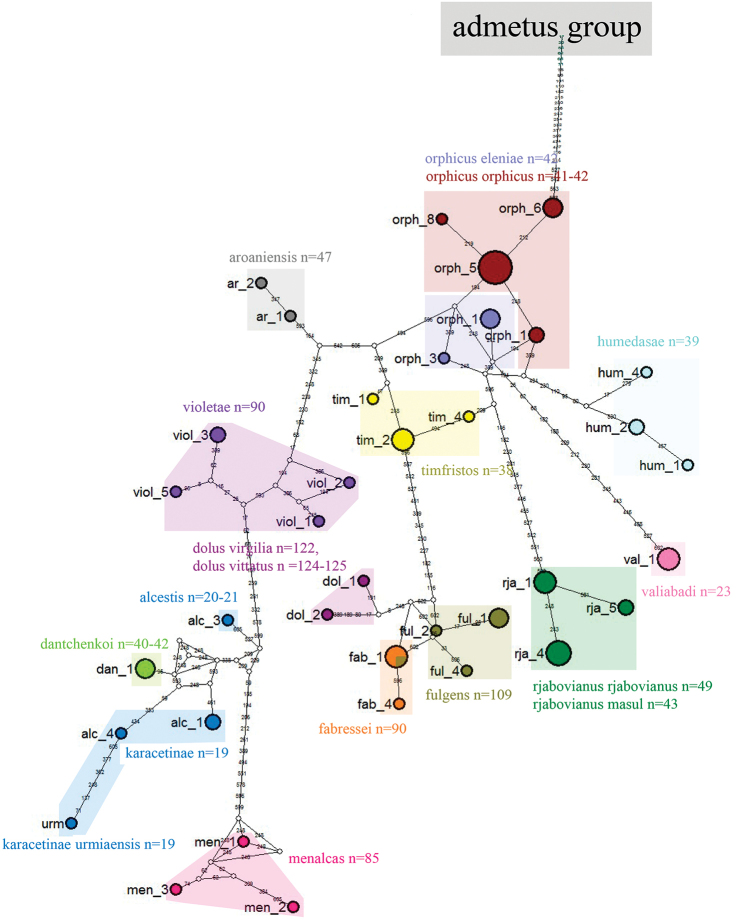
Haplotype network of *Polyommatus dolus* species group. Colored circles represent different taxa. Each line segment represents a mutation step, and white small circles represent “missing” haplotypes.

The complicated relationships between species of *Polyommatus admetus* and *Polyommatus dolus* groups were also reflected by a haplotype network (Figs 10 and 11) constructed on the base of *COI*. To construct the network we used 191 specimens that were collapsed in 96 haplotypes representing 26 haplogroups (Table 2): 10 haplogroups for *Polyommatus admetus* group and 16 haplogroups for *Polyommatus dolus* group.

*Polyommatus ripartii* was represented by 82 specimens divided in 38 haplotypes and four haplogroups which corresponded completely with the four clades revealed on the Bayesian tree (Fig. 10). *Polyommatus admetus* sensu auctorum was found to include two haplogroups. One haplogroup was represented by specimens from the Balkan and west Turkey (*Polyommatus admetus admetus*), and the other haplogroup was represented by specimens from Armenia and Azerbaijan (*Polyommatus admetus yeranyani* + *Polyommatus admetus malievi*). These two haplogoups were clearly distinct from one another as can be seen in the number of nucleotide substitutions between them. *Polyommatus nephohiptamenos* was represented by a distinct haplogroup most close to *Polyommatus ripartii ripartii* haplogroup.

As a by-product of our study, we also discovered that within our samples *Polyommatus demavendi* comprised two haplogroups. One haplogroup was represented by specimens of *Polyommatus demavendi belovi*, whilst the other was represented by *Polyommatus demavendi lorestanus*. *Polyommatus pseudorjabovi* was represented by a single differentiated haplogroup. A distinct haplogroup represented by a single haplotype was found within *Polyommatus khorasanensis*.

Concerning *Polyommatus dolus* group (Fig. 11) we would like to mention that all recognized species, except for *Polyommatus fulgens* and *Polyommatus fabressei*, were represented by clearly distinct *COI* haplogroups. *Polyommatus fulgens* and *Polyommatus fabressei* were closely related and even shared one haplotype, despite clear differences in butterfly wing color and karyotypes.

Haplotypes of our target taxa (*Polyommatus aroaniensis*, *Polyommatus timfristos* sp. n., *Polyommatus orphicus* and *Polyommatus humedasae*) formed together a single cluster. However, all these taxa were distinct, and they did not share any common haplotypes. Therefore, this cluster could be subdivided into four haplogroups: *ar* (*Polyommatus aroaniensis*), *tim* (*Polyommatus timfristos*), *orph* (*Polyommatus orphicus*) and *hum* (*Polyommatus humedasae*) (Table 2, Fig. 11).

Despite presumed conspecifity (Kolev 2005), *Polyommatus orphicus* and *Polyommatus dantchenkoi* were found to be in the opposite parts of the recovered net, being separated by a number of other species (*Polyommatus alcestis*, *Polyommatus violetae*, *Polyommatus aroaniensis*, *Polyommatus timfristos*). The chromosomally distinct taxa *Polyommatus alcestis* and *Polyommatus karacetinae* were found to be also distinct with respect to their *COI* haplotypes. These two taxa were already treated as different species by Wiemers et al. (2009).

### Butterfly morphology

**Figure 12.**
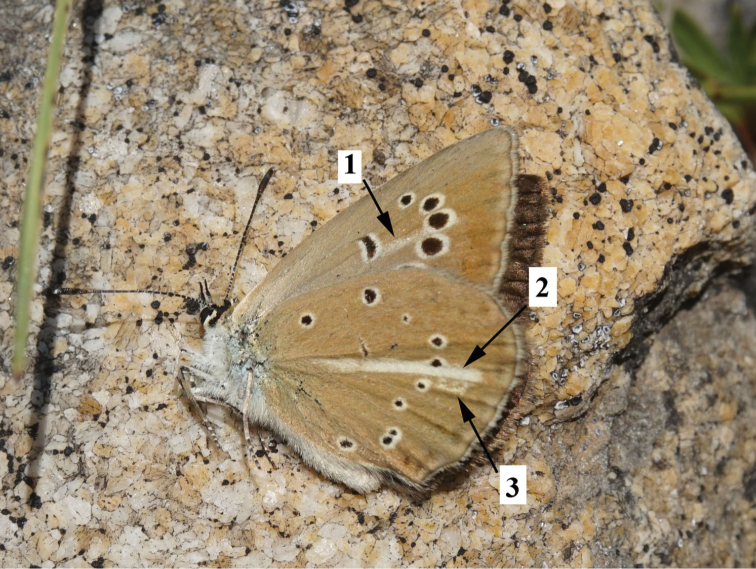
*Polyommatus orphicus orphicus* collected in the type locality (Bulgaria, Hvoyna, 3 July 2016). Photo by E. Pazhenkova. White postdiscal streak between discal spot and submarginal marking on the forewing underside (character 1), prominent white streak on the hindwing underside (character 2) and additional white short streak between postdiscal and submarginal areas of the hind wing underside (character 3) are shown.

One of the main characteristic features of the anomalous blue butterflies is the upperside wing color. All males and females have brown upper side of the wings, and therefore the group is also called “brown” complex. As for the underside (Fig. 12), there are some differentiated characters of the wing pattern that allow the defining of seven morphological types.

**Figure 13.**
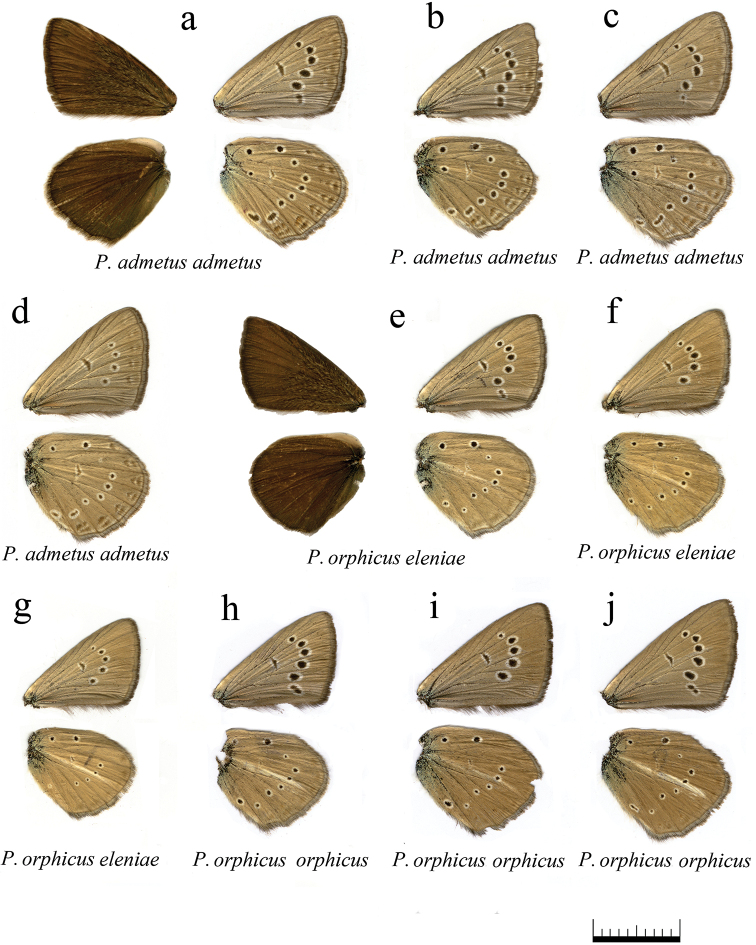
The coloration and wing pattern of *Polyommatus admetus*, *Polyommatus orphicus eleniae* and *Polyommatus orphicus orphicus*. The letters correspond to the following sample numbers: **a** LR-08-D109 upperside and underside **b** LR-08-386 **c** LR-08-655 **d** LR-08-211 **e** LR-08-433 upperside and underside **f** LR-08-434 **g** LR-08-437 **h** LR-08-545 **i** LR-08-546 **j** LR-08-560. Scale bar corresponds to 10 mm in all figures.

**Figure 14.**
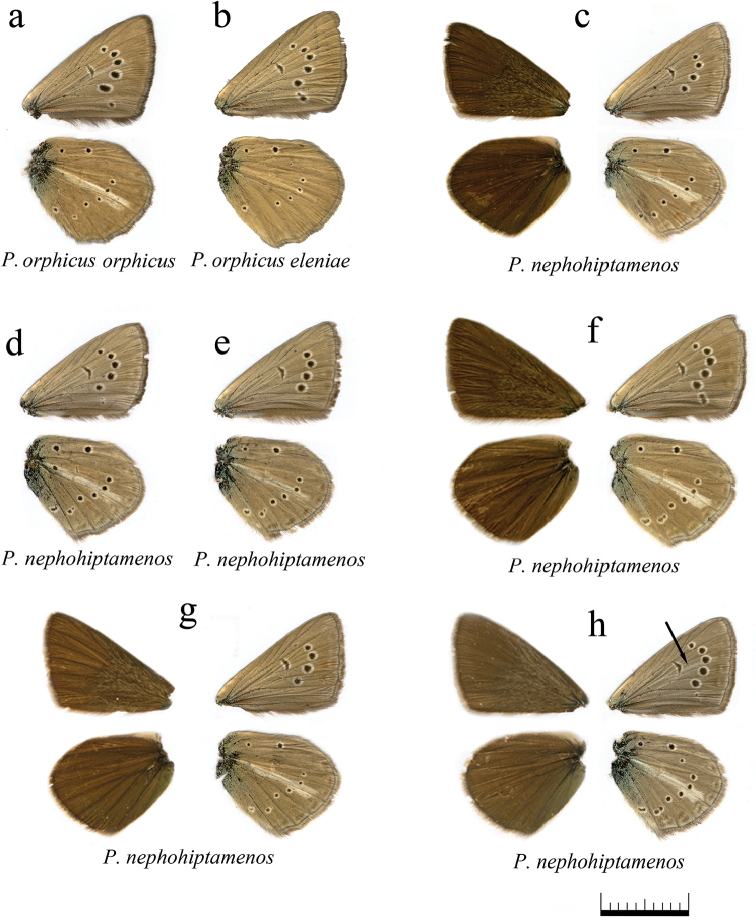
The coloration and wing pattern of *Polyommatus orphicus orphicus*, *Polyommatus orphicus eleniae* and *Polyommatus nephohiptamenos*. The letters correspond to the following sample numbers: **a** LR-08-D561 **b** LR-08-431 **c** LR-08-483 upperside and underside **d** LR-08-496 **e** LR-08-498 **f** LR-08-499 upperside and underside **g** LR-08-485 upperside and underside **h** LR-08-494 upperside and underside, white postdiscal streak between discal spot and submarginal marking on the forewing underside is shown by arrow. Bar = 10 mm.

**Figure 15.**
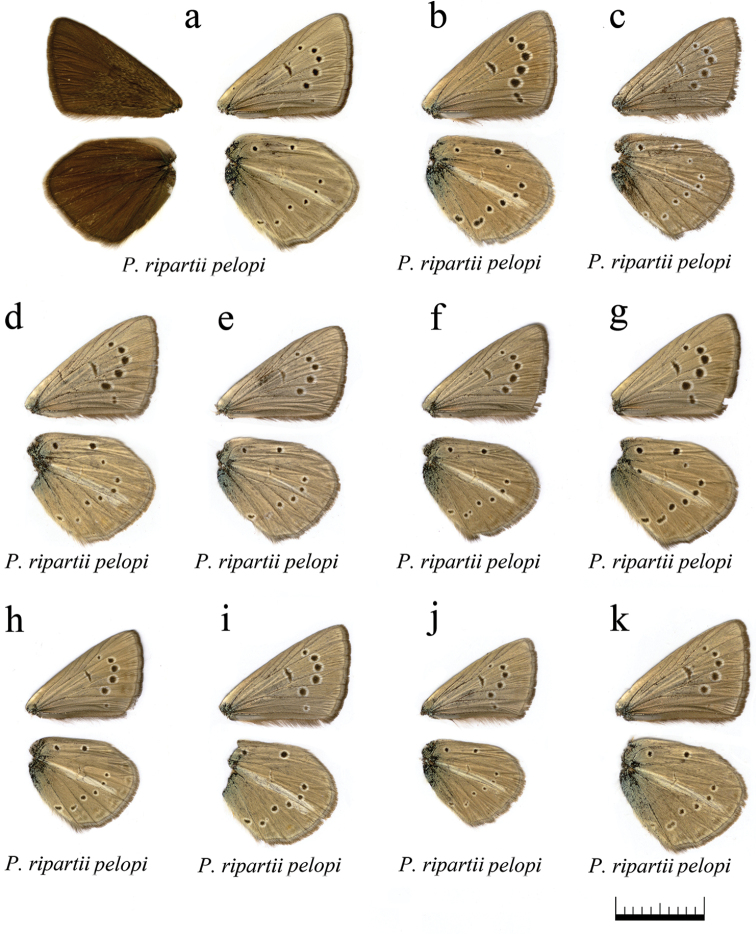
The coloration and wing pattern of *Polyommatus ripartii pelopi*. The letters correspond to the following sample numbers: **a** LR-08-D257 upperside and underside **b** LR-08-471 **c** LR-08-085 **d** LR-08-092 **e** LR-08-120 **f** LR-08-144 **g** LR-08-145 **h** LR-08-249 **i** LR-08-252 **j** LR-08-260 **k** LR-08-291. Bar = 10 mm.

**Figure 16.**
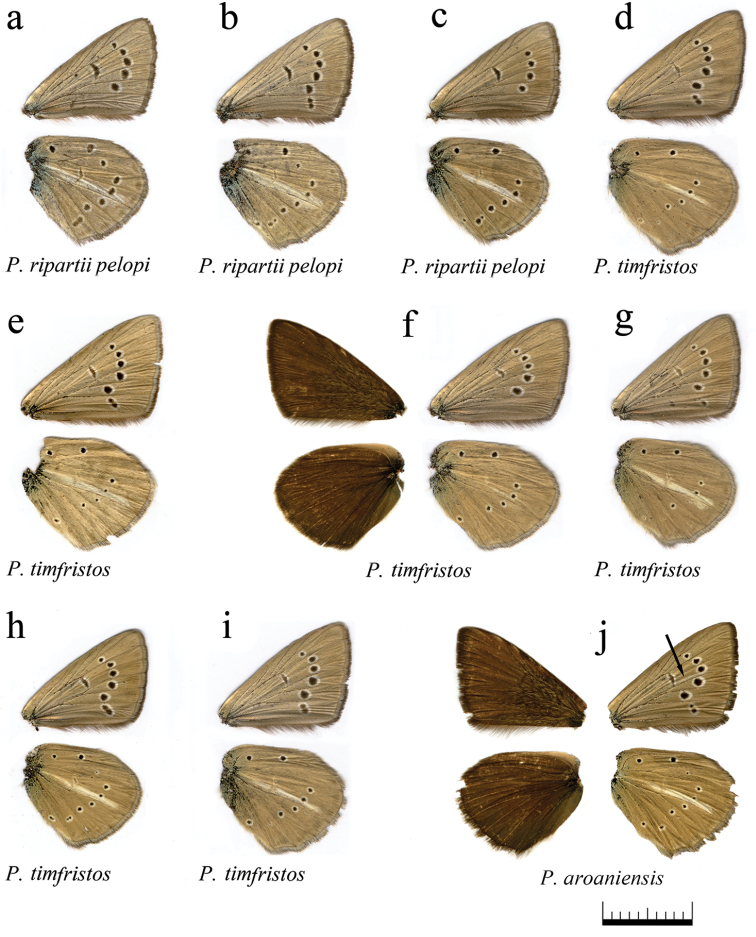
The coloration and wing pattern of *Polyommatus ripartii pelopi*, *Polyommatus timfristos* sp. n. and *Polyommatus aroaniensis*. The letters correspond to the following sample numbers: **a** LR-08-D549 **b** LR-08-551 **c** LR-08-571 **d** LR-08-273 **e** LR-08-205 **f** LR-08-274 upperside and underside **g** LR-08-258 **h** LR-08-247 (Holotype) **i** LR-08-255 **j** LR-08-102 upperside and underside. White postdiscal streak between discal spot and submarginal marking on the forewing underside is shown by arrow. Bar = 10 mm.

1. *Polyommatus ripartii* type: hindwing underside with well-developed white streak (character 2 in Fig. 12), spots are small or medium-sized, marginal marking is reduced. This type is found in different species of both *Polyommatus admetus* and *Polyommatus dolus* complexes, e.g. in *Polyommatus orphicus orphicus* (Fig. 13g), *Polyommatus orphicus eleniae* (Fig. 13j), *Polyommatus nephohiptamenos* (Fig. 14e), *Polyommatus ripartii pelopi* (Figs 15a, f, g, h, j, k, 16a, b, c) and *Polyommatus timfristos* (Fig. 16e, h).
2. *Polyommatus valiabadi* type: the wing underside with exaggerated spots, white streak on the hindwing underside is clearly visible and sharp. This type is found in *Polyommatus valiabadi*, *Polyommatus rjabovianus* and *Polyommatus pseudorjabovi* from Iran and Azerbaijan (Lukhtanov et al. 2015). This type is not found in European *Agrodiaetus* species.
3. *Polyommatus admetus* type: the hindwing has no white streak, marginal marking is very well pronounced. This type is found in *Polyommatus admetus* (Fig. 13a, b, c, d).
4. *Polyommatus nephohiptamenos* type: White streak is well pronounced and very broad on the hindwing, consisting of the main streak and an additional short streak between postdiscal and submarginal areas, just under the main streak. This type is common in *Polyommatus nephohiptamenos* (Fig. 14c, d, f, g, h), not rare in *Polyommatus ripartii* (Fig. 15b,d,i) and also found in *Polyommatus orphicus orphicus* (Fig. 14a) and *Polyommatus timfristos* (Fig. 16g).
5. *Polyommatus humedasae* type: no white streak on the hindwing, marginal marking is pale. This type is quite common in *Polyommatus aroaniensis*, *Polyommatus timfristos* (Fig. 16f) and *Polyommatus orphicus* (Fig. 14b). It is typical for some populations of *Polyommatus ripartii* from West Europe (Vila et al. 2010) (but not from the Balkan Peninsula).
6. *Polyommatus aroaniensis* type: the white streak on the hindwing underside demonstrates different level of reduction. This type is found in *Polyommatus aroaniensis* (Fig. 16j), *Polyommatus timfristos* (Fig. 16d, e, i), *Polyommatus orphicus orphicus* (Fig. 14h, i) and *Polyommatus orphicus eleniae* (Fig. 13e, g). It is also found in the population of *Polyommatus ripartii* from the Crimea (Vila et al. 2010) (but not from the Balkan Peninsula).
7. *Polyommatus orphicus* type: forewing underside with clear white postdiscal streak between discal spot and submarginal marking, white streak on hindwing underside is prominent, often with additional small white streak (Fig. 12). This type is common in *Polyommatus orphicus orphicus* (Fig. 14a); nevertheless, the most characteristic feature (the white postdiscal streak between discal spot and submarginal marking on the forewing underside) can be found in other species, e.g. *Polyommatus aroaniensis* (Fig. 16j) and *Polyommatus nephohiptamenos* (Fig. 14h).

## Species level monophyly, paraphyly and polyphyly

The studied taxa were found to demonstrate a relatively low level of *COI* and *ITS2* differentiation in terms of genetic distances between species and numbers of evolutionary steps between the taxa on haplotype network (Figs 10 and 11). This result is not unexpected in light of our previous knowledge of this group (Wiemers and Fiedler 2007).

The low genetic differentiantion results in relatively low support for some recovered clades (e.g. for *Polyommatus timfristos*, Figs 8 and 9) and in non-monophyly of some taxa (*Polyommatus nephohiptamenos*, *Polyommatus orphicus*) with respect to *COI* gene or to combination of *COI* and *ITS2*. Species-level non-monophyly in DNA barcode gene trees can have multiple explanations (Mutanen et al. 2016). In our case, combination of low interspecific differentiation with low level of intraspecific variation indicates that preservation of ancestral polymorphism and incomplete lineage sorting (rather than interspecific hybridization) is the most likely mechanism explaining the pattern observed. This finding is also in agreement with the previous conclusion that the subgenus Agrodiaetus itself and its species represent young evolutionary entities (Kandul et al. 2004). We should also stress that despite the obvious paraphyly, the taxa *Polyommatus nephohiptamenos* and *Polyommatus orphicus* are distinct with respect to the *COI* barcodes, and this can be seen on both Bayesian tree (Figs 6–8) and haplotype network (Figs 10 and 11).

An entirely different situation was found in *Polyommatus ripartii* and *Polyommatus admetus* sensu auctorum. In these taxa polyphyly in *COI* trees arises as a result of deep intraspecific divergence. There are two theoretically possible explanations for this kind of non-monophyly. First, each taxon can be a mix of unrecognized multiple species (Dincă et al. 2011, 2013b). Second, a profound irregularity in barcodes can be caused by reasons other than speciation resulting in extraordinary intra-specific barcode variability (Pazhenkova and Lukhtanov 2016). Among these reasons, interspecific mitochondrial introgression (Lukhtanov et al. 2015b) and blending of deeply diverged mitochondrial lineages which evolved in allopatry in different Pleistocene refugia (Pazhenkova and Lukhtanov 2016) are most likely ones. The first explanation could be applied to *Polyommatus admetus* sensu auctorum which most probably comprises two allopatric species, *Polyommatus admetus* sensu stricto and *Polyommatus yeranyani* (see the section **Taxonomy** below). The situation with *Polyommatus ripartii* sensu lato seems to be much more complicated. A combination of the first and the second explanations could be applied to *Polyommatus ripartii* sensu lato, and West-European and Eurasian clades could represent sympatric (parapatric?) intraspecific lineages (Dinca et al. 2013) whereas Turkish-Transcaucasian clade could represent an allopatric species. Additioanl studies are required to solve this problem.

## Chromosomal diversity

### *Polyommatus admetus*

The chromosome number of *Polyommatus admetus* was first established by H. de Lesse who discovered n=80 in populations from Bulgaria (Kalotina) and W Turkey, and n=78-80 (with predominance of n=79) in populations from the eastern part of Turkey (de Lesse 1960a,b). The last count (n=78-80 with predominance of n=79) was later confirmed for populations from Armenia (Lukhtanov and Dantchenko 2002a), Turkey and Azerbaijan (Dantchenko and Lukhtanov 2005, Lukhtanov et al. 2015a). Here we confirm the haploid chromosome number n=80 for Dragoman near Kalotina (Bulgaria) and demonstrate that this karyotype occurs in other localities in Greece. The karyotype of the European samples (with predominance of n=80) seems to be similar, but not completely identical to the karyotype of samples from east Turkey, Armenia and Azerbaijan (with predominance of n=79).

### *Polyommatus ripartii*

This transpalearctic species has been known to have a stable karyotype (n=90, including one large, one medium and 88 small elements) throughout its whole distribution range from Spain in the west to the Altai in the east (de Lesse 1960a,b, Kandul 1997, Lukhtanov and Datchenko 2002, Vila et al. 2010, Vershinina and Lukhtanov 2010, Przybyłowicz et al. 2014). The number n=90 was also found in *Polyommatus ripartii pelopi* (Coutsis et al. 1999), and we confirmed this count for samples from South and Central Greece and from Bulgaria.

### *Polyommatus nephohiptamenos*

The haploid chromosome number was erroneously given for this taxon as n=8-11 by Brown and Coutsis (1978), and later corrected by Coutsis and De Prins (2007) who established the chromosome number with an approximation due bivalents overlaps as n=ca84-88. Here we were able to make a precise count of chromosome elements in this taxon and to demonstrate that n=90, exactly as in *Polyommatus ripartii*. We do not confirm the proposed difference between *Polyommatus nephohiptamenos* and *Polyommatus ripartii* in number of large chromosomes (Coutsis and De Prins 2007). In our squash preparations, both species demonstrate one big and one medium-sized element in the haploid chromosome set.

### *Polyommatus aroaniensis*

The haploid chromosome number for this taxon was erroneously given as n=15-16 by Brown (1976a), and later corrected to be n=48 in few studied metaphase plates by Coutsis et al. (1999). In the single studied sample we were able to make a precise count of chromosome elements and found the haploid chromosome number to be n=47. Both counts (previous n=48 and n=47 in this study), are essentially different from those found in closely related *Polyommatus timfristos* and *Polyommatus orphicus* (Kolev 2005, this work) and *Polyommatus humedasae* (Troiano et al. 1979, Vila et al. 2010).

### *Polyommatus orphicus* and *Polyommatus eleniae*

The chromosome number of *Polyommatus orphicus* was first established by Kolev (2005) who discovered n=41-42 in population from Hvoyna (Bulgaria), thus, similar to the karyotype found in *Polyommatus dantchenkoi* from remote east Turkey (Lukhtanov et al. 2003).

The chromosome number of *Polyommatus eleniae* was established first by Coutsis and De Prins (2005) who discovered n=41 in population from Falakro Mt near Granitis (Greece). Coutsis and De Prins reported that despite identical chromosome number, karyotypes of *Polyommatus orphicus* and *Polyommatus eleniae* were different in respect to their structure. Karyotype of *Polyommatus eleniae* was reported to be more asymmetrical than karyotype of *Polyommatus orphicus* (that is, the chromosomes were more differentiated with respect to their size).

Here we reinvestigated the karyotypes of *Polyommatus orphicus* and *Polyommatus eleniae* originating directly from their type-localities. Our data confirm previous chromosome number counts, but do not confirm the differences in karyotype structures. In our opinion, the presumed differences could appear because of differences in staining techniques used by Kolev (2005) for *Polyommatus orphicus* and Coutsis and De Prins (2005) for *Polyommatus eleniae* (see Wiemers and De Prins 2004). In our study, we used the same technique for both taxa, and we did not find any differences in the karyotype structure.

### *Polyommatus timfristos*

The haploid chromosome number of this taxon is established first here as n=38 and thus differs by at least three fixed chromosome fussions/fixions from *Polyommatus orphicus orphicus* and *Polyommatus orphicus eleniae* (n=41-42). This number is similar (but not identical) to that found in *Polyommatus humedasae* (n=39, Vila et al. 2010). We are not sure that the karyotypes of *Polyommatus timfristos* and *Polyommatus humedasae* are related in their origin because they are not found in proximity and separated by an area where *Polyommatus orphicus* with n=41–42 is distributed.

## Taxonomy

### *Polyommatus admetus*

The Balkan and west Turkish populations of *Polyommatus admetus* have a unique hindwing underside pattern (*Polyommatus admetus* type, Fig. 13a, b, c, d) and can be easily separated on the basis of morphology from other species. However, some taxonomic and identification problems appear if oriental populations of *Polyommatus admetus* sensu lato are considered. In 2004, *Polyommatus admetus yeranyani* from Armenia and *Polyommatus admetus malievi* from Azerbaijan were described (Dantchenko and Lukhtanov 2005). The two last taxa differ from the nominative subspecies morphologically. They usually have a distinct white streak on the underside of the hindwing, and the marginal pattern of the wing underside is not as prominent as in *Polyommatus admetus admetus*. In fact, *Polyommatus admetus yeranyani* and *Polyommatus admetus malievi* are phenotypically similar to *Polyommatus ripartii* and *Polyommatus demavendi*, and their identification is not always easy. Karyological analysis revealed a minor difference between the western and oriental forms (see above), and molecular analysis demonstrated that they were differentiated with respect to *COI* barcodes and did not constitute together a monophyletic entity. This barcode distinctness is especially clearly expressed in the haplotype network (Fig. 10). Therefore, in accordance with the criterion of avoiding non-monophyletic groups in taxonomy (Vila et al. 2013), they should be treated as distinct species *Polyommatus admetus* and *Polyommatus yeranyani*.

### *Polyommatus ripartii*

The distribution of *COI* haplotypes in *Polyommatus ripartii* demonstrates a very complex picture. This taxon is represented by several clades on the phylogenetic reconstructions. The West-European clade includes butterflies from France, Italy and Spain. Another clade (a “mixed”, or Eurasian clade) includes butterflies from the whole Western Palaearctic region from Spain to Mongolia. Eastern Turkish-Caucasian clade (*Polyommatus ripartii paralcestis*) is strongly differentiated and appears as a group close to *Polyommatus demavendi*. Complicated taxonomy and phylogeography of *Polyommatus ripartii* have recently been topics of several specific studies and publications (Vila et al. 2010, Vodolazhsky et al. 2011, Dincă et al. 2013a, Przybyłowicz et al. 2014) and are out of the focus of the present paper. The sequences obtained in our study confirm that Balkan samples represent one of the major clades within *Polyommatus ripartii* populations, thus *Polyommatus ripartii pelopi* is confirmed as a valid subspecies.

### *Polyommatus nephohiptamenos*

Taxonomic interpretation of this local Balkan endemic is difficult since it is morphologically very similar and chromosomally seems to be identical to the close species *Polyommatus ripartii*. However, distinct *COI* barcodes in combination with ecological differentiation (*Polyommatus nephohiptamenos* is a high altitude species, whereas *Polyommatus ripartii pelopi* can be found usually at middle and low elevations) do not allow us to reject the pre-existing taxonomic hypotheis that *Polyommatus nephohiptamenos* represents a distinct taxonomic entity. The fact that *Polyommatus nephohiptamenos* retains its homogeneity with respect to *COI* being surrounded by closely related *Polyommatus ripartii* is additional indirect evidence for a presence of genetic boundaries between them. Further molecular and genetic studies are required to understand the real taxonomic status of *Polyommatus nephohiptamenos*.

### *Polyommatus orphicus*

*Polyommatus dantchenkoi orphicus* was described (Kolev 2005) and later considered (Tshikolovets 2011, Eckweiler and Bozano 2016) as a subspecies of *Polyommatus dantchenkoi*, a species known from east Turkey, because *Polyommatus dantchenkoi dantchenkoi* and *Polyommatus dantchenkoi orphicus* shared a similar phenotype and number of chromosomes (Lukhtanov et al. 2003, Kolev 2005). At times, *Polyommatus orphicus* has been considered as a distinct species (e.g. Van Swaay et al. 2010); however, its species level status was not justified.

Our molecular data demonstrate that, despite similarity in number of chromosomes, *Polyommatus dantchenkoi dantchenkoi* and *Polyommatus dantchenkoi orphicus* are not closely related as was previously thought. In the haplotype network, these taxa were found to be placed in the opposite parts of the recovered net, being separated by a number of other species (Fig. 11). Their merging would result in a polyphyletic assemblage (Fig. 8). Avoiding non-monophyletic groups is a preferable option in practical taxonomy (Talavera et al. 2013a). Therefore, *Polyommatus dantchenkoi* and *Polyommatus orphicus* should be considered as two distinct species. We should also note that the *COI* barcodes alone (as in our study) can provide weak evidence for monophyly or non-monophyly of taxa since trees inferred from single markers sometimes display relationships that reflect the evolutionary histories of individual genes rather than of the species being studied. In case of *Agrodiaetus*, *COI* barcodes showing such a discrepancy between species and gene trees may be a result of interspecific mitochondrial introgression (Lukhtanov et al. 2008, 2015b). Despite this limitation, we argue that monophyletic clusters resulting from the DNA barcode analysis are better primary taxonomic hypotheses than para- or polyphyletic ones (Lukhtanov et al. 2016).

*Polyommatus eleniae* was described from a place located 80 km south-west from the type locality of *Polyommatus orphicus*. *Polyommatus orphicus* and *Polyommatus eleniae* have the same number of chromosomes, but it was supposed that they were different in karyotype structure (Coutsis and De Prins 2005). Additionally, it was supposed that *Polyommatus eleniae* differed from *Polyommatus orphicus* by the constant lack of a white postdiscal streak on the forewing underside (character 1 on Fig. 12) and by strong reduction or total lacking of a white streak on the hindwing underside (character 2 on Fig. 12) (Coutsis and De Prins 2005). In *Polyommatus orphicus* these streaks are supposed to be always sharply defined (Kolev 2005). Our study does not support the difference in karyotypes (see above). Our analyses showed that the supposed differences in morphology disappeared if individual variations were taken into account. Although the “typical” phenotype of *Polyommatus orphicus* (Figs 12 and 14a) often present in in Hvoyna, the individuals with different level of reduction of white streak on both fore- and hindwing underside are very common (Fig. 13h, i, j). These individuals with confidence can be identified as *Polyommatus orphicus* as they have the same karyotype and do not differ in mitochondrial haplotypes. Thus, the morphological difference between individuals from Hvoyna (*Polyommatus orphicus*) and Falakro Mt (*Polyommatus eleniae*) is not clear and is not based on fixed characters. The difference in karyotypes was also not confirmed in our analysis (see the section **Chromosomal diversity** above). Therefore, we conclude that the population from Falakro Mt is most probably conspecific with *Polyommatus orphicus* and can be treated as a subspecies *Polyommatus orphicus eleniae*.

### *Polyommatus aroaniensis*

This taxon was first described by Brown (1976a) as a subspecies of *Polyommatus alcestis* and two years later was raised to species rank (Brown and Coutsis 1978). Despite its similarity to other taxa of the brown complex, especially with *Polyommatus humedasae*, *Polyommatus orphicus orphicus* and *Polyommatus orphicus eleniae*, it differs by its karyotype and *COI* barcodes. Its species distinctness confirmed by chromosomal analysis (Coutsis et al. 1999) has never been questioned. Thus, there has been no problem with treatment of *Polyommatus aroanisnsis* as a separate species. However, there are numerous identification problems associated with *Polyommatus aroaniensis* because several populations from Central and Northern Greece, as well as from other countries of the Balkan Peninsula were identified as *Polyommatus aroaniensis* (see the section Distribution areas below), but their karyotypes were not studied. In our work, we discovered that two of these populations (from Timfristos Mt and Parnassos Mt) represented a previously unrecognized species. Below we name it and provide its formal description.

### Polyommatus (Agrodiaetus) timfristos

Taxon classificationAnimaliaLepidopteraLycaenidae

Lukhtanov, Vishnevskaya & Shapoval sp. n.

http://zoobank.org/58B77480-1FD0-423B-8FF9-BF39E79F177C

#### Holotype

(Fig. 16h). male, field code LR-08-247, GenBank accession number KY066725 for *COI* and KY081279 for *ITS2*; Greece, Timfristos Mt, Karpenisi, 38°55.460'N; 21°47.605 E, 1270 m, 20 July 2008, V.A. Lukhtanov and N.A. Shapoval leg., deposited in Zoological Institute of the Russian Academy of Science (St. Petersburg).

**COI barcode sequence of the holotype**, 657 base pairs.

ACATTATATTTTATTTTTGGAATTTGAGCAGGAATAGTAGGAACATCTCTAAGAATTTTAATTCGTATGGAATTAAGAACTCCTGGATCCTTAATTGGAAATGATCAAATTTATAATACTATTGTTACAGCCCATGCATTTATTATAATTTTTTTTATGGTTATACCTATTATAATTGGAGGATTTGGTAACTGATTAGTTCCCTTAATATTAGGAGCACCTGATATAGCTTTTCCACGATTAAATAATATGAGATTTTGATTATTACCGCCATCATTAATACTACTAATTTCTAGAAGAATTGTAGAAAATGGAGCAGGAACAGGATGAACAGTTTACCCCCCACTTTCATCAAATATTGCACATGGAGGATCATCTGTAGATTTAGCAATTTTCTCTCTTCATTTAGCGGGAATTTCTTCAATTTTAGGAGCAATTAATTTTATTACAACTATCATTAATATACGAGTAAATAATTTATCTTTTGATCAAATATCATTATTTATTTGAGCAGTGGGAATTACAGCATTATTATTACTTTTATCATTGCCTGTATTAGCTGGGGCAATTACCATATTATTAACAGATCGAAATCTTAATACCTCATTCTTTGACCCAGCTGGTGGAGGAGATCCAATTTTATATCAACATTTATTT

Haploid chromosome number of the holotype n=38 (Fig. 4c).

#### Paratypes.

Four males, field codes LR-08-255, LR-08-258, LR-08-273, LR-08-274, forewing length 17–18 mm, the same data as holotype. Male: field code LR-08-205, Greece, Parnassos, 38°33.311'N; 22°34.300 E, 1750 m, 19 July 2008, V.A. Lukhtanov and N.A.Shapoval leg. Five females: forewing length 15–16 mm; Greece, Timfristos Mt, Karpenisi, 38°55.554'N; 21°48.460 E, 1490 m, 21 July 2008, V.A. Lukhtanov and N.A. Shapoval leg. Two females: forewing length 14.5–15.5 mm; Greece, Parnassos, 38°33.311'N; 22°34.300 E, 1750 m, 19 July 2008, V.A. Lukhtanov and N.A. Shapoval leg.. All paratypes are deposited in Zoological Institute of the Russian Academy of Science (St. Petersburg).

#### Males

(Fig. 16d–i). Forewing length 16.2–18.2 mm. Upperside: ground color completely brown. Discoidal, submarginal and antemarginal marking absent on both fore- and hindwings. Forewings with a developed sex brand and scaletuft. Fringe brown as ground color.

Underside: ground color light brown with yellowish coffee-milk tint. Greenish blue basal suffusion very slight, nearly lacking. One basal black spot is present only on hindwings. Discoidal black spot is present on the forewings, but can be slightly seen on the hindwings (absent or vestigial). Postdiscal black ocelli are encircled by a whitish border. They are prominent on the forewings, forming a strongly curved row. Postdiscal black ocelli on the hindwing small. Submarginal and antemarginal marking is absent on the forewings, and absent or vestigial on the hindwings. White streak on hindwings clearly visible. In one specimen the white streak is vestigial, in one the white streak is almost absent (can be slightly distinguished), and in one specimen there is an additional short streak between postdiscal and submarginal areas of the wing, straight under the main white streak. Fringe brown, slightly darker than the underside ground color.

Genitalia: the male genitalia have a structure typical for other species of the subgenus Agrodiaetus (Coutsis, 1986).

#### Females

**Figure 17.**
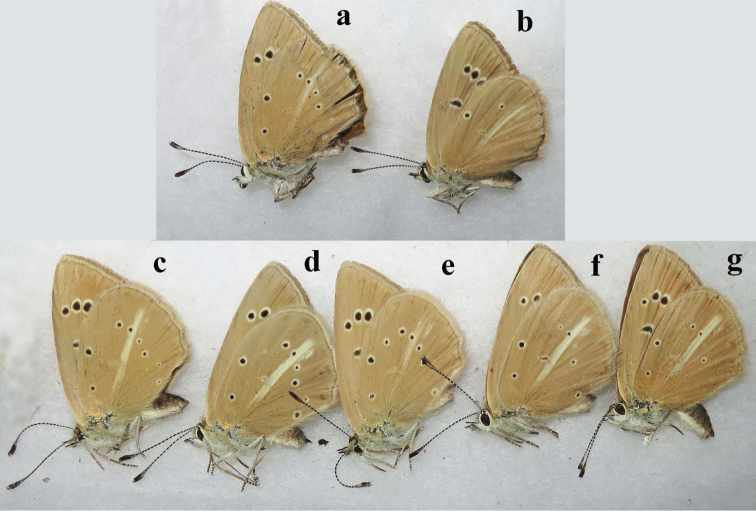
Paratypes of *Polyommatus timfristos* sp. n. (females). **a, b** samples from Parnassos Mt **c–g** samples from Timfristos Mt.

**Figure 18.**
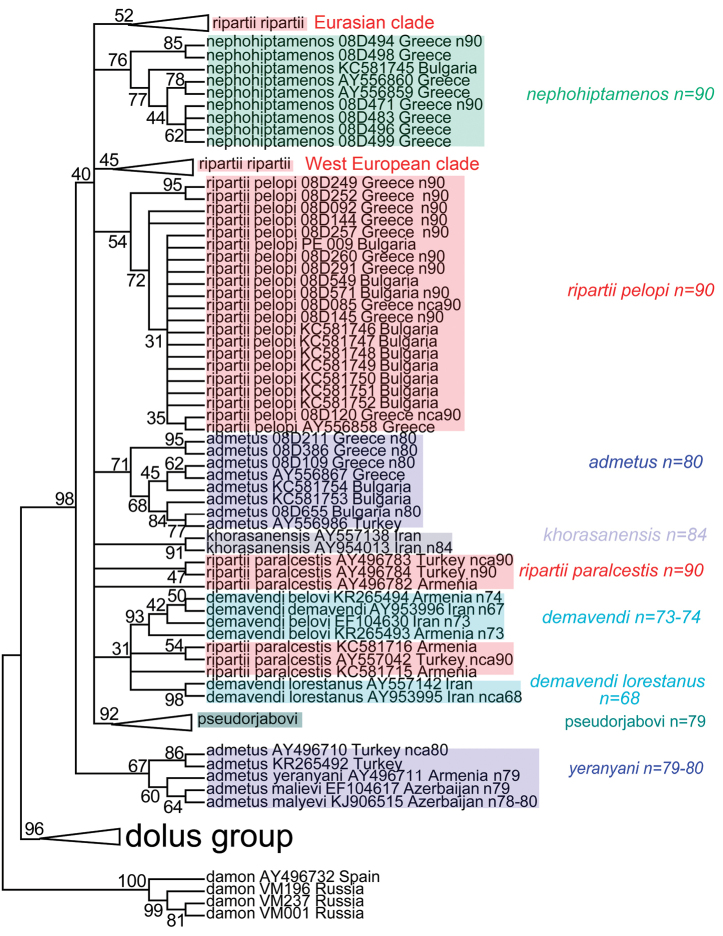
Fragment of the ML tree of *Polyommatus admetus* and *Polyommatus dolus* complexes based on analysis of *COI* barcodes and focused on *Polyommatus nephohiptamenos*, *Polyommatus admetus* and *Polyommatus ripartii pelopi*. *Polyommatus pseudorjabovi* clade is not shown in details, for its composition see Lukhtanov et al. (2015a). Details of the West-European and the “mixed” (Eurasian) clades of *Polyommatus ripartii* are shown in Fig. 19. Details of the *Polyommatus dolus* group are shown in Fig. 20. Numbers at nodes indicate bootstrap support.

**Figure 21.**
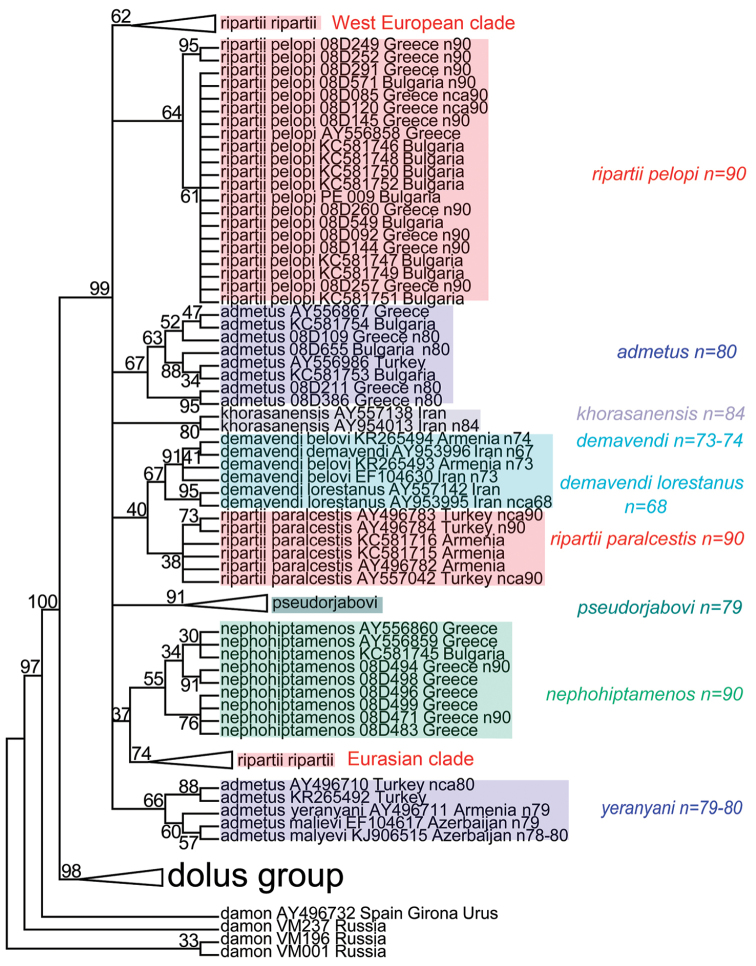
Fragment of the MP tree of *Polyommatus admetus* and *Polyommatus dolus* complexes based on analysis of *COI* barcodes and focused on *Polyommatus nephohiptamenos*, *Polyommatus admetus* and *Polyommatus ripartii pelopi*. *Polyommatus pseudorjabovi* clade is not shown in details, for its composition see Lukhtanov et al. (2015a). The West-European and the “mixed” (Eurasian) clades of *Polyommatus ripartii* are shown in Fig. 22. *Polyommatus dolus* group is shown in Fig. 23. Numbers at nodes indicate bootstrap support.

(Fig. 17a–g). Forewing length 15.8–17.5 mm.Upperside: ground color as in males, but lighter dark brown and without sex brand and scaletuft. Fringe greyish brown. Underside: ground color and general design as in males but fringes lighter-colored. Greenish blue basal suffusion almost invisible. White streak on hindwing underside is present in all paratypes and demonstrates a variable level of reduction.

#### Diagnosis.

*Polyommatus timfristos* (n=38) differs by at least three fixed chromosome fusions/fissions from the most closely related and allopatric *Polyommatus orphicus orphicus* and *Polyommatus orphicus eleniae* (n=41-42). *Polyommatus timfristos* (n=38) differs by at least nine fixed chromosome fusions/fissions from allopatric *Polyommatus aroaniensis* (n=47). From the closely related *Polyommatus orphicus* and *Polyommatus aroaniensis*, *Polyommatus timfristos* differs also by a number of nucleotide substitutions within the studied 657-bp fragment of the mitochondrial *COI* gene.

The chromosome number in *Polyommatus timfristos* (n=38) is similar (but not identical) to that found in *Polyommatus humedasae* (n=39, Vila et al. 2010). However, we are not sure that these karyotypes are related in their origin because they are not found in proximity and separated by an area where *Polyommatus orphicus* with n=41-42 is distributed. With respect to *COI* barcodes, the pair *Polyommatus timfristos*/*Polyommatus humedasae* is more differentiated than pairs *Polyommatus timfristos*/*Polyommatus aroaniensis* and *Polyommatus timfristos*/*Polyommatus orphicus*.

From sympatric and syntopic *Polyommatus ripartii pelopi* the new species can usually be distinguished by the absence of submarginal marking and strong reduction of greenish blue basal suffusion. These characteristics are usually (but not always) better expressed in *Polyommatus ripartii pelopi* specimens. In doubtful cases, the separation is only possible on the base of chromosomal and molecular markers since these species are different: the chromosome number of *Polyommatus ripartii pelopi* is n=90; they also have fixed differences in 33 positions within the studied 657-bp fragment of *COI* gene.

#### Ecology.

**Figure 32.**
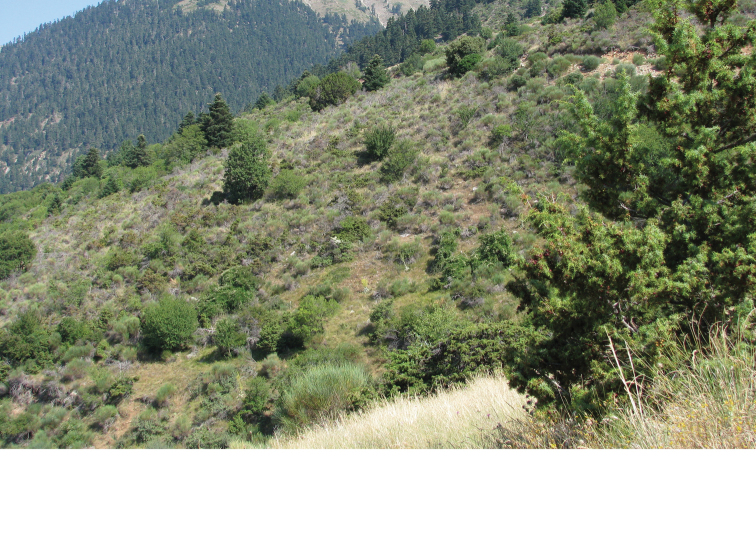
Habitat of *Polyommatus timfristos*. Central Greece, Mt. Timfristos, near Karpenisi, 1200 m, 20 July 2008. Photo by V.A. Lukhtanov.

**Figure 33.**
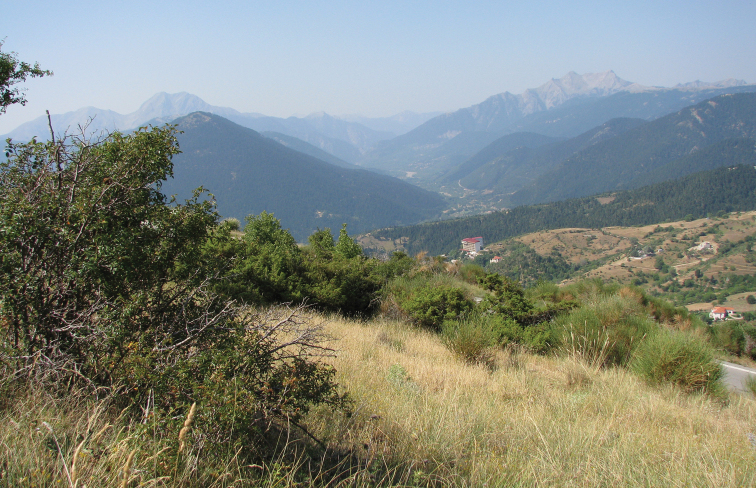
Habitat of *Polyommatus timfristos*. Central Greece, Mt. Timfristos, near Karpenisi, 1200 m, 20 July 2008. Photo by V.A. Lukhtanov.

**Figure 34.**
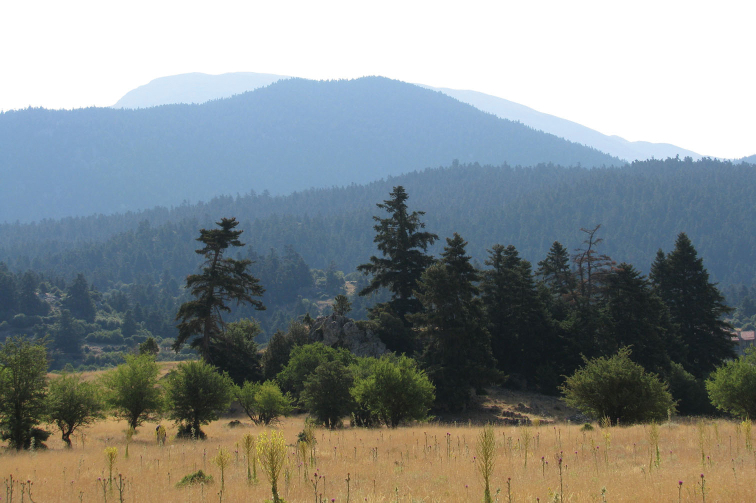
Habitat of *Polyommatus timfristos*. Central Greece, Mt. Parnassos, 19 July 2008. Photo by V.A. Lukhtanov.

**Figure 35.**
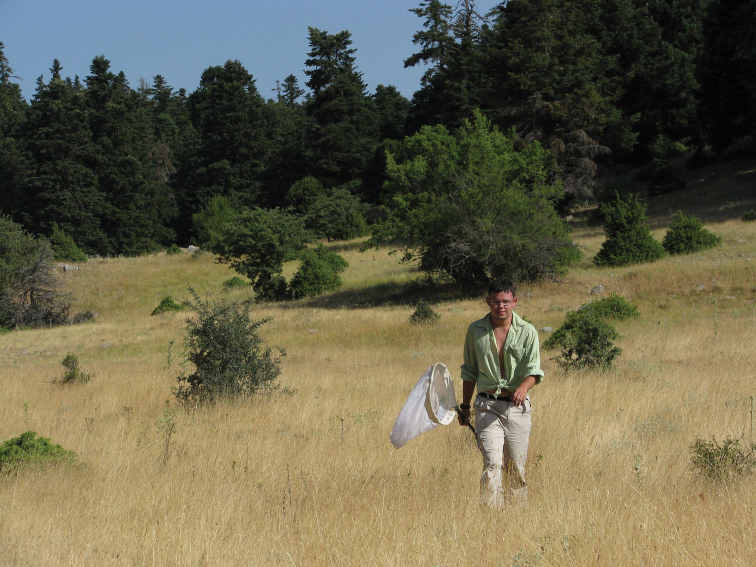
Habitat of *Polyommatus timfristos*. Central Greece, Mt. Parnassos, 19 July 2008. Photo by V.A. Lukhtanov.

*Polyommatus timfristos* sp. n. inhabits xerothermic and xeromontane localities and dry meadows from 1200 to 1800 m altitude (Figs 32–35). It was found in complete syntopy with *Polyommatus ripartii pelopi* and *Polyommatus admetus*.

#### Etymology.

Timfristos is a mountain in the eastern part of Evrytania and the western part of Phthiotis in Central Greece. The name is a noun.

## Distribution areas

### *Polyommatus admetus*

Figs 24–26

This species is widespread in the Balkan Peninsula. It is local in the northern part of Hungary (Ilonczai and Bálint 2001, Bálint et al. 2006) and recorded in Slovakia (Kulfan and Kulfan 1992, Eckweiler and Bozano 2016). It has been shown to be widely distributed in the western part of Romania, but no exact localities were provided (Eckweiler and Bozano 2016). It is common in Greece and found in Croatia, Bosnia and Herzegovina, Montenegro, Serbia, Bulgaria, The Republic of Macedonia, Albania and European Turkey (Sijaric and Mihljevic 1972, Hesselbarth et al. 1995, Abadjiev 2001, Tolman and Lewington 2008, Eckweiler and Bozano 2016).

### *Polyommatus ripartii*

Fig. 27

*Polyommatus ripartii* is widespread in the southern part of the Balkan Peninsula (Greece and Bulgaria); however, it is more local in the north. It is known from Albania, the Republic of Macedonia, south Serbia (Kudrna et al. 2011, Eckweiler and Bozano 2016), Bosnia and Herzegovina (Koren 2010). It was mentioned for European Turkey (Hesselbarth et al. 1995, Tolman and Lewington 2008) and recently found in Croatia (Koren 2010, Dincă et al. 2013a).

### *Polyommatus nephohiptamenos*

Figs 28–30

*Polyommatus nephohiptamenos* has a dot-like distribution area and is known from the high altitudes of north-east Greece (Mt Pangeon, Mt Phalakro and Mt Orvilos) and south-west Bulgaria (Mt Orvilos, also known as Mt Slavyanka, Mt Alibotush and Kitka Planina) (Kolev 1994, Tolman and Lewington 2008, Eckweiler and Bozano 2016).

### *Polyommatus aroaniensis*

Fig. 31

*Polyommatus aroaniensis* has been considered as a relatively widespread species (Kolev and van der Poorten 1997). Apart from its type-locality (South Greece, Peloponnese), it has been recorded in different parts of Central and Northern Greece (Brown 1976a, Wakeham-Dawson and Spurdens 1994, Wakeham-Dawson 1998, Pamperis 2009), from a few areas in south Macedonia (Kolev and van der Poorten 1997, Melovski and Bozhinovska 2014) and from some localities in south-west Bulgaria and one isolated place in the central part of the country (Abadjiev 2001, Kolev 1994, Kolev and van der Poorten 1997). Tshikolovets (2011) and Eckweiler and Bozano (2016) show its distribution extending into Albania, although the species has not been recorded from this country in recent surveys (Verovnik and Popović 2013). Verovnik et al. (2015) recorded it in Bosnia and Herzegovina. Koren and Laus (2015) recorded it in Croatia; however, this record was not confirmed by molecular data (Lovrenčić et al. 2016).

Our chromosomal data confirm *Polyommatus aroaniensis* in South Greece (Peloponnese), but cannot confirm it in Central and Northern Greece and in Bulgaria where it is replaced by the closely related allopatric species *Polyommatus timfritos* and *Polyommatus orphicus*. In the light of the data obtained, the occurrence of *Polyommatus aroaniensis* in Bulgaria, Albania, Macedonia and Bosnia and Herzegovina seems to be doubtful and requires a confirmation based on chromosomal analysis. We cannot exclude that the populations from Albania, Macedonia and Bosnia and Herzegovina could represent *Polyommatus orphicus* or even undescribed taxa of the subgenus Agrodiaetus.

### *Polyommatus timfristos*

Figs 32–35

This species is known from Timfristos and Parnassos Mts in Central Greece only.

### *Polyommatus orphicus*

Figs 36–39

This species is known from South Bulgaria and Northern Greece only. However, its occurrence in other countries in the northern Balkan is theoretically possible (see above).

## An alternative classification and conservation

Theoretically, the main groupings in the *Polyommatus humedasae* – *Polyommatus orphicus* – *Polyommatus timfristos* – *Polyommatus aroaniesnsis* subcomplex can be interpreted as subspecies-level taxa, if the polytypic species concept is applied. None of them appears to be sympatric in distribution, and taken together they form a moderately supported monophyletic lineage on the *COI*+*ITS2* tree (Fig. 9). Under this scenario, this subcomplex would be considered a diverse array of allopatric populations, each of which possesses unique genetic attributes (karyotypes and molecular markers) and is distributed in a particular area within the Alp-Balkan region. As possible theoretical support for this alternative classification, one can argue that differences in chromosome numbers in *Agrodiaetus* do not necessarily result in complete reproductive isolation, and, at least in some particular cases, do not prevent interspecific hybridization and genetic introgression (Lukhtanov et al. 2015b).

However, even if the last statement is true, it does not mean that chromosomal rearrangements are irrelevant to formation of genetic barriers between populations. Chromosome changes have been shown to be important in speciation in the blue butterflies (Lukhtanov et al. 2005, 2015b, Kandul et al. 2007, Talavera et al. 2013b). Even a weak decrease in fertility in heterozygotes for multiple chromosomal rearrangenments can result in selection against them and in formation of a boundary between chromosomally diverged homozygous populations. Additional studies are required to shed light on this topic. Recent studies have treated *Polyommatus humedasae*, *Polyommatus aroaniensis* and *Polyommatus orphicus* as species-level taxa (Eckweiler and Bozano 2016), which our study suggests is a reasonable interpretation although distribution areas of *Polyommatus aroaniensis* and *Polyommatus orphicus* should be corrected. Based on our current knowledge, if *Polyommatus humedasae*, *Polyommatus aroaniensis* and *Polyommatus orphicus* are considered species-level taxa, *Polyommatus timfristos* should be treated as a species-level taxon as well.

Regardless of its taxonomic status as a species or subspecies, *Polyommatus timfristos* represents a unique entity within the genus *Polyommatus* that deserves additional study. A better understanding of its evolutionary history may be helpful in understanding mechanisms of chromosomal diversification within the genus, and may further elucidate the biogegraphy of the south Balkan and Aegean regions. As a distinct taxonomic entity occupying a very restricted area in Central Greece it should be considered a candidate on the list of protected species in Greece and the whole of Europe.

## Biogeography

Analysis of distribution areas and phylogeny of the *Polyommatus dolus* lineage shows that the phylogeograpic history of this complex involved a combination of dispersal and vicariance events with a clear general trend of dispersal from the East (Iran), where the group most likely arose, to the West: to the Mediterranean area and to the Iberian Peninsula (Vila et al. 2010). The Europe was estimated to be colonized approximately 1.24 Mya (range 0.88–1.64 Mya). Approximately 1.15 Mya (range 0.80–1.51 Mya), the European lineage was divided into three subclades located (1) in the Balkan Mountains and Alps (*Polyommatus aroaniensis* sensu auctorum: the Balkans; *Polyommatus humedasae*: the Alps), (2) southern Spain (*Polyommatus violetae*), and (3) the Iberian-Italian region (*Polyommatus fabressei* + *Polyommatus dolus*), respectively (Vila et al. 2010).

Three chromosomal sublineages discovered in our study (*Polyommatus aroaniensis* sensu stricto + *Polyommatus orphicus* +*Polyommatus timfristos*) represent late Pleistocene splits of the Balkan subclade that evolved in allopatry within the Balkan refugium. Given the deep level of chromosomal divergence between these sublineages, we assume that there was a long period of allopatric differentiation when they were separated by geographic or/and ecological barriers. In our opinion, this is evidence for presence of three separate Balkan subrefugia in the past (Pelonnese, Central Greece and Northern Grecee/South Bulgaria).

Greece, as a part of the Balkan Peninsula, has been already reported to harbor genetically differentiated lineages from the rest of the Balkans for a number of animal species as a result of evolution in multiple separate refugia (Kasapidis et al. 2005, Alexandri et al. 2012, Karaiskou et al. 2014). Thus, our data provide a chromosomal evidence for this refugia-within-refugia concept (Gòmez and Lunt 2007, Karaiskou et al. 2014), and the discovery of a new, chromosomally diverged species *Polyommatus timfristos* stresses the biogeographic importance of Central Greece as a separate Pleistocene refugium within the Balkans.

## Taxonomic conclusion

We propose the following taxonomic arrangement of the *Polyommatus dolus* and *Polyommatus admetus* lineages (chromosome numbers are in parentheses when known, **the Balkan taxa are in bold**):

*Polyommatus dolus* lineage

*Polyommatus dolus* (Hübner, [1823])

*Polyommatus dolus dolus* (Hübner, [1823]) (n=123-125)

*Polyommatus dolus vittatus* (Oberthür, 1892) (n=124-125)

*Polyommatus dolus virgilia* (Oberthür, 1910) (n=122)

*Polyommatus dolus gargano* (Wimmers, 1931) (n=122)

*Polyommatus dolus paravirgilia* Verity, 1943 (n unknown)

*Polyommatus fulgens* (Sagarra, 1925)

*Polyommatus fulgens fulgens* (Sagarra, 1925) (n=109)

*Polyommatus fulgens ainsae* (Forster, 1961) (n=108-110)

*Polyommatus fulgens pseudovirgilia* (de Lesse, 1962) (n=108)

*Polyommatus fulgens leonensis* (Verhulst, 2004) (n unknown)

*Polyommatus menalcas* (Freyer, [1837]) (n=85)

*Polyommatus fabressei* (Oberthür, 1910) (n=90)

*Polyommatus violetae* (Gómez-Bustillo, Expósito & Martínez, 1979)

*Polyommatus violetae violetae* (Gómez-Bustillo, Expósito & Martínez, 1979) (n=90)

*Polyommatus violetae subbaeticus* (Gil-T. & Gil-Uceda, 2005) (n=90)

*Polyommatus humedasae* (Toso & Balletto, 1976) (n=39)

***Polyommatus orphicus* Kolev, 2005**

***Polyommatus orphicus orphicus* Kolev, 2005 (n=41-42)**

***Polyommatus orphicus eleniae* Coutsis & De Prins, 2005 (n=41-42)**

***Polyommatus timfristos* Lukhtanov, Vishnevskaya & Shapoval, sp. n. (n=38)**

***Polyommatus aroaniensis* (Brown, 1976) (n=47)**

*Polyommatus alcestis* (Zerny, 1932) (n=20-21)

*Polyommatus karacetinae* (Lukhtanov & Dantchenko, 2002)

*Polyommatus karacetinae karacetinae* (Lukhtanov & Dantchenko, 2002) (n=19)

*Polyommatus karacetinae urmiaensis* Schurian & Ten Hagen, 2003, **comb. n.** (n=19)

*Polyommatus dantchenkoi* (Lukhtanov & Wiemers, 2003) (n=40-42)

*Polyommatus eriwanensis* (Forster, 1960) (n=32-34)

*Polyommatus interjectus* (de Lesse, 1960) (n=29-32)

*Polyommatus rjabovianus* (Koҫak, 1980) (= *rjabovi* (Forster, 1960)

*Polyommatus rjabovianus rjabovianus* (Koҫak, 1980) (n=49)

*Polyommatus rjabovianus masul* Lukhtanov, Dantchenko, Vishnevskaya & Saifitdinova, 2015 (n=43)

*Polyommatus valiabadi* (Rose & Schurian, 1977) (n=24)

*Polyommatus admetus* lineage

*Polyommatus ripartii* (Freyer, 1830)

*Polyommatus ripartii ripartii* (Freyer, 1830) (= *agenjoi* Forster, 1965; = *budashkini* Kolev & de Prins, 1995; = *exuberans* Verity, 1926; = *montanesa* Gómez-Bustillo, 1971; = *mozuelica* Agenjo, 1973; = *ovchinnikovi* Lukhtanov & Dantchenko, 2002; = *ramonagenjo* Koçak & Kemal, 2001; = *rippertii* Boisduval, 1832; = *sarkani* Lukhtanov & Dantchenko, 2002; = *susae* Bertaccini, 2003) (n=90)

***Polyommatus ripartii pelopi* (Brown, 1976) (n=90)**

*Polyommatus ripartii paralcestis* (Forster, 1960) (n=90)

*Polyommatus ripartii colemani* (Lukhtanov & Dantchenko, 2002) (n=90)

*Polyommatus ripartii tengritaghicus* Koҫak & Kemal, 2001 (n unknown)

***Polyommatus nephohiptamenos* (Brown & Coutsis, 1978) (n=90)**

*Polyommatus demavendi* (Pfeiffer, 1938) (n=67-74)

*Polyommatus demavendi demavendi* (Pfeiffer, 1938)

*Polyommatus demavendi amasyensis* (de Lesse, 1961)

*Polyommatus demavendi belovi* (Dantchenko & Lukhtanov, 2005)

*Polyommatus demavendi ahmadi* (Carbonell, 2001)

*Polyommatus demavendi lorestanus* Eckweiler, 1997

*Polyommatus khorasanensis* (Carbonell, 2001) (n=74)

*Polyommatus pseudorjabovi* Lukhtanov, Dantchenko, Vishnevskaya & Saifitdinova, 2015 (n=79)

***Polyommatus admetus* (Esper, [1783])** (= *anatoliensis* Forster, 1960) (n=80)

*Polyommatus yeranyani* (Dantchenko & Lukhtanov, 2005), **stat. n.**

*Polyommatus yeranyani yeranyani* (Dantchenko & Lukhtanov, 2005) (n=78-80)

*Polyommatus yeranyani malievi* (Dantchenko & Lukhtanov, 2005), **comb. n.** (n=78-80)

## Supplementary Material

XML Treatment for Polyommatus (Agrodiaetus) timfristos

## Figures and Tables

**Figure 19.**
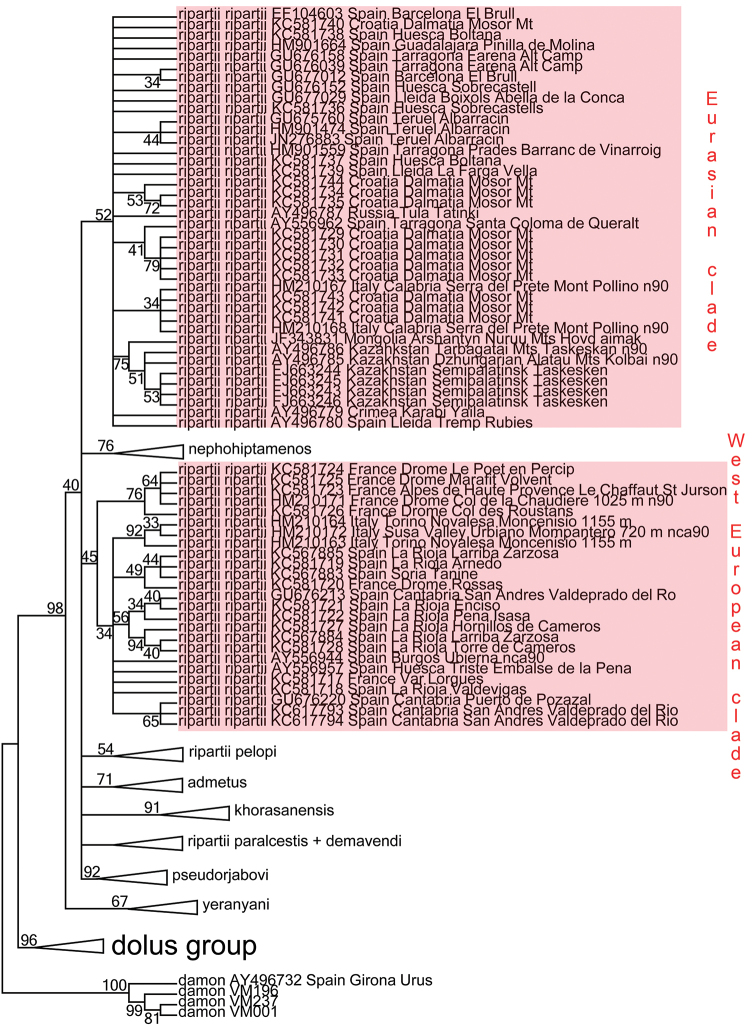
Fragment of the ML tree of *Polyommatus admetus* and *Polyommatus dolus* complexes based on analysis of *COI* barcodes and focused on details in the structure of the West-European and the “mixed” (Eurasian) clades of *Polyommatus ripartii*. Numbers at nodes indicate bootstrap support.

**Figure 20.**
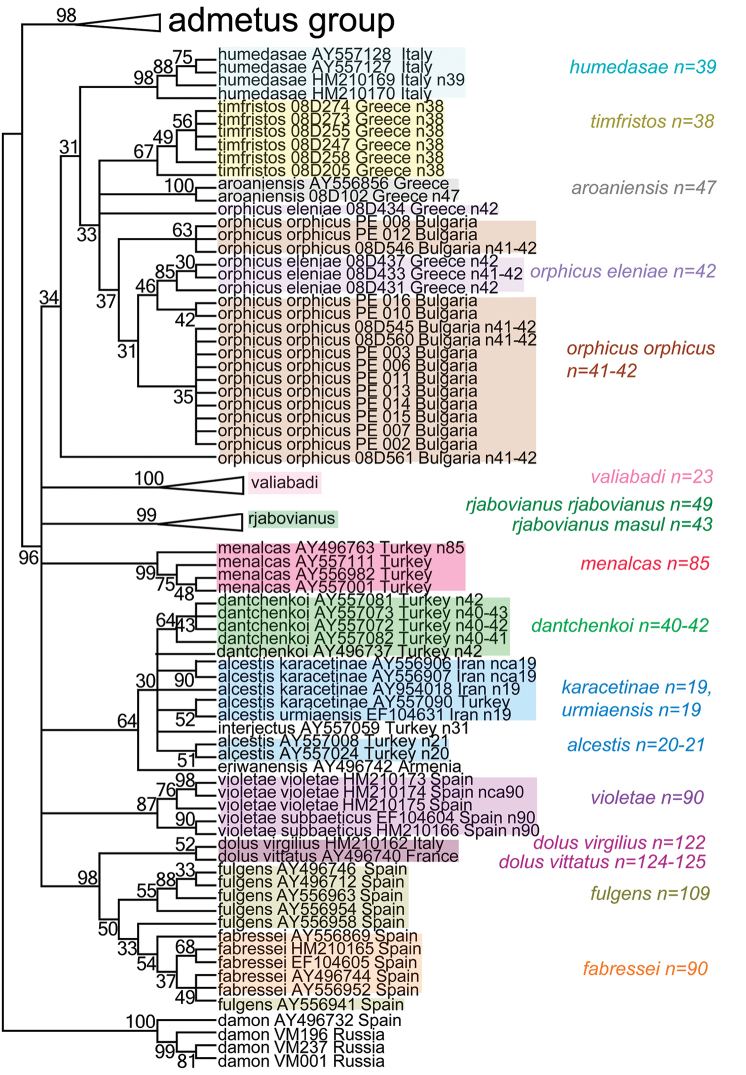
Fragment of the ML tree based on analysis of *COI* barcodes and focused on details of the *Polyommatus dolus* group. *Polyommatus rjabovianus* and *Polyommatus valiabadi* clades are not shown in details, for their composition see Lukhtanov et al. (2015a). Numbers at nodes indicate bootstrap support.

**Figure 22.**
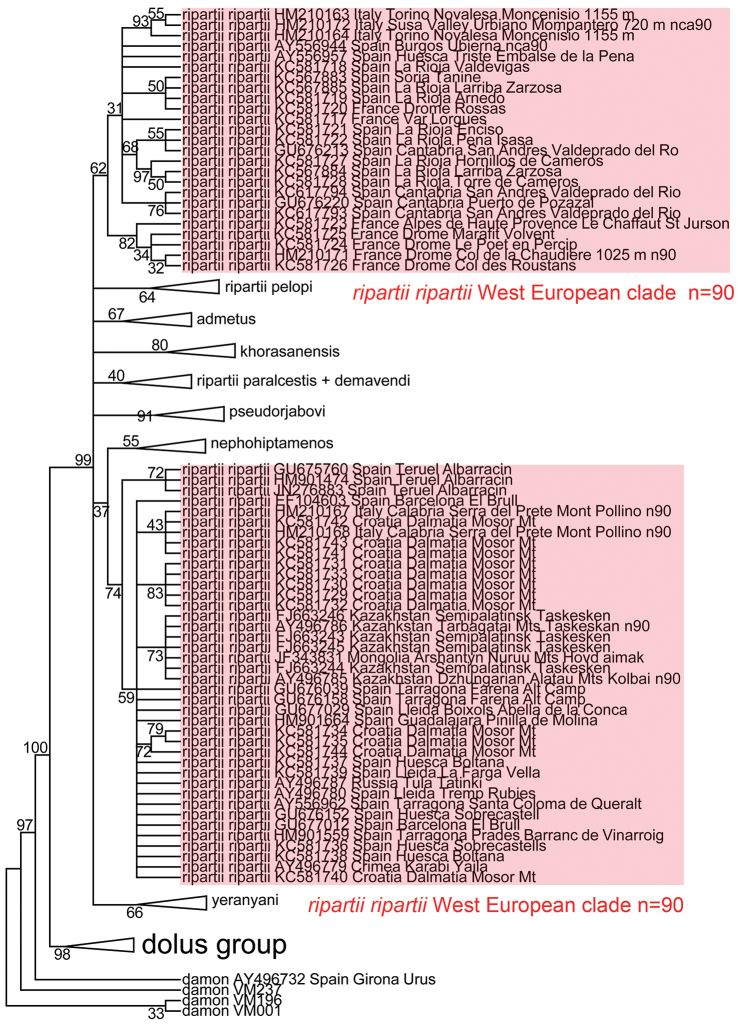
Fragment of the MP tree of *Polyommatus admetus* and *Polyommatus dolus* complexes based on analysis of *COI* barcodes and focused on details of the West-European and the “mixed” (Eurasian) clades of *Polyommatus ripartii*. Numbers at nodes indicate bootstrap support.

**Figure 23.**
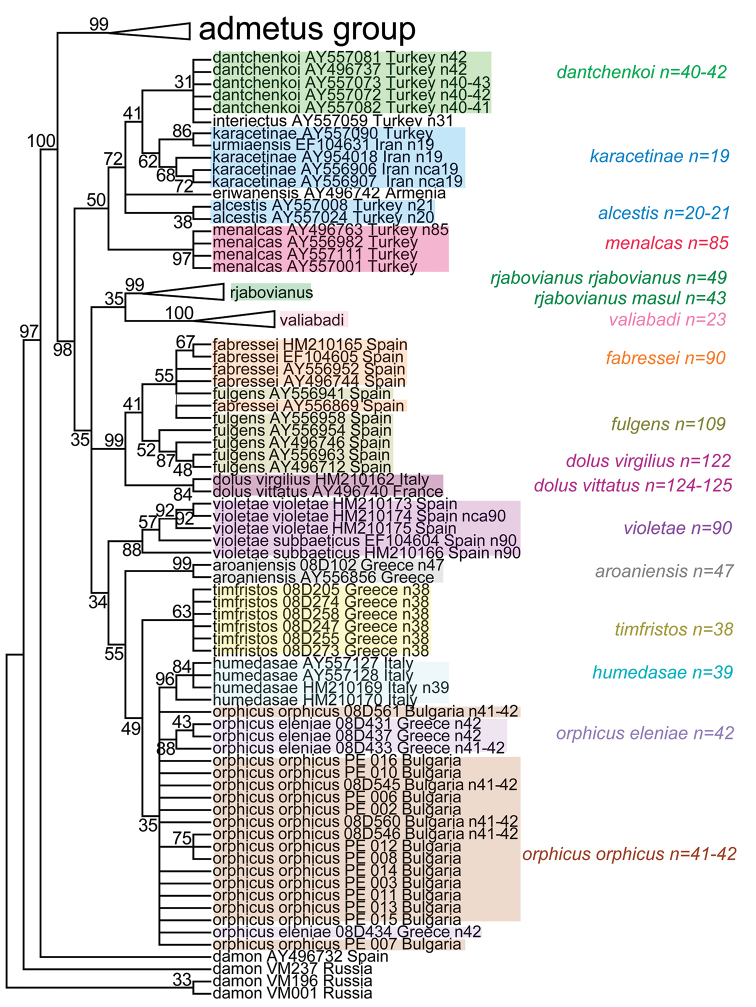
Fragment of the MP tree based on analysis of *COI* barcodes and focused on details of the *Polyommatus dolus* group. *Polyommatus rjabovianus* and *Polyommatus valiabadi* clades are not shown in details, for their composition see Lukhtanov et al. (2015a). Numbers at nodes indicate bootstrap support.

**Figure 24.**
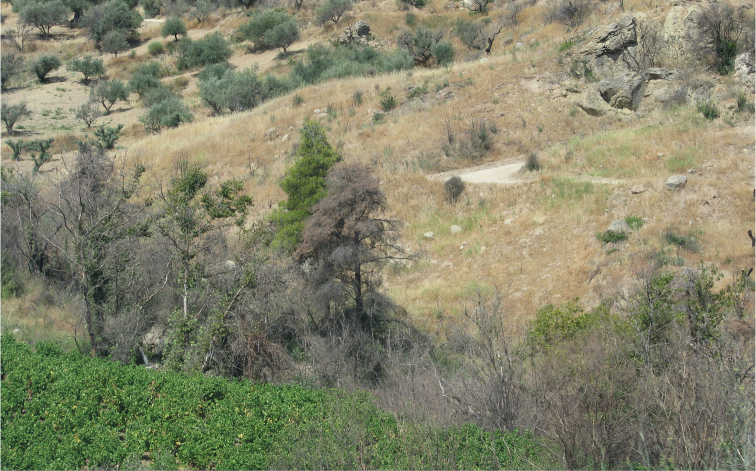
Habitat of *Polyommatus admetus*. Greece, Peloponnesse Peninsula, Mt. Chelmos, near Kalavrita, 800 m, 16 july 2008. Photo by V.A. Lukhtanov.

**Figure 25.**
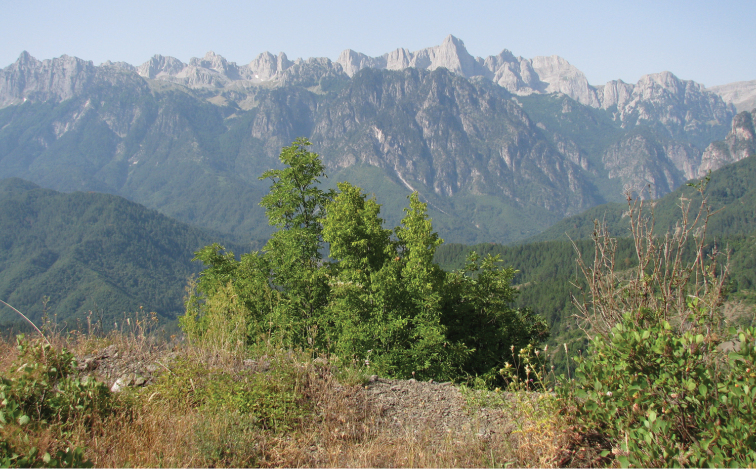
Habitat of *Polyommatus admetus*. Greece, Smolikas Mt, Konitsa, 950 m, 22 July 2008. Photo by V.A. Lukhtanov.

**Figure 26.**
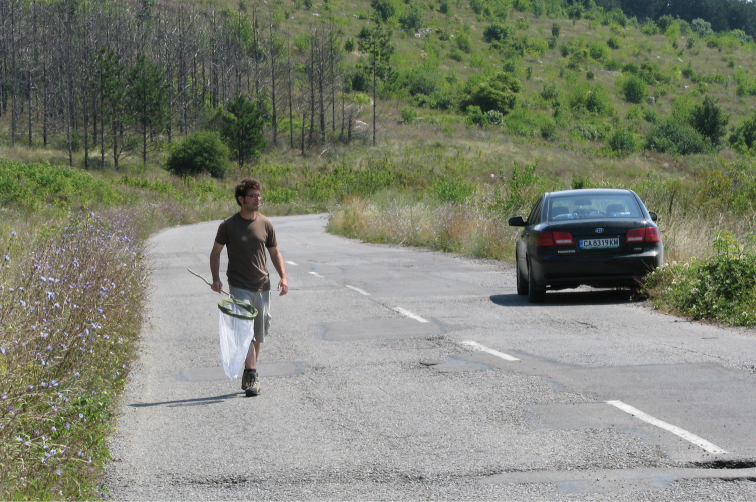
Habitat of *Polyommatus admetus*. W Bulgaria, near Dragoman, 700 m, 29 July 2008. Photo by V.A. Lukhtanov.

**Figure 27.**
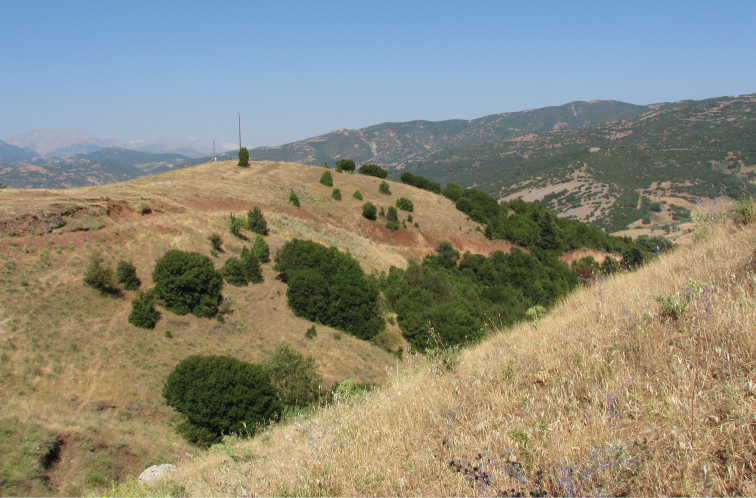
Habitat of *Polyommatus ripartii pelopi*. Greece, Peloponnesse Peninsula, Mt. Chelmos, near Kalavrita, 800 m, 17 July 2008. Photo by V.A. Lukhtanov.

**Figure 28.**
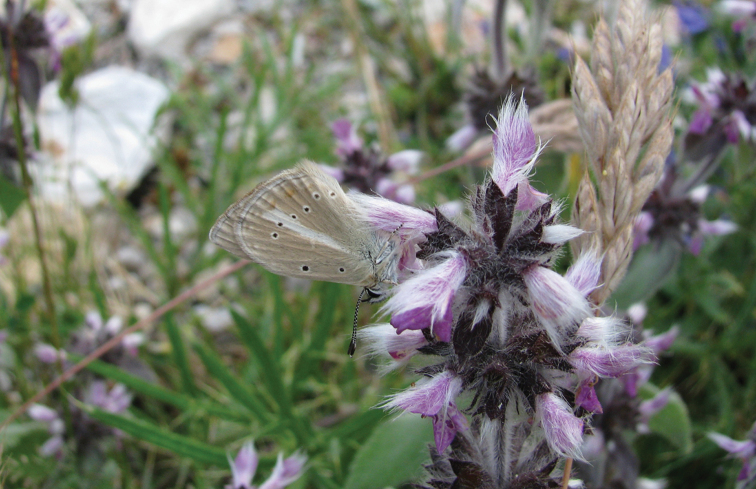
*Polyommatus nephohiptamenos*. Northern Greece, Falakro Mt, near Granitis, 1700 m, 23 July 2008. Photo by V.A. Lukhtanov.

**Figure 29.**
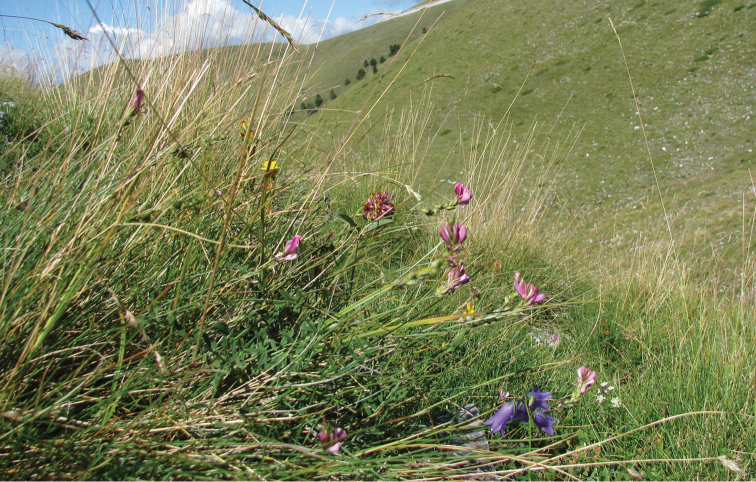
Habitat of *Polyommatus nephohiptamenos*. Northern Greece, Falakro Mt, near Granitis, 1700 m, 24 July 2008. Photo by V.A. Lukhtanov.

**Figure 30.**
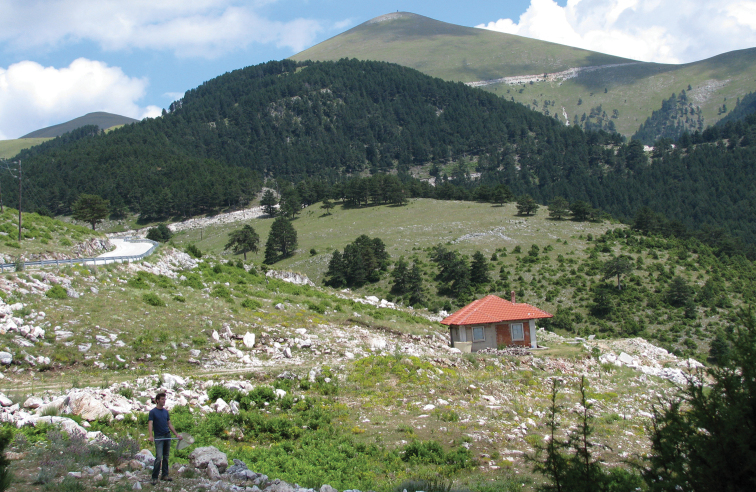
Habitat of *Polyommatus nephohiptamenos*. Northern Greece, Falakro Mt, near Granitis, 1700 m, 24 July 2008. Photo by V.A. Lukhtanov.

**Figure 31.**
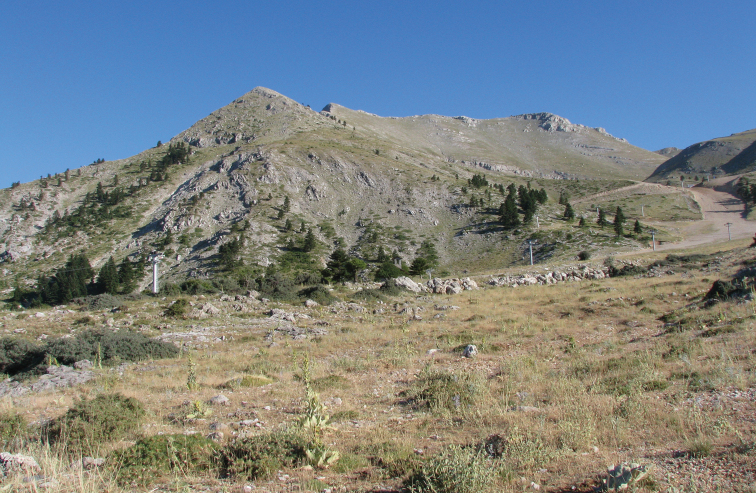
Habitat of *Polyommatus aroaniensis* in its type locality. Greece, Peloponnesse Peninsula, Mt. Chelmos, near Kalavrita, 1600 m, 16 July 2008. Photo by V.A. Lukhtanov.

**Figure 36.**
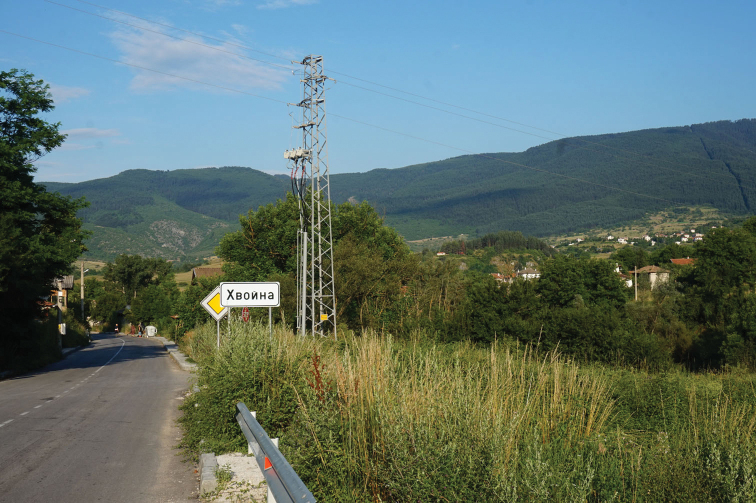
Hvoyna, Bulgaria, type locality of *Polyommatus orphicus orphicus*, 2 July 2016. Photo by E. Pazhenkova.

**Figure 37.**
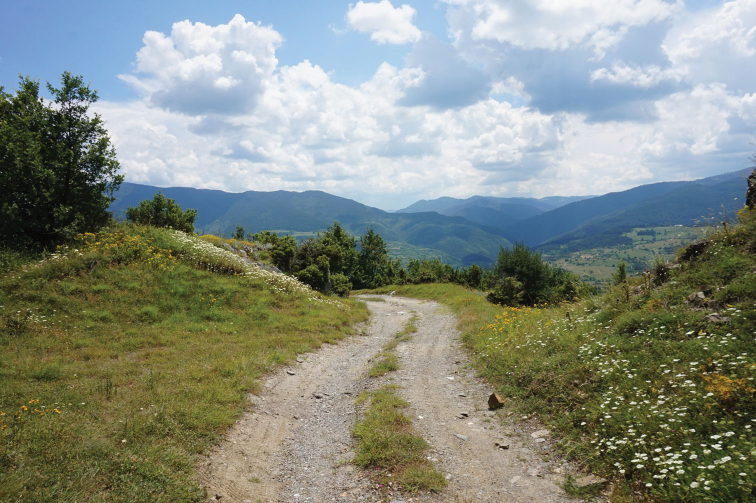
Habitat of *Polyommatus orphicus orphicus* in its type locality. Bulgaria, Hvoyna, 3 July 2016. Photo by E. Pazhenkova.

**Figure 38.**
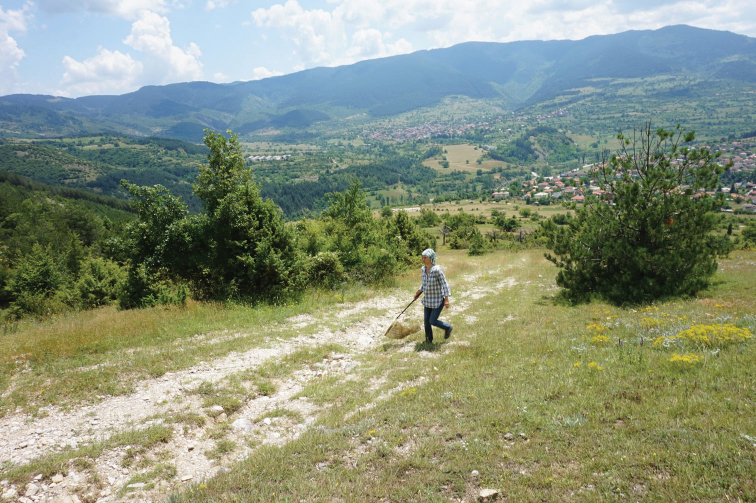
Habitat of *Polyommatus orphicus orphicus*. Bulgaria, Hvoyna, 3 July 2016. Photo by I. Zubkova.

**Figure 39.**
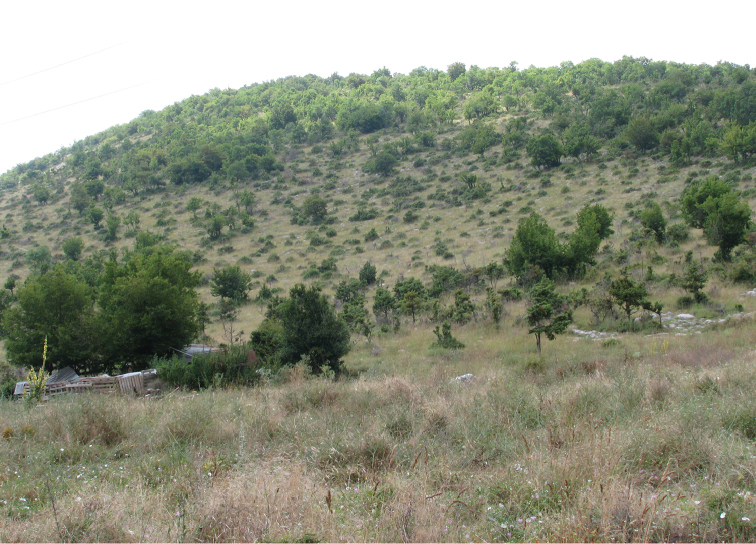
Habitat of *Polyommatus orphicus eleniae* in its type locality. Northern Greece, Makedonía, Dráma district, near Granítis, 900 m, 23 July 2008. Photo by V.A. Lukhtanov.

## Appendix 1

Alphabetical list of the nominal taxa described within the Polyommatus (Agrodiaetus) admetus and Polyommatus (Agrodiaetus) dolus complexes.

***admetus* (Esper, [1783])**

Original combination: *P.*[*apilio*] *Pl.*[*ebeius*] *Rur.*[*alis*] *Argus Admetus*

In: Esper EJC (1777-1807) Die Schmetterlinge in Abbildungen nach der Natur mit Beschreibungen (I)2: 148; Tab. LXXXII, figs 2, 3.

Type locality: “Ungarn ... bis nach Semlin an die Gränze von Sclavonien” [Hungary].

Syntypes in Naturwissenschaftliche Sammlung, Wiesbaden, Germany, and in Zoologische Staatssammlung, Munich, Germany (after Häuser and Eckweiler 1997).

Current status: species.

***ahmadi* (Carbonell, 2001)**

Original combination: *Agrodiaetus ahmadi*

In: Carbonell F (2001) Contribution à la connaissance du genre *Agrodiaetus* Hübner (1822), *Agrodiaetus ahmadi* et *Agrodiaetus khorasanensis* nouvelles espèces dans le Nord de l‘Iran (Lepidoptera, Lycaenidae). Linneana Belgica 18: 105–110.

Type locality: environs d’Alulak, 1600 m., E. Prov. De Zanjan, N. Iran [N Iran, Alilak, Qazvin].

Holotype in Muséum National d’Histoire Naturelle, Paris.

Current status: subspecies of Polyommatus (Agrodiaetus) demavendi (after Eckweiler and Bozano 2016).

***agenjoi* (Forster, 1965)**

Original combination: *Agrodiaetus admetus agenjoi*

In: Forster W (1965) *Agrodiaetus admetus agenjoi* ssp. n. Entomologische Zeitschrift 75(18): 198.

Type locality: “Barcelona, Taradell” [Spain: Barcelona].

Holotype in Museo Nacional de Ciencias Naturales, Madrid, Spain (after Häuser and Eckweiler 1997).

Current status: preoccupied name (see *ramonagenjoi*).

***ainsae* (Forster, 1961)**

Original combination: *Agrodiaetus dolus ainsae*

In: Forster W (1961) Bausteine zur Kenntnis der Gattung *Agrodiaetus* Scudd. (Lep. Lycaen.) II. Zeitschrift der Wiener Entomologischen Gesellschaft 46: 76. Taf. 14, 15, figs 5, 6.

Type locality: “Spanien, Pyrenäen, Ainsa” [Spain: Huesca].

Holotype in Naturhistorisches Museum, Wien, Austria.

Current status: subspecies of Polyommatus (Agrodiaetus) fulgens (after Eckweiler and Bozano 2016).

***alcestis* (Zerny, 1932)**

Original combination: Lycaena (Hirsutina) ripperti alcestis

In: Zerny H (1932) Lepidopteren aus dem nördlichen Libanon. Deutsche Entomologische Zeitschrift Iris 46(4): 186.

Type locality: Becharré [Lebanon].

Lectotype in The Natural History Museum, London (after Bálint 1999: 9).

Current status: species.

***amasina* (Neuburger, 1900)**

Original combination: Lycaena menalcas ab. amasina

In: Neuburger W (1900) *Lycaena menalcas* Frr. ♂ aberr. (Lep.). Illustrierte Zeitschrift für Entomologie 5: 370.

Type locality: “Aus der Gegend von Amasia” [Turkey: Amasya].

Holotype in coll. W. Neuburger [?] (after Häuser and Eckweiler 1997).

Current status: unavailable (infrasubspecific name).

***amasyensis* (de Lesse, 1961)**

Original combination: *Agrodiaetus demavendi amasyensis*

In: de Lesse H (1961) Variations géographiques des caractères externes chez les espèces autrefois réunies sous le nom d’*Agrodiaetus ripartii* Freyer (Lep. Lycaenidae). Revue Française d’Entomologie 28(2): 96.

Type locality: “Amasya” [Turkey: Amasya].

Holotype in Museum National d’Histoire Naturelle, Paris, France (after Häuser and Eckweiler 1997).

Current status: subspecies of Polyommatus (Agrodiaetus) demavendi (after Eckweiler and Bozano 2016).

***anatoliensis* (Forster, 1960)**

Original combination: *Agrodiaetus admetus anatoliensis*

In: Forster W (1960) Einige neue Formen der Gattung *Agrodiaetus* Scudd. (Lep. Lycaen.). Entomologische Zeitschrift 70(3): 20.

Type locality: “Akshehir” [Turkey: Akşehir].

Holotype in Zoologische Staatssammlung, München, Germany (after Häuser and Eckweiler 1997).

Current status: synonym of Polyommatus (Agrodiaetus) admetus admetus (after Eckweiler and Bozano 2016).

***anticodiscoelongata* (Verity, 1943)**

Original combination: *Agrodiaetus dolus anticodiscoelongata*

In: Verity R (1943) Farfalle diurne d’Italia 2: 323.

Type locality: “Monti Sibillini” [Central Italy].

Current status: synonym of Polyommatus (Agrodiaetus) dolus virgilius (after Eckweiler and Bozano 2016).

***aroaniensis* (Brown, 1976)**

Original combination: *Agrodiaetus alcestis aroaniensis*

In: Brown J (1976) Notes regarding previously undescribed European taxa of the genera *Agrodiaetus* Hübner, 1822 and *Polyommatus* Klug, 1801 (Lep., Lycaenidae). Entomologist’s Gazette 27(2): 78.

Type locality: “Kamena Allonia, Mt. Chelmos” [Greece: Peloponnese].

Holotype in coll. J. Brown, Sutton (after Häuser and Eckweiler 1997).

Current status: species.

***belovi* (Dantchenko & Lukhtanov, 2005)**

Original combination: *Agrodiaetus belovi*

In: Dantchenko A, Lukhtanov V (2005) New taxa of the brown species-complex of the genus *Agrodiaetus* Hübner, (1822) from Transcaucasia (Lepidoptera, Lycaenidae). Atalanta 35: 327–334, 472–475.

Type locality: Armenia, Gegamsky mts.

Holotype in Museum of Comparative Zoology (Harvard University, Cambridge, MA, USA).

Current status: subspecies of Polyommatus (Agrodiaetus) demavendi (after Eckweiler and Bozano 2016).

***budashkini* (Kolev & De Prins, 1995)**

Original combination: Polyommatus (Agrodiaetus) budashkini

In: Kolev Z, De Prins W (1995) A new species of the “brown *Agrodiaetus*” complex from the Crimea (Lepidoptera: Lycaenidae). Phegea 23(2): 121.

Type locality: “Crimea, Sudak” [Ukraine: Crimea].

Holotype in Vlaamse Lepidoptera Collectie, Antwerpen.

Current status: synonym of Polyommatus (Agrodiaetus) ripartii ripartii.

***colemani* (Lukhtanov & Dantchenko, 2002)**

Original combination: *Agrodiaetus ripartii colemani*

In: Lukhtanov V, Dantchenko A (2002) Descriptions of new taxa of the genus *Agrodiaetus* Hübner, [1822] based on the karyotype investigation (Lepidoptera, Lycaenidae). Atalanta 33(1/2): 81–107.

Type locality: Kazakhstan, Shymkentskaya oblast’, Ugamskiy Khrebet, Saryaigyr, 1600 m.

Holotype in Museum of Comparative Zoology, Harvard University, Cambridge, MA, USA.

Current status: subspecies of Polyommatus (Agrodiaetus) ripartii.

***crassipuncta* (Stauder, 1921)**

Original combination: Lycaena dolus virgilia Obth. f. n. crassipuncta

In: Stauder (1921) Deutsche Entomologische Zeitschrift [Iris] 35: 31.

Type locality: “Unteritalien” [South Italy].

Current status: synonym of Polyommatus (Agrodiaetus) dolus virgilius (after Eckweiler and Bozano 2016).

***dantchenkoi* (Lukhtanov & Wiemers, 2003)**

Original combination: *Agrodiaetus dantchenkoi*

In: Lukhtanov V, Wiemers M, Meusman K (2003) Description of a new species of the “brown” *Agrodiaetus* complex from South-East Turkey (Lycaenidae). Nota lepidopterologica 26(1/2): 65–71.

Type locality: Turkey, Prov. Van, 34 km N Catak [SE Turkey (Van)].

Holotype in Museum of Comparative Zoology, Harvard University, Cambridge, MA, USA.

Current status: species.

***demavendi* (Pfeiffer, 1938)**

Original combination: Lycaena ripertii Frr. ssp. n. demavendi m.

In: Pfeiffer E (1938) Notizen über persische Lycaenidae (Lepid.). Mitteilungen der Münchner Entomologischen Gesellschaft 28(2): 194.

Type locality: Ort Demavend (Tar-Tal) 2200-2500 m [Iran: Elburs mts.].

Holotype in Zoologische Staatssammlung, München, Germany (after Häuser and Eckweiler 1997).

Current status: species.

***discoelongata* (Courvoisier, 1914)**

Original combination: *Lycaens dolus disco-elongata*

In: Courvoisier (1914) Deutsche Entomologische Zeitschrift [Iris] 28: 186.

Type locality: not stated.

Current status: synonym of Polyommatus (Agrodiaetus) dolus (after Eckweiler and Bozano 2016).

***dolus* (Hübner, [1823])**

Original combination: *Papilio dolus*

In: Hübner J (1796-[1838]) Sammlung europäischer Schmetterlinge I. Taf. 159, figs 793–796.

Type locality: [S France].

Current status: species.

***elachista* (Dannehl, 1927)**

Original combination: Lycaena dolus Hb. elachista

In: Dannehl F (1927) Neue Formen und geografische Rassen aus meinem Rhopaloceren-Ausbeuten der letzten Jaren. Mitteilungen der Münchner Entomologischen Gesellschaft 17: 7-8.

Type locality: “Mt. Sabini (Gennaro), Simbruini, Velino, Sirente,… bei Aquila, Gran-Sasso, Morrone, Agatone, Majella” [Central and South Italy].

Current status: synonym of Polyommatus (Agrodiaetus) dolus virgilius (after Eckweiler and Bozano 2016).

***eleniae* Coutsis & De Prins, 2005**

Original combination: Polyommatus (Agrodiaetus) eleniae

In: Coutsis J, De Prins J (2005) A new brown Polyommatus (Agrodiaetus) from northern Greece (Lepidoptera: Lycaenidae). Phegea 33(4): 129–137.

Type locality: Greece, Makedonía, Dráma district, Mt. Falakró, eastern foothills, near Granítis, 900 m.

Holotype in Zöologisch Museum, Universiteit van Amsterdam, Netherlands.

Current status: subspecies of Polyommatus (Agrodiaetus) orphicus.

***epidolus* (Boisduval, 1840)**

Original combination: *Lycaena epidolus*

In: Boisduval [JBA] (1840): Genera et Index Methodictis Europaeorum Lepidopterorurn, p. 13.

Type locality: “Turcia” [Turkey].

Lectotype in The Natural History Museum, London (after Bálint 1999: 28)

Current status: synonym of Polyommatus (Agrodiaetus) menalcas (after: Bálint 1999: 28; Eckweiler and Bozano 2016).

***eriwanensis* (Forster, 1960)**

Original combination: *Agrodiaetus ripartii eriwanensis*

In: Forster W (1960) Einige neue Formen der Gattung *Agrodiaetus* Scudd. (Lep. Lycaen.). Entomologische Zeitschrift 70(3): 19.

Type locality: “Eriwan” [Armenia, Yerevan].

Holotype in Zoologische Staatssammlung, München, Germany (after Häuser and Eckweiler 1997).

Current status: species.

***exuberans* (Verity, 1926)**

Original combination: Hirsutina admetus race exuberans

In: Verity R (1926) Zygaenae, Grypocera and Rhopalocera of the Cottian Alps compared with other races. The Entomologist’s Record and Journal of Variation 38(9): 121.

Type locality: “Oulx” [Italy: Piemonte: Torino].

Syntypes in Museo Zoologico de la Specola, Firenze, Italy (after Häuser and Eckweiler 1997).

Current status: synonym of Polyommatus (Agrodiaetus) ripartii ripartii (after Vila et al. 2010).

***fabressei* (Oberthür, 1910)**

Original combination: *Lycaena rippertii fabressei*

In: Oberthür C (1910) Notes pour servir à établir la Faune Franςaise et Algérienne des Lepidopteres (suite). Etudes de Lépidoptérologié Comparee 4(1): 260.

Type locality: “à Albarracin” [Spain: Teruel].

Lectotype in The Natural History Museum, London (after Bálint 1999: 30).

Current status: species.

***fulgens* (De Sagarra, 1925)**

Original combination: Hirsutina dolus rassa fulgens

In: De Sagarra I (1925) Anotacions a la lepidopterologia ibèrica III (1). Formes noves dignes d’esment. Butlleti de la Institución Catalana de Historia Natural 5(9): 271.

Type locality: “Santa Coloma de Queralt” [Spain: Cataluňa].

Holotype in Museu de Catalunya, Barcelona, Spain (after Häuser and Eckweiler 1997).

Current status: species.

***galloi* (Balletto & Toso, 1979)**

Original combination: *Agrodiaetus galloi*

In: Balletto E, Toso G (1979) On a new species of *Agrodiaetus* (Lycaenidae) from Southern Italy. Nota Lepidopterologica 2(1/2): 13–25.

Type locality: Pollino, Lucania, Southern Italy, loc. Piano di Ruggio, 1550 m.

Holotype in Museo Civico di Storia Naturale Giacomo Doria, Genova, Italy.

Current status: synonym of Polyommatus (Agrodiaetus) ripartii ripartii (after Vila et al. 2010).

***gargano* (Wimmers, 1931)**

Original combination: Lycaena dolus var. gargano

In: Wimmers C (1931) Aus der Lepidopteren-Fauna Italiens (Apulien). Entomologische Zeitschrift 45(7): 96.

Type locality: “Mte. Gargano” [Italy: Puglia: Foggia].

Current status: subspecies of Polyommatus (Agrodiaetus) dolus (after Eckweiler and Bozano 2016).

***humedasae* (Toso & Balletto, 1976)**

Original combination: *Agrodiaetus humedasae*

In: Toso G, Balletto E (1976) Una nuova specie del genere *Agrodiaetus* Hübn. (Lepidoptera Lycaenidae). Annali del Museo Civico di Storia Naturale Giacomo Doria 81: 125.

Type locality: dintorni di Cogne, Val d'Aosta [Italy: Valle d'Aosta].

Holotype in Museo Civico di Storia Naturale “Giacomo Doria”, Genova, Italy (after Häuser and Eckweiler 1997).

Current status: species.

***infralunulata* (Verity, 1943)**

Original combination: *Agrodiaetus dolus infralunulata*

In: Verity R (1943) Farfalle diurne d’Italia 2: 323.

Type locality: not stated.

Syntypes possibly in The Natural History Museum, London (after Häuser and Eckweiler 1997).

Current status: synonym of Polyommatus (Agrodiaetus) dolus virgilius (after Eckweiler and Bozano 2016).

***interjectus* (de Lesse, 1960)**

Original combination: *Agrodiaetus interjectus*

In: de Lesse H (1960) Les nombres de chromosomes dans la classification du groupe d'*Agrodiaetus ripartii* Freyer (Lepidoptera Lycaenidae). Revue Franςaise d'Entomologie 27(3): 253.

Type locality: “Erzincan ... 1200 m” [Turkey: Erzincan].

Holotype in Museum National d’Histoire Naturelle, Paris, France (after Häuser and Eckweiler 1997).

Current status: species.

***iris* (Agenjo, [1973])**

Original combination: Plebejus (Agrodiaetus) damon iris

In: Agenjo R (1971) Nuevas subespecies de ropalóceros ibéricos. Graellsia 26: 30.

Type locality: “Puerto de Pozazal, a 1 001 m., en Valdeprado del Río, provincia de Santander” [Spain: Santander].

Holotype in coll. G. Pardo, Torrelavega (after Häuser and Eckweiler 1997).

Current status: synonym of Polyommatus (Agrodiaetus) fulgens pseudovirgilia (after Eckweiler and Bozano 2016).

***karacetinae* (Lukhtanov & Dantchenko, 2002)**

Original combination: *Agrodiaetus alcestis karacetinae*

In: Lukhtanov V, Dantchenko A (2002) Description of new taxa of the genus *Agrodiaetus* (Hübner, 1822) based on karyotype investigation. Atalanta 33(1/2): 81–107.

Type locality: Turkey, Hakkari, Dez Valley, 1500 m.

Holotype in Institute of Systematic and Population Biology (Zoological Museum), Amsterdam, Netherlands.

Current status: species.

***khorasanensis* (Carbonell, 2001)**

Original combination: *Agrodiaetus khorasanensis*

In: Carbonell F (2001) Contribution à la connaissance du genre *Agrodiaetus* Hübner (1822), *Agrodiaetus ahmadi* et *Agrodiaetus khorasanensis* nouvelles espèces dans le Nord de l’Iran (Lepidoptera, Lycaenidae). Linneana Belgica 18: 105–110.

Type locality: North-East Iran, Khorasan.

Holotype in Muséum National d’Histoire Naturelle in Paris.

Current status: species.

***lefebvrii* (Godart, [1824])**

Original combination: *Polyommatus lefebvrii*

In: Latreille [PA], Godart [J B] (1819[-1824]) Encyclopédie Métho-dique. Histoire Naturelle. 9. Entomologie, ou Histoire Naturelle des Crustaces, des Arachnides et des Insectes, p. 696.

Type locality: “aux environs de Toulon ... et dans les Bouches-du-Rhône” [France: Var/Bouches-du-Rhône].

Syntypes in [Toulon] and [Bouches-du-Rhône] [?] (after Häuser and Eckweiler 1997).

Current status: synonym of Polyommatus (Agrodiaetus) dolus dolus (after Eckweiler and Bozano 2016).

***leonensis* (Verhulst, 2004)**

Original combination: *Agrodiaetus ainsae leonensis*

In: Verhulst J (2004) Description d’une nouvelle sous-espèce d’*Agrodiaetus ainsae* Forster, 1961 provenant de la province de Leon (Lep. Lycaenidae). Linneana Belgica 19(5): 209–212.

Type locality: the vicity of Cistierna, Leon province, northern Spain.

Holotype in Institut des Sciences Naturalles de Bruxelles (after Verhulst 2004).

Current status: subspecies of Polyommatus (Agrodiaetus) fulgens (after Eckweiler and Bozano 2016).

***lorestanus* Eckweiler, 1997**

Original combination: Polyommatus (Agrodiaetus) demavendi lorestanus

In: Eckweiler W (1997) Neue Taxa von Polyommatus (Agrodiaetus) (Lepidoptera: Lycaenidae). Nachrichten des Entomologischen Vereins Apollo Supplementum 16: 8.

Type locality: “Iran, Lorestan, Dorud, Saravand, 2000-2300 m” [Iran: Zagros mts.].

Holotype in coll. W. Eckweiler in Naturkundemuseum, Karlsruhe, Germany.

Current status: subspecies of Polyommatus (Agrodiaetus) demavendi (after Eckweiler and Bozano 2016).

***magnabrillata*** (**Gomez-Bustillo, 1971)**

Original combination: *Plebejus dolus magnabrillata*

In: Gomez-Bustillo M (1971) For un mejor conosimiento de los ropaloceros españoles. Sociedad de Ciencias Naturales Aranzadi, Publicacion N° 19: 8

Type locality: “Puerto de Pozazal (1000 m), T. M. de Valdeprado, Prov. de Santander” [Spain: Santander].

Holotype in coll. M. Gomez-Bustillo, Madrid (after Häuser and Eckweiler 1997).

Current status: synonym of Polyommatus (Agrodiaetus) dolus pseudovirgilia (after Eckweiler and Bozano 2016).

***malievi* (Dantchenko & Lukhtanov, 2005)**

Original combination: *Agrodiaetis admetus malievi*

In: Dantchenko A, Lukhtanov V (2005) New taxa of the brown species-complex of the genus *Agrodiaetus* Hübner, (1822) from Transcaucasia (Lepidoptera, Lycaenidae). Atalanta 35: 327–334, 472–475.

Type locality: Azerbaijan, Talysh mts.

Holotype in Museum of Comparative Zoology, Harvard University, Cambridge, MA, USA.

Current status: subspecies of Polyommatus (Agrodiaetus) yeranyani.

***masul* Lukhtanov, Dantchenko, Vishnevskaya & Saifitdinova, 2015**

Original combination: Polyommatus (Agrodiaetus) rjabovianus masul

In: Lukhtanov V, Dantchenko A, Vishnevskaya M, Saifitdinova A (2015) Detecting cryptic species in sympatry and allopatry: analysis of hidden diversity in Polyommatus (Agrodiaetus) butterflies (Lepidoptera: Lycaenidae). Biological Journal of the Linnean Society 116: 468–485.

Type locality: North Iran, Gilan, vicinity of Masuleh, 2200 m.

Holotype in Zoological Institute of the Russian Academy of Science, St. Petersburg, Russia.

Current status: subspecies of Polyommatus (Agrodiaetus) rjabovianus.

***menalcas* (Freyer, [1837])**

Original combination: *Lycaena Menalcas*

In: Freyer C ([1836]-1839) Neuere Beiträge zur Schmetterlingskunde. 3. Band, 38. Heft, p. 46; tab. 223, figs 2–3.

Type locality: “bei Konstantinopel” [Turkey: Istanbul].

Lectotype in The Natural History Museum, London (after Bálint 1999: 43).

Current status: species.

***montanesa* (Gomez-Bustillo, 1971)**

Original combination: *Plebejus rippartii montanesa*

In: Gomez-Bustillo M (1971) Por un mejor conosimiento de los ropaloceros espanoles. Sociedad de Ciencias Naturales Aranzadi, Publicacion N° 19: 8.

Type locality: “T. M. de Valdeprado, Prov. de Santander” [Spain: Santander].

Holotype in coll. M. Gomez-Bustillo,Madrid [?] (after Häuser and Eckweiler 1997).

Current status: synonym of Polyommatus (Agrodiaetus) ripartii (after Eckweiler and Bozano 2016).

***mozuelica* (Agenjo, [1973])**

Original combination: Plebejus (Agrodiaetus) ripartii mozuelica

In: Agenjo R (1971 [1973]) Nuevas subespecies de ropalóceros ibéricos. Graellsia 26: 31.

Type locality: “Mozuelos, at 840 m., provincia de Burgos” [Spain: Burgos].

Holotype in National Institute of Entomology (Institute Español de Entomologia), Madrid, Spain (after Häuser and Eckweiler 1997).

Current status: synonym of Polyommatus (Agrodiaetus) ripartii ripartii (after Eckweiler and Bozano 2016).

***nephohiptamenos* (Brown & Coutsis, 1978)**

Original combination: *Agrodiaetus nephohiptamenos*

In: Brown J, Coutsis JG (1978) Two newly discovered lycaenid butterflies (Lepidoptera: Lycaenidae) from Greece, with notes on allied species. Entomologist's Gazette 29(4): 207.

Type locality: “mountains of NE. Greece, 1800 m” [Greece].

Holotype in coll. J. Brown, Sutton (after Häuser and Eckweiler 1997).

Current status: species.

***obsoleta* (Stauder, 1921)**

Original combination: Lycaena dolus virgilia f.n. obsoleta

In: Stauder (1921) Deutsche Entomologische Zeitschrift [Iris] 35: 31

Type locality: “Unteritalien” [South Italy].

Syntypes [possibly in BMNH, London] (after Häuser and Eckweiler 1997).

Current status: synonym of Polyommatus (Agrodiaetus) dolus virgilius (after Eckweiler and Bozano 2016).

***orphicus* Kolev, 2005**

Original combination *Polyommatus dantchenkoi orphicus*

In: Kolev Z (2005) *Polyommatus dantchenkoi* (Lukhtanov et Wiemers, 2003) tentatively identified as new to Europe, with a description of a new taxon from the Balkan Peninsula (Lycaenidae). Nota lepidopterologica 28(1): 25–34.

Type locality: South Bulgaria, Rhodopi mts, Hvoyna.

Holotype in National Museum of Natural History, Sofia, Bulgaria.

Current status: species.

***ovchinnikovi* (Lukhtanov & Dantchenko, 2002)**

Original combination: *Agrodiaetus ripartii ovchinnikovi*

In: Lukhtanov V, Dantchenko A (2002) Descriptions of new taxa of the genus *Agrodiaetus* Hübner , [1822] based on the karyotype investigation (Lepidoptera, Lycaenidae). Atalanta 33(1/2): 81–107.

Type locality: Kazakhstan, Vostochno-Kazakhstanskaya oblast’, Zyryanovskij raion, Kremnyukha, 450 m.

Holotype in Zoological Institute of the Russian Academy of Science, St. Petersburg, Russia.

Current status: synonym of Polyommatus (Agrodiaetus) ripartii ripartii.

***paralcestis* (Forster, 1960)**

Original combination: *Agrodiaetus ripartii paralcestis*

In: Forster W (1960) Einige neue Formen der Gattung *Agrodiaetus* Scudd. (Lep. Lycaen.). Entomologische Zeitschrift 70(3): 17.

Type locality: “Akschehir, Sultan Dagh 1700-2200 m” [Turkey: Konya].

Holotype in Zoologische Staatssammlung, München, Germany (after Häuser and Eckweiler 1997).

Current status: subspecies of Polyommatus (Agrodiaetus) ripartii.

***paravirgilius* (Verity, 1943)**

Original combination: Agrodiaetus dolus sattorazza paravirgilia

In: Verity R (1943) Farfalle diurne d’Italia 2: 325.

Type locality: “Monte Faito della penisola Sorrentina” [S. Italy, Sorrento peninsula].

Current status: subspecies of Polyommatus (Agrodiaetus) dolus (after Eckweiler and Bozano 2016).

***paucipuncta* Lhomme, 1927**

Original combination: *Polyommatus dolus paucipuncta*

In: Lhomme (1927) Amateur De Papillons 3: 192.

Type locality: not stated.

Current status: synonym of Polyommatus (Agrodiaetus) dolus (after Eckweiler and Bozano 2016).

***pelopi* (Brown, 1976)**

Original combination: *Agrodiaetus ripartii pelopi*

In: Brown J (1976) On two previously undescribed subspecies of Lycaenidae (Lepidoptera) from Greece. Entomologische Berichten 36(3): 47.

Type locality: Troupa, Mt. Chelmos, Greece, 1300 m [Greece: Peloponnese].

Holotype in coll. J. Brown, Sutton (after Häuser and Eckweiler 1997).

Current status: subspecies of Polyommatus (Agrodiaetus) ripartii.

***pseudorjabovi* Lukhtanov, Dantchenko, Vishnevskaya & Saifitdinova, 2015**

Original combination: Polyommatus (Agrodiaetus) pseudorjabovi

In: Lukhtanov V, Dantchenko A, Vishnevskaya M, Saifitdinova A (2015) Detecting cryptic species in sympatry and allopatry: analysis of hidden diversity in Polyommatus (Agrodiaetus) butterflies (Lepidoptera: Lycaenidae). Biological Journal of the Linnean Society 116: 468–485.

Type locality: Azerbaijan, Talysh mt., Zuvand plateau, vicinity of Mistan village.

Holotype in Zoological Institute of the Russian Academy of Science, St. Petersburg.

Current status: species.

***pseudovirgilia* (de Lesse, 1962)**

Original combination: *Agrodiaetus dolus pseudovirgilia*

In: de Lesse H (1962) Variation chromosomique chez *Agrodiaetus dolus* HB. (Lep. Lycaenidae). Alexanor 2(7): 285.

Type locality: “25 km W. Burgos, près Villanueva de Aragon” [Spain: Burgos].

Holotype in Museum National d’Histoire Naturelle, Paris, France (after Häuser and Eckweiler 1997).

Current status: subspecies of Polyommatus (Agrodiaetus) fulgens.

***punctigera* (Dannehl, 1927)**

Original combination: Lycaena dolus Hb. punctigera

In: Dannehl F (1927) Neue Formen und geografische Rassen aus meinem Rhopaloceren-Ausbeuten der letzten Jaren. Mitteilungen der Münchner Entomologischen Gesellschaft 17: 7–8.

Type locality: “Mt. Sabini (Gennaro), Simbruini, Velino, Sirente,… bei Aquila, Gran-Sasso, Morrone, Agatone, Majella” [Central and South Italy].

Current status: synonym of Polyommatus (Agrodiaetus) dolus virgilius (after Eckweiler and Bozano 2016).

***ramonagenjo* Koçak & Kemal, 2001**

Original combination: *Polyommatus* (s. str. (Agrodiaetus (Admetusia)) ripartii ssp. ramonagenjo

In: Koçak A, Kemal M (2001) *Polyommatus* Latr. Cinsideki *Agrodiaetus* Seksiyonunun Biyolojik Çestiligi Zoocografyasi ve Taksonomisi Üzerine bir Arastirma (Lycaenidae, Lepidoptera). CESA Miscellaneous Papers 78/79: 1–11.

Current status: a replacement name for *Agrodiaetus admetus agenjoi* nec Polyommatus escheri race/form agenjoi Higgins, 1948. Synonym of *Polyommatus ripartii ripartii* (Vila et al. 2010) or separate subspecies of *Polyommatus ripartii* (Eckweiler and Bozano 2016).

***ripartii* (Freyer, 1830)**

Original combination: *Lycaena ripartii*

In: Freyer C (1830) Beiträge zur Geschichte europäischer Schmetterlinge mit Abbildungen nach der Natur, 3. Band, 23. Heft: 128; tab. 133, fig. 3.

Type locality: “Spanien” [Spain].

Lectotype in The Natural History Museum, London (after Bálint 1999: 54).

Current status: species.

***rippertii* (Boisduval, 1832)**

Original combination: *Polyommatus rippertii*

In: Boisduval [JBA] (1832-1843) Icones historique des Lépidoptères nouveaux ou peu connus 1 : 68. Pl. 16, figs 4–6.

Type locality: “aux environs de Digne” [France: Alpes de Haute Provence].

Lectotype in The Natural History Museum, London (after Bálint 1999: 54).

Current status: synonym of Polyommatus (Agrodiaetus) ripartii (Bálint 1999: 54, Eckweiler and Bozano 2016).

***rjabovi* (Forster, 1960)**

Original combination: *Agrodiaetus rjabovi*

In: Forster W (1960) *Agrodiaetus rjabovi* sp. n. Entomologische Zeitschrift 70(14): 157.

Type locality: “Talysh, distr. Lenkoran, Ljulakeran” [Azerbaijan].

Holotype in Zoologische Staatssammlung, München, Germany (after Häuser and Eckweiler 1997).

Current status: invalid name, junior secondary homomym of *Polyommatus thersites rjabovi* Obraztsov, 1936, replaced by Polyommatus (Agrodiaetus) rjabovianus.

***rjabovianus* (Koҫak, 1980)**

Original combination: *Agrodiaetus valiabadi* nom. n. *rjabovianus*

In: Koҫak A (1980) On the nomenclature of some genus- and species-group names of Lepidoptera. Nota lepidopterologica 2(4): 142.

Type locality: see *rjabovi* Forster, 1960.

Type material: see *rjabovi* Forster, 1960.

Current status: Replacement name for *Agrodiaetus rjabovi* Forster, 1960.

***rufomaculata* (Dannehl, 1927)**

Original combination: *Lycaena dolus* Hb. ♀ *rufomaculata*

In: Dannehl F (1927) Neue Formen und geografische Rassen aus meinem Rhopaloceren-Ausbeuten der letzten Jahren. Mitteilungen der Münchner Entomologischen Gesellschaft 17: 7–8.

Type locality: “Mt. Sabini (Gennaro), Simbruini, Velino, Sirente,… bei Aquila, Gran-Sasso, Morrone, Agatone, Majella” [Central and South Italy].

Current status: synonym of Polyommatus (Agrodiaetus) dolus virgilius (after Eckweiler and Bozano 2016).

***sarkani* (Lukhtanov & Dantchenko, 2002)**

Original combination: *Agrodiaetus ripartii sarkani*

In: Lukhtanov V, Dantchenko A (2002) Descriptions of new taxa of the genus *Agrodiaetus* Hübner, [1822] based on the karyotype investigation (Lepidoptera, Lycaenidae). Atalanta 33 (1/2): 81–107.

Type locality: Kazakhstan, Taldy-Kurganskaya oblast’, Dzhungarian Alatau, Andreevskij rayon (Kabanbai), Kolbai vic., 800 m.

Holotype in Museum of Comparative Zoology, Harvard University, Cambridge, MA, USA.

Current status: synonym of Polyommatus (Agrodiaetus) ripartii ripartii (after Vila et al. 2010)

***splendens* (Gomez-Bustillo, 1971)**

Original combination: Plebejus damon subsp. splendens

In: Gomez-Bustillo M (1971) For un mejor conosimiento de los ropaloceros españoles. Sociedad de Ciencias Naturales Aranzadi, Publicacion N° 19: 6.

Type locality: “Puerto de Pozazal (1000 m), T. M. de Valdeprado, Prov. de Santander” [SPAIN: Santander].

Holotype in coll. M. Gomez-Bustillo, Madrid [?] (after Häuser and Eckweiler 1997).

Current status: synonym of Polyommatus (Agrodiaetus) fulgens pseudovirgilia (after Eckweiler and Bozano 2016).

***splendida* (Dannehl, 1927)**

Original combination: Lycaena dolus Hb. splendida

In: Dannehl F (1927) Neue Formen und geografische Rassen aus meinem Rhopaloceren-Ausbeuten der letzten Jaren. Mitteilungen der Münchner Entomologischen Gesellschaft 17: 7–8. Type locality: “Mt. Sabini (Gennaro), Simbruini, Velino, Sirente,… bei Aquila, Gran-Sasso, Morrone, Agatone, Majella” [Central and South Italy].

Syntypes [possibly in BMNH, London] (after Häuser and Eckweiler 1997).

Current status: synomym of Polyommatus (Agrodiaetus) dolus virgilius (after Eckweiler and Bozano 2016).

***subbaeticus*** (Felipe Gil-T. & Talia Gil-Uceda, 2005)

Original combination: *Agrodiaetus fabressei subbaeticus*

In: Felipe Gil-T, Talia Gil-Uceda (2005) *Agrodiaetus violetae* (Gómez-Bustillo, Expósito et Martínez, 1979): morfología comparada y descripción de *Agrodiaetus fabressei subbaeticus* ssp. n. del sureste de la península Ibérica (Lepidoptera, Lycaenidae). Boletín Sociedad Entomológica Aragonesa 36(4): 361.

Type locality: Spain, Iberica peninsula, N. Sierra de la Sagra.

Holotype in coll. of Dr. U. Eitschberger, McGuire Center for Lepidoptera and Biodiversity, Florida Museum of Natural History, University of Florida, USA.

Current status: subspecies of Polyommatus (Agrodiaetus) violetae.

***subtusradiata* (Oberthür, 1910)**

Original combination: *Lycaena Ripertii Subtus radiata*

In: Oberthür (1910) Études de Lépidopterologie Comparee 4: 261.

Type locality: “Fort-Naryne” [Kyrgyzstan].

Current status: unavailable name and synonym of Polyommatus (Agrodiaetus) ripartii colemani (after Eckweiler and Bozano 2016).

***susae* (Bertaccini, 2003)**

Original combination: *Agrodiaetus ripartii susae*

In: Bertaccini E (2003) Prima segnalazione in piemonte di *Agrodiaetus ripartii* (Freyer, 1831) e descrizione di *Agrodiaetus ripartii susae* ssp. nova (Insecta Lepidoptera Lycaenidae). Quoderno di Studi e Notizie di Storio Noturole dello Romogno 17: 127–138.

Type locality: “valle di susa (To) loc. Mompantero”, Italy.

Current status: synonym of Polyommatus (Agrodiaetus) ripartii ripartii.

***tengritaghicus* Koҫak & Kemal, 2001**

Original combination: Polyommatus (Admetusia) tengritaghicus

In: Koҫak A, Kemal M (2001) Lepidoptera Cograpiyesi llstide tetqiqatlar. 2. Qazaqistan Kepinekliring Zoocograpiyesi ve Taksonomiyesi Llstide Tetqiqatlar. (Lepidoptera, Papilionoidea, Hesperioidea). Priamus 10: 111–163.

Type locality: Kazakhstan.

Current status: likely subspecies of Polyommatus (Agrodiaetus) ripartii.

***timfristos* Lukhtanov, Vishnevskaya & Shapoval, sp. n.**

Original combination: *Polyommatus timfristos*

In the present paper.

Type locality: Greece, Timfristos Mt, Karpenisi, 38°55.460'N; 21°47.605 E, 1270 m.

Holotype in Zoological Institute of the Russian Academy of Science, St. Petersburg.

Current status: species.

***urmiaensis* Schurian & Ten Hagen, 2003**

Original combination: Polyommatus (Agrodiaetus) urmiaensis

In: Schurian K, Ten Hagen W (2003) Polyommatus (Agrodiaetus) urmiaensis sp. n. aus Nordwestiran (Lepidoptera: Lycaenidae). Nachrichten des Entomologischen Vereins Apollo 24(1/2): 1–5.

Type locality: Iran, Azarbaygan-e Garbi, vic. Salamas, 60 km N of Orumieh, 1700–1800 m.

Holotype in Senckenberg Museum, Frankfurt am Main, Germany.

Current status: subspecies of Polyommatus (Agrodiaetus) karacetinae.

***valiabadi* (Rose & Schurian, 1977)**

Original combination: Agrodiaetus rjabovi ssp. valiabadi

In: Rose K., Schurian K (1977) Beitraege zur Kenntnis der Rhopaloceren Irans. 7. Beitrag: Eine neue Unterart von *Agrodiaetus rjabovi* Forster. Journal of Entomological Society of Iran 4(1/2): 63.

Type locality: “Elburs-Nordseite (Chalus-Tal), Umgebung Vali-Abad, 1900- 2100 m NN, 25 km nördlich Kandevantunnel” [Iran: Elburs mts.].

Holotype in coll. K. Schurian, Schwalbach [Kelkheim] (after Häuser and Eckweiler 1997).

Current status: species.

***violetae* (Gómez-Bustillo, Expósito & Martínez, 1979)**

Original combination: *Agrodiaetus violetae*

In: Gómez-Bustillo M, Expósito HA, Martínez BP (1979): Una nueva especie para la Ciencia: *Agrodiaetus violetae* (Lep. Lycaenidae). Shilap Revista de Lepidopterología 7(1): 51.

Type locality: “Sierra de Almijara (a 1150 m.), Prov. de Málaga” [Spain: Malaga].

Holotype in coll. M. Gómez-Bustillo, Madrid [?] (after Häuser and Eckweiler 1997)

Current status: species.

***violetapunctata* (Gómez-Bustillo, Exposìto & Martinéz, 1979)**

Original combination: Agrodiaetus violetae f. violetapunctata

In: Gómez-Bustillo M, Expósito HA, Martínez BP (1979): Una nueva especie para la Ciencia: *Agrodiaetus violetae* (Lep. Lycaenidae). Shilap Revista de Lepidopterología 7(1): 53.

Type locality: “Sierra de Almijara 9°1150 m), Prov. de Málaga” [Spain].

Current status: synonym of Polyommatus (Agrodiaetus) violetae.

***virgilius* (Oberthür, 1910)**

Original combination: Lycaena dolus race virgilia

In: Oberthür C (1910) Notes pour servir à établir la Faune Franҫaise et Algérienne des Lépidoptères (suite). Études de Lépidoptérologie Comparée 4(l): 263.

Type locality: “Italic méridionale . . . notamment à Sulmona” [Italy: Abruzzi e Molise: L'Aquila].

Lectotype in The Natural History Museum, London (after Bálint 1999: 63).

Current status: subspecies of Polyommatus (Agrodiaetus) dolus.

***vittatus* (Oberthür, 1892)**

Original combination: *Lycaena dolus* forma geographica *vittata*

In: Oberthür C (1892) Bulletin de la Société Entomologique de France, 1892: X.

Type locality: “Lozère” [France: Lozère].

Syntypes possibly in The Natural History Museum, London (after Häuser and Eckweiler 1997).

Current status: subspecies of Polyommatus (Agrodiaetus) dolus.

***yeranyani* (Dantchenko & Lukhtanov, 2005)**

Original combination: *Agrodiaetus admetus yeranyani*

In: Dantchenko A, Lukhtanov V (2005) New taxa of the brown species-complex of the genus *Agrodiaetus* Hübner, (1822) from Transcaucasia (Lepidoptera, Lycaenidae). Atalanta 35: 327–334, 472–475.

Type locality: Armenia, Zangezur mts, Kajaran distr., right bank Vokhtchi River, Pkhrut vic. 1900 m.

Type material: Museum of Comparative Zoology (Harvard University, Cambridge, MA, USA).

Current status: species.

## Appendix 2

Additional phylogenetic trees

## Appendix 3

Habitats of the studied species
